# Redox proteomic insights into involvement of clathrin-mediated endocytosis in silver nanoparticles toxicity to *Mytilus galloprovincialis*

**DOI:** 10.1371/journal.pone.0205765

**Published:** 2018-10-29

**Authors:** Younes Bouallegui, Ridha Ben Younes, Ridha Oueslati, David Sheehan

**Affiliations:** 1 Research Unit of Immuno-Microbiology Environmental and Carcinogensis, Sciences Faculty of Bizerte, University of Carthage, Bizerte, Tunisia; 2 Proteomic Research Group, School of Biochemistry and Cell Biology, University College Cork, Cork, Ireland; 3 Dept of Chemistry, Khalifa University of Science and Technology, Abu Dhabi, United Arab Emirates; VIT University, INDIA

## Abstract

Clathrin-mediated endocytosis is a major mode of nanoparticle (NP) internalization into cells. However, influence of internalization routes on nanoparticle toxicity is poorly understood. Here, we assess the impact of blocking clathrin-mediated endocytosis upon silver NP (AgNP) toxicity to gills and digestive glands of the mussel *Mytilusgalloprovincialis*using the uptake inhibitor, amantadine. Animals were exposed for 12h to AgNP (< 50 nm) in the presence and absence of amantadine. Labeling of oxidative protein modifications, either thiol oxidation, carbonyl formation or both in two-dimensional electrophoresis separations revealed 16 differentially affected abundance spots. Amongst these, twelve hypothetical proteins were successfully identified by peptide mass fingerprinting (MALDI TOF-MS/MS). The proteins identified are involved in buffering redox status or in cytoprotection. We conclude that blockade of clathrin-mediated endocytosis protected against NP toxicity, suggesting this uptake pathway facilitates toxicity. Lysosomal degradation and autophagy are major mechanisms that might be induced to mitigate NP toxicity.

## Introduction

Many adverse effects exerted by silver nanoparticles (AgNP) on the mussel *Mytilus galloprovincialis*, are directly or indirectly mediated by generation of reactive oxygen species (ROS)[1–3]. Various factors seem to contribute to determining toxicity such as exposure time, particle chemical composition, final concentration and targeted organs[4–8]. Several homeostatic processes that might be induced to protect against redox status have previously been investigated. These include, antioxidant defense enzymes such as catalase (CAT), glutathione peroxidase (GPx), glutathione transferase (GST) and superoxide dismutase (SOD). Cytoprotective mechanisms include immune cell recruitment, apoptosis, autophagy, cytoskeleton modeling/reorganization due to recycling processes, protein refolding/isomerization. In addition, redox effects such as protein carbonylation or disulfide bond formation through thioredoxin systems provide a means of redox buffering to alleviate oxidative stress[8–16].

Third researchers have investigated the possible role of differential uptake routes for nanoparticle internalization in influencing NP toxicity either on short- or long-term exposure[12, 17, 18, 7]. However, the precise involvement of different internalization routes in regulation of NP toxicity remains unclear[12, 17, 18].

Two principal endocytotic routes have been described for NP entry into cells. Such routes involves, either trafficking to endosomal and lysosomal compartments (conventional endocytosis) or, alternatively, cell-surface lipid raft associated domains known as *caveolae*, which avoid the degradative fate of material entering the endosomal/lysosomal system[12]. The endo-lysosomal pathway facilitates intracellular trafficking ultimately ending in fusion with lysosomes to enable breakdown of vesicle contents. Distinct mechanisms have been described for intracellular trafficking to lysosomes: 1. Endocytosis (fluid phase) and 2. Clathrin-dependent endocytosis[19]. Clathrin-dependent endocytosis involves assembly of clathrin (a specific protein coat at the intracellular face of the plasma membrane) in the formation of clathrin-coated pits. These cover a significant fraction (1–2%) of plasma membrane surface and support fast budding of vesicles intracellularly (as quick as 1min) [20–22, 12, 23, 19, 18].

In this same context, it is interesting to note previous studies that reported on the lysosomal-autophagic system (removal site of damaged cellular constituents and redundant products by lysosome contents) as a common target for many environmental pollutants. Lysosomes accumulate many toxic metals and organic xenobiotics (which perturb normal function and damage the lysosomal membrane)[10, 14]. The regulation of this highly conserved group of cellular processes appears to be very similar in eukaryotic organisms ranging from yeasts to Humans and they are often implicated in disease processes, cell death and adaptive responses [10]. Moore et al, [14] documented that autophagy may have a protective role in the context of oxidative stress. Interestingly, failure/overcome of the first line-defense against oxidative damage (antioxidant enzymes defenses) induced lysosomal autophagy which provided a second line of defense against harmful effects of damaged and malfunctioning proteins (by removing oxidatively damaged proteins, inappropriately folded proteins, impaired organelles…etc.,). Autophagy may also serve as third line of defense by triggering programmed cell death to remove irreversibly damaged cells in order to maintain organ integrity, once autophagic capabilities are compromised[12, 13, 10, 14].

The present study aimed to assess the role of clathrin-mediated endocytosis on NP toxicity by probing oxidative stress-related damage. A redox proteomic investigation was performed on gill and digestive gland of *M*. *galloprovincialis* to detect and analyze redox-based changes in the proteome that might be altered by silver nanoparticles (AgNP) and/or changed as post-translational modifications (carbonylation and thiol group oxido-reduction). Effects in cellular turnover/signaling were studied after exposure to AgNP (< 50nm) for 12h prior to and after inhibition of clathrin-mediated endocytosis using the pharmacological inhibitor amantadine hydrochloride as blocker[24].

## Material and methods

### Chemicals and silver nanoparticles

Protein assay dye reagent concentrate was obtained from Bio-Rad (CA, USA); Unstained protein molecular weight markers for SDS-PAGE were from Thermo Scientific (Rockford, IL, USA); 5-Iodoacetamido-fluorescein (IAF), 5-fluorescein thiosemicarbazide (FTSC) and all other general reagents suitable for electrophoresis were purchased from Sigma—Aldrich Ireland Ltd. (Arklow, Co. Wicklow, Ireland); Reagents for proteomic work (Immobiline Drystrips and IPG buffer) were sourced from GE Healthcare.

Poly-vinyl-pyrrolidone (PVP)-coated AgNP (< 50 nm; 99.1% purity) were locally produced using a modified polyol process [25–26] by the chemistry department (FSB, Bizerte, Tunisia). As previously reported [27] TEM analysis (TECNAI G20, Ultra-Twin, FSB, Bizerte, Tunisia) for AgNP <50 nm demonstrated homogeneous spherical characteristics with an approximate size of 50 nm. Size distribution revealed median size of 41.6 ± 18.82 nm. Recorded XRD pattern (D8 Advance diffractometer (Bruker, Bizerte); Bragg—Brentano configuration at 40 kV and 40 mA) from representative batches of silver powder showed the crystalline nature of the AgNP where the diffraction peaks matched the face centered cubic (fcc) phase of silver. UV-Vis spectrum (T60; PG-instruments, Leicestershire, UK) of the colloidal solution provides information on the average particle size, whereas its full width at half-maximum (fwhm) can be used to estimate particle dispersion as previously demonstrated [28]. Agglomeration status analyses performed prior to exposure were confirmed by absorbance spectra at λ_max_ = 400 nm that clearly indicated the AgNP had a homogenous dispersion in aqueous solutions [28].

AgNP stock solutions were prepared and suspended in artificial sea water (ASW; 58.5% NaCl; 26.5% MgCl_2_-6H_2_O; 9.8% Na_2_SO_4_; 2.8% CaCl_2_; 1.65% KCl; 0.5% NaHCO_3_; 0.24% KBr; 0.07% H_3_BO_3_; 0.0095% SrCl_2_-6H_2_O; and, 0.007% NaF; [29]), salinity = 35%, pH 8.0). AgNP stock solution was mixed several times by inversion and an aliquot removed as a working solution that was sonicated for 15 min in alternating cycles (2 x 30 sec) in an ultrasonic bath (100 W; 40 KHz; VWR, Strasbourg, France).

### Experimental design

No specific permissions are required for our study. No endangered or protected species were used in our study. Mussels (*M*. *galloprovincialis*) of average shell length 75 [± 5] mm were collected from Bizerte Lagoon (Northeast Tunisia; 37°08′–37°15′N, 9°48′–9°57′E) and maintained in oxygenated ASW (salinity 35%, pH 8) in static tanks under standard conditions (aeration, photoperiod: 12/12 h; T = 16°C). Animals were acclimated (48 h) and not fed during either acclimation or exposure. The exposure rate in each tank was 1 mussel/0.5 L ASW in all experiments. AgNPs final concentration was set at 100 μg AgNPs/L[30].

As previously described by [31], mussels were separately exposed to AgNP (<50nm) for 12 h—with/without having also been exposed to inhibitor of endocytosis. For the inhibitor-treated groups, mussels (n = 10) were incubated at 100 μM amantadine (Sigma). Control groups (each n = 10) were maintained in oxygenated tanks of only ASW and/or ASW with inhibitor. All exposures were performed in triplicate.

### Animal dissection and homogenate preparation

Digestive gland and gill tissues were dissected from control and treated groups of mussel and then were homogenized (10mM Tris-HCL, pH 7.2; 0.5 M sucrose; 1 mM EDTA; 1 mM PMSF), centrifuged at 20,000xg for 1h and supernatants were stored at -80°C until required.

### Protein quantification

Protein concentrations were measured by the method of Bradford [32]. Protein estimation was performed in quadruplicate in a microplate reader at a wavelength of 595 nm using bovine serum albumin as a standard. Protein content for each tissue (digestive gland and gill) was calculated and recorded.

### Protein thiol and carbonyl labeling

Protein thiols were labeled with IAF [33] (stock solution 20mM IAF in DMSO) by adding prepared tissue homogenates (120μg protein) to a final concentration of 200 μM IAF for 2 h at 4°C in the dark [34]. Proteins were precipitated in a final concentration of 20% (w/v) trichloroacetic acid (TCA) for 5 min at 4°C and centrifuged at 11,000g for 3 min at 4°C. Pellets were re-suspended in 40 μl of water and 500μl of ice-cold acetone (incubated for 2h up to overnight at -20°C) before centrifugation at 11,000g for 3 min at 4°C. Protein carbonyls were labeled by adding FTSC to tissue homogenates (120μg) at a final concentration of 1 mM [34]. Samples were incubated for 2h in the dark, at 4°C before precipitation of proteins with a final concentration of 10% (w/v) TCA. Pellets were washed twice with 500 μl of ice-cold 1:1 ethanol-ethylacetate. Prior to rehydration, pellets were centrifuged and dried, to make sure no solvent remained in the samples. Pellets were dried for 5–10 min to ensure there was no acetone present prior to re-suspension in rehydration buffer. All subsequent steps were carried out with minimal exposure of samples to light.

### Two-dimensional electrophoresis and protein identification

Labeled proteins were re-suspended in rehydration buffer (5M urea, 2M thiourea, 2% w/v CHAPS, 2% v/v IPG buffer). Labeled protein (120μg), was loaded onto an immobiline Drystrip (pH 3–10 nonlinear, 7cm; GE Healthcare), which was rehydrated overnight in the dark. Isoelectric focusing (IEF) was performed on a PROTEAN IEF system (Bio-Rad), according to the strip manufacturer’s recommendations. Strips were reduced in equilibration buffer (6 M urea, 0.375 M Tris, pH 8.8, 2% w/v SDS, 20% v/v glycerol) containing 2% w/v dithiothreitol (DTT) for 20 min and thiols were then blocked with equilibration buffer containing 2.5% w/v iodoacetamide for 20 min. After focusing, strips were loaded into 10% polyacrylamide gels for SDS-PAGE separation. Gels were scanned for fluorescence and then stained with colloidal coomassie. Image analysis was performed using the Progenesis-Same-Spots software (Nonlinear Dynamics Limited, UK). Experiments were defined by compound, with the exposure concentrations representing treatments. Spots were considered of interest when showing a 1.5-fold change between treatments as well as having a p < 0.05 in ANOVA. Significant, well-resolved spots of sufficient intensity were then selected for mass spectrometry(MS) analysis. Features from the fluorescence images were considered interesting only if they could be matched to a coomassie-stained feature of sufficient staining intensity for likely MS detection. Selected spots were excised manually using clean pipette tips and in-gel digested with trypsin according to [35]. Extracted peptides were loaded onto a R2 micro column (RP-C18 equivalent) where they were desalted, concentrated and eluted directly onto a MALDI plate using a-cyano-4-hydroxycinnamic acid as the matrix solution in 50% acetonitrile and 5% formic acid. Mass spectra of the peptides were acquired with positive reflectron MS and MS/MS modes using a MALDI-TOF/TOF MS instrument (Bruker, Daltonics-ultrafleXtreme analyzer) with exclusion list of the trypsin autolysis peaks (842.51, 1045.56, 2211.11 and 2225.12). The collected MS and MS/MS spectra were analyzed in combined mode by using the Mascot search engine (version 2.2; Matrix Science, Boston, MA) and the NCBI database restricted to 1.5 Da peptide mass tolerance for the parent ions, an error of 0.5 Da for the fragments, one missed cleavage in peptide masses, and carbamidomethylation of Cys and oxidation of Met as fixed and variable amino acid modifications, respectively. No taxonomy restrictions were applied. The identified proteins were only considered if a MASCOT score above 95% confidence was obtained (p < 0.05) and at least one peptide was identified with a score above 95% confidence (p < 0.05). This analysis was conducted by the Proteomic Core Service, Institute of Medical Science, University of Aberdeen, Scotland, UK.

### Hypothetical proteins

Hypothetical proteins (HP) identified by MS/MS were studied further using bioinformatics tools to gain insight into their possible biological functions. This was achieved using blastp and DELTA-BLAST from the National Center for Biotechnology Information (NCBI; http://blast.ncbi.nlm.nih.gov/Blast.cgi) to identify sequence similarity with known proteins. In addition, conserved sites, domains and families present in the HP were studied using the Interproscan tool from the European Bioinformatics Institute (EBI; http://www.ebi.ac.uk/Tools/pfa/iprscan/). Results include the highest-scoring, non-hypothetical, protein from blastp, as well as the domains and families identified from Interproscan. In the present study, those were always in accordance with results from DELTA-BLAST.

### Ethical statement

All mussel experiments were carried out following the ARRIVE guidelines and in accordance with the U.K. Animals (Scientific Procedures) Act, 1986 and associated guidelines, EU Directive 2010/63/EU for animal experiments.

## Results

### 2DE

**Fig 1:**
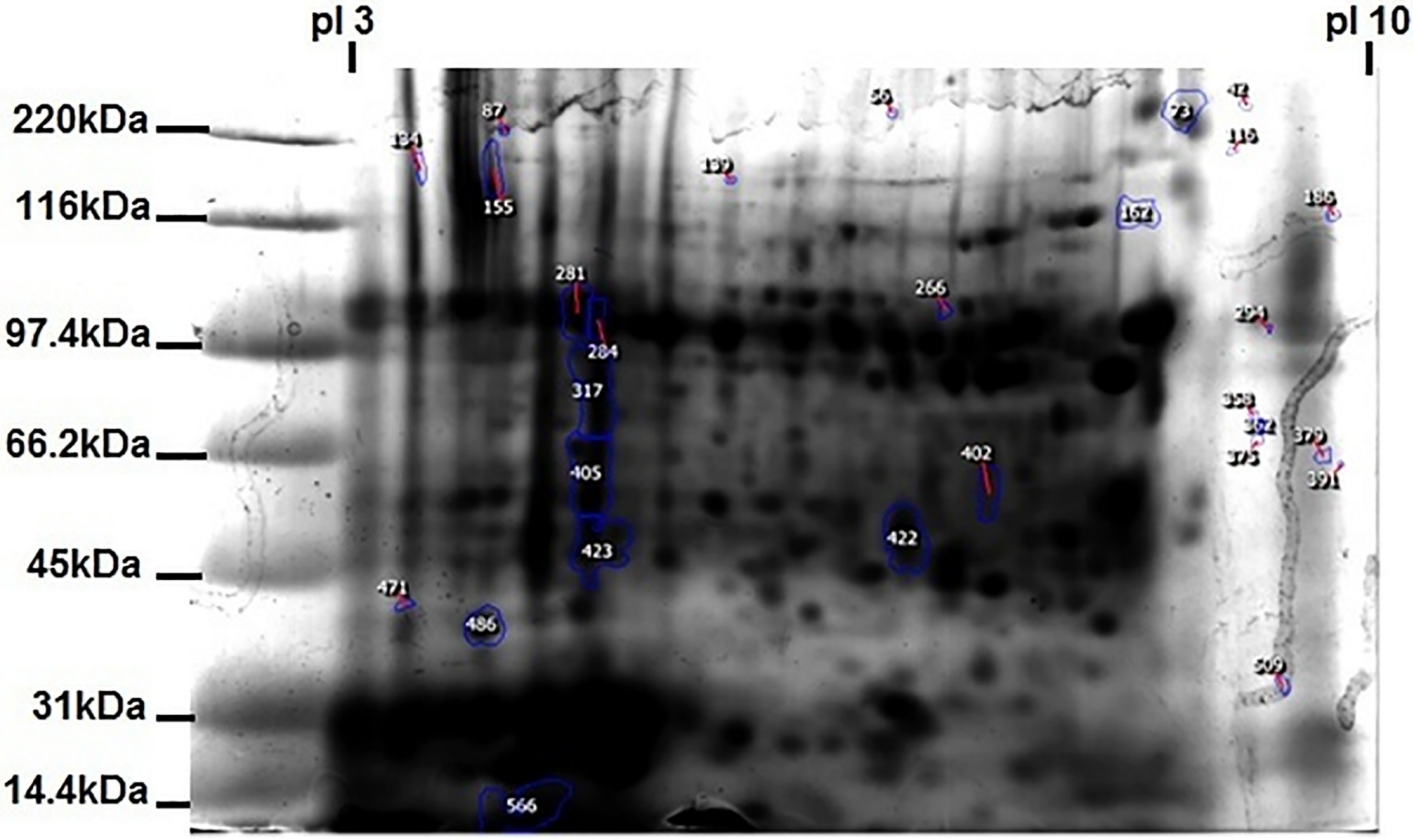
Representative 2DE separation of digestive gland proteins. Spots selected for excision and identification by MALDI TOF-MS/MS are among the numbered ones.

On 2-DE analysis of proteins from gill and digestive gland of mussels exposed to AgNP < 50 nm for 12h prior to and after inhibition of clathrin-mediated endocytosis, a total number of 16 spots showed significant volume change of at least 1.5-fold either in carbonyl content (FTSC fluorescence), thiol content (IAF fluorescence) and/or protein expression level (coomassie staining intensity) (Fig 1).

Labeled separations showed oxidative stress associated with decreased presence of reduced thiols or increased presence of carbonylated proteins. In fact, no significant changes were shown in carbonyl content of protein extract from digestive gland of mussels exposed to AgNP alone or in the presence of amantadine (clathrin-mediated route blocker) for 12h. Only one spot (n° 135) of gill protein recorded significant increase in carbonyls (+3.5 fold) in the presence of amantadine (Table 1). Furthermore, for all digestive gland protein spots, changes were noticed in thiol content including the four significant ones (-2.0, -1.7, 2.2 and 2.4 for spots n° 486, 73, 284 and 281, respectively) when exposed to AgNP alone. However, after clathrin-route blockade (amantadine) all protein extracts (digestive gland and gill) showed significant changes except spot n° 155, 471 for digestive gland and 135 for gill.

**Table 1:** Fold change of spot volumes of identified proteins^a^.

	AgNP< 50 nm			AgNP< 50 nm + Amantadine		
	Carbonyl content (FTSC)	Thiol content (IAF)	Protein content (coomassie)	Carbonyl content (FTSC)	Thiol content (IAF)	Protein content (coomassie)
**Protein (spot)**						
*Digestive gland*						
Chitinase-like protein 3 (155)	--	--	-5.5*	--	--	-1.6*
HSP 70 (162)	--	-1.3	1.5*	--	-2.7*	2.2*
Trypsin (73)	--	-1.7*	5.4*	--	-2.1*	4.2*
Shell myostracum collagen-like protein 1 (281)	--	2.4*	-0.8	--	2.7*	-3.6*
Collagen-like protein-7 (284)	--	2.2*	1.8	--	1.8*	-3.9*
Actin (402)	--	-1.3	2.0*	--	-1.5*	2.3*
Cationic trypsin 3 (405)	--	-0.9	-1.8	--	-2.5*	-2.9*
Actin (422)	--	-1.3	1.1	--	-1.7*	1.8*
Trypsin (471)	--	--	-4.1*	--	--	-1.7
Predicted peptidyl-prolyl cis-trans isomerase (486)	--	-2.0*	-5.0*	--	-2.3*	-1.4
*Gills*						
Trypsin (135)	2.3	--	-2.7*	3.5*	--	1.2
Procollagen-proline dioxygenase βsubunit (223)	--	1.2	-2.1*	--	2.6*	-2.4*

^a^Fold changes are relative to control and given only for treatment where the spot was significantly changed with max fold change ≥ 1.5

*p<0.05(ANOVA)

Among these changes 6 spots (162, 73, 402, 405 and 486) from 9 showed significant decrease in thiol content due to oxidative stress. Other minor changes (max fold ≤ 1.5) also were found (Table 1). In addition, only one spot of gill proteins was revealed to be increased in thiol content (+1.2 for 223) when exposed to AgNP in the presence of amantadine. Otherwise, protein content (coomassie stained spots) indicated changes in the average volume due to alterations in protein expression (Table 1).

### Protein identification

Of the 16 spots of interest (14 from digestive gland and 2 from gill) in 2-DE separations, twelve were successfully identified by MS/MS analysis (Table 2).

**Table 2:** Identified proteins using MALDI TOF-MS/MS.

Spot n°	Accession n°	Protein ID	Organism	Protein Mw (Da)	Mascot score	Predicted P*I*	Matched peptides	Sequence coverage (%)
**135**	gi|136429	Trypsin	*Susscrofa*	25,078	71	7	1	8
**155**	gi|906541877	Chitinase-like protein 3	*Mytilus galloprovincialis*	16,753	74	6.29	2	18
**162**	gi|157679184	HSP 70	*Poeciliareticulata*	69,952	114	5.13	2	2
**223**	gi|390979785	procollagen-proline dioxygenase beta subunit	*Mytilus galloprovincialis*	55,402	539	4.53	9	25
**73**	gi|136429	Trypsin	*Susscrofa*	25,078	73	7	1	8
**281**	gi|891167073	Shell myostracum collagen-like protein 1	*Mytilusgalloprovincialis*	19,529	117	7.79	2	15
**284**	gi|906541868	Collagen-like protein-7	*Mytilusgalloprovincialis*	15,215	164	9.21	5	26
**402**	gi|218932486	Actin	*Trichoderma sulawesense*	23,977	214	4.95	3	16
**405**	gi|554561470	cationic trypsin 3	*MyotisBrandtii*	27,166	74	6.85	1	8
**422**	gi|3328	Actin	*Saccharomces cerevisiae*	41,907	82	5.53	1	4
**471**	gi|136429	Trypsin	*Susscrofa*	25,078	83	7	1	8
**486**	gi|919045645	Predicted peptidyl-prolyl cis-trans isomerase	*Lingulaanatina*	23,267	136	9.35	3	12

Among these, four proteins were found to be from *M*. *galloprovincialis*. The three bioinformatic tools used (blastp, DELTA-BLAST and InterproScan) were in good agreement as to the type of proteins identified. In brief, only the best results from the blastp search and the families and main domains found in InterproScan are included in Table 2. Among the identified spots, four were annotated as trypsin and cationic trypsin 3, two as actin, two as collagen-like protein (shell myostracum 1 and 7) and one each as β subunit of procollagen-proline dioxygenase, Heat shock protein 70 (HSP70), chitinase-like protein 3, and a predicted peptidyl-prolyl *cis-trans* isomerase (Table 2). The fold changes of the spot volumes from which the above proteins were identified, are shown in Table 1 while the features themselves are shown on the reference image of the analysis (Fig 1). In order to gain insight into the potential function of these proteins, bioinformatics tools were used to find similar proteins and the families to which they could be related through conserved function. The results of these analyses are shown in Table 3.

**Table 3:** Blast and interproscan search for hypothetical proteins.

Spot n°	Accession n°	Best hit^a^ (protein [*species*], *E*-value, identity %)	Families and domains^b^	Go functions^b^
**135, 73, 471**	gi|136429	Trypsinogen isoform X1 [*Susscrofa*], *E*: 4×10^−169^, identity: 100%	Peptidase S1A, chymotrypsin family; Peptidase S1, PA clan; Serine proteases, trypsin domain; Serine proteases, trypsin family, histidine active site; Serine proteases, trypsin family, serine active site	Proteolysis, serine-type endopeptidase activity
**155**	gi|906541877	Chitinase like protein3 [*Mytilusgalloprovincialis*], *E*: 9×10^−108^, identity: 100%	Glycoside hydrolase superfamily; glycoside hydrolase family 18, catalytic domain; chitinase II; chtinase insertion domain	Carbohydrate metabolic process, chitin binding
**162**	gi|157679184	HSP70 protein [*Poeciliareticulata*], *E*: 0.0, identity: 100%	Heat shock protein 70 family; Heat shock protein 70kD, peptide-binding domain; Heat shock protein 70kD, C-terminal domain; Heat shock protein 70, conserved site	None predicted.
**223**	gi|390979785	Procollagen-proline dioxygenase β subunit [*Mytilusgalloprovincialis*], *E*: 0.0, identity: 100%	Protein disulphide isomerase; thioredoxin-like fold; thioredoxin domain; disulphide isomerase; thioredoxin, conserved site	Cell redox homeostasis, isomerase activity, endoplasmic reticulum
**281**	gi|891167073	Shell myostracum collagen like protein 1 [*Mytilusgalloprovincialis*], 4×10^−124^, identity: 100%	Van willebrand factor, type A domain	None predicted
**284**	gi|906541868	Collagen like protein7 [*Mytilusgalloprovincialis*], 2×10^−96^, identity: 100%	Van willebrand factor, type A domain	None predicted
**402**	gi|218932486	Actin, partial [*Trichodermasulawesense*], 3×10^−159^, identity: 100%	Actin family; actin like conserved site	None predicted
**405**	gi|554561470	PREDICTED: cationic trypsin3 [*Myotisbrandtii*], *E*: 0.0, identity: 100%	Peptidase S1A, chymotrypsin family; Peptidase S1, PA clan; Serine proteases, trypsin domain; Serine proteases, trypsin family, histidine active site; Serine proteases, trypsin family, serine active site	Proteolysis, serine-type endopeptidase activity
**422**	gi|3328	Actin [*Saccharomyces cerevisiae*], *E*: 0.0, identity: 100%	Actin family; actin conserved site; actin like conserved site	None predicted
**486**	gi|919045645	PREDICTED: peptidylprolyl cis-trans isomerase β like [*Lingulaanatina*], 7×10^−153^, identity: 100%	Cyclophilin-type peptidyl-prolyl cis-trans isomerase; Cyclophilin-like domain; Cyclophilin-type peptidyl-prolyl cis-trans isomerase domain; Cyclophilin-type peptidyl-prolyl cis-trans isomerase, conserved site	Proteinpeptidyl-prolylisomerization, proteinfolding, peptidyl-prolyl cis-trans isomerase activity

^a^Best hit results are obtained using a blastp search.

^b^Families, domains and GO functions were obtained using Interproscan.

## Discussion

### Functions of identified proteins

Bioinformatics searches of proteins identified by MS/MS analyses resulted in the identification of ten hypothetical sequences with similarity to known proteins (Table 3). 2DE separations of labeled proteins from gills and digestive gland revealed differential posttranslational modifications (PTM). In fact, significant changes in thiol content were mainly only found in proteins from digestive gland, whilst carbonyl content changes were found to be significant only in the case of gill proteins. This result suggests differential tissue-specific functioning, which might be related to different redox effects as previously described [36]. Spots 135, 37, 471 (the first was from gill while the other two were from digestive gland) contain the same protein; Trypsinogen isoform X1. This is the same protein family as cationic trypsin 3 identified from spot 405. These four spots represent the same hypothetical protein function; a serine-type protease activity. Serine proteases represent over a third of all known proteolytic enzymes and are implicated in a wide range of physiological and biochemical processes including digestion, coagulation and immunity, homeostatic regulation and protein folding [37].

Spots n° 281 and 284 were identified as collagen-like proteins; shell myostracum collagen-like and collagen-like protein 7, respectively, identified from *M*. *galloprovincialis*, represent a Van Willebrand factor (VWF), type A domain which showed a non-predicted function as result of Interproscan search. [38] identified 53 collagen-like proteins from the insoluble matrix of myostracum of *M*.*galloprovincialis* (with collagen- and or -VWA domain-containing proteins). Collagens collectively are the most abundant protein class in vertebrates where they comprise some 25% of all body protein. They form the scaffold for vertebrate tissues and play numerous roles in processes such as cell migration, tissue morphogenesis and repair and the healthy functioning of joints [39]. VWFs may have previously-unrecognized biological functions, including smooth muscle cell proliferation, tumor cell metastasis, immune cell regulation and angiogenesis through vascular endothelial growth factor (VEGF) receptor 2 (VEGFR 2)-dependent proliferation and migration. In response to pathological stimuli, such as inflammation, the circulatory concentration of VWF increases rapidly [40–41].

Two other spots (n° 402 and 422) were identified as actins with similarities, respectively, to *Trichoderma sulawesense* and *Saccharomyces cerevisiae*. In fact, the two spots were considered as part of the same train due to the widespread presence of actins (i.e., globular and filamentous). The actin cytoskeleton is a ubiquitous protein structure which, when combined with microtubules (formed by α-tubulin polymerization) and intermediate filaments, represents the fundamental components of the cytoskeleton. It also plays crucial roles in generation and maintenance of cell morphology/polarity, endocytosis and intracellular trafficking, contractility, motility and cell division in eukaryotic cells. In cells, the assembly and disassembly of actin filaments, and their organization into functional higher order networks, is under the control of specific signaling pathways[42–43]. In non-muscle cells, actin microfilaments interact with myosin (heavy and light chains) and paramyosin to produce a sliding effect, which is the basis of muscular contraction, as well as many aspects of cell motility and organelle transport[44–45]. Actin is redox-sensitive[16].

A chitinase-like protein 3 (spot n° 155) was also identified. Chitinase-like proteins (CLPs) are lectins produced by multiple cell types combining properties of cytokines and growth factors. Spot n° 486 contained Peptidyl prolyl *cis-trans* isomerase β like (PPIsomerase). The latter is a conserved group of enzymes that catalyze the conversion between *cis* and *trans* conformations of proline-containing peptide bonds. This enzyme family plays critical roles in cell cycle progression, gene expression, cell signaling and proliferation. The PPIsomerase cyclophilin is produced by atherosclerotic lesions and from monocytes and endothelial cells. It promotes a chemotactic response, and vascular smooth muscle cells (VSMC) in inflammatory conditions or in response to increased ROS associated with oxidative stress. It is thought that cell signaling relies on slow conformational interconversions of the backbone of key proteins as exemplified by the prolyl *cis/trans* isomerization, and that prolyl *cis/trans* isomerases serve to integrate temporally and spatially protein conformers with signaling events. The causal relationship between prolyl *cis/trans* isomerization catalysis, malignant transformation and tumor progression remains poorly understood [46–48].

The second significant modified protein from gill was from spot n°223, which showed similarity to procollagen-proline dioxygenase (commonly known as proline hydroxylase). This is a protein disulfide isomerase (PDI) which is a thiol oxidoreductase chaperone of the thioredoxin super-family. PDI roles in ER-related redox homeostasis and signaling are well-studied. Proline hydroxylation is the most prevalent post-translational modification of collagen. PDI exerts effects on thiol oxidation/reduction and isomerization, as well as chaperone effects. PDIs may also regulate thiol-disulfide switches in other cellular locations such as the cell surface and cytosol. Extracellularly, PDIs crucially regulate thiol redox signaling of thrombosis/ platelet activation, (e.g. integrins) and support remodeling during injury repair. PDI effects are orchestrated by expression levels or post-translational modifications [49–52]. Cellular ROS generation usually increases upon ER stress. Accumulation of un/misfolded proteins in the ER lumen is the canonical trigger of the unfolded protein response which is often accompanied by oxidative stress and ROS generation [52].

Spot n°162 containedHSP70, a protein family reported to be constitutively or inducibly associated with stress responses present in most bivalves[53]. HSPs are synthesized by cells in stress conditions such as oxidative stress, radiation, pH, salinity and exposure to xenobiotics. They confer cytoprotection from apoptosis by preventing caspase activation [53–54]. Furthermore, HSPs appear to be essential in other processes such as protein folding, trafficking and aggregation or anomalous reactions that could otherwise impair cell functioning and proteolysis regulation in cells [53]. HSP70 expression changes in response to seasonal change and other environmental factors (reviewed in [54]). In addition, HSPs may also be important immunoregulatory agents[55–56].

### Proteomic approach and differential post-translational modification in 2-DE separations

Spots revealed in 2-DE separations appeared to be significantly affected by both treatments (AgNP prior to and after alteration of clathrin-mediated endocytosis) when compared to control. In fact, trypsin identified from digestive gland protein extract (spots n° 73, 405 and 471) showed in almost all cases significant decrease in free thiol content consistent with thiol oxidation but with no significant changes in carbonyl content accompanied with significant changes in the protein expression. Thiol oxidation was noticed to be intensified after the clathrin-mediated route was inhibited which highlights its protective role against AgNP toxicity. This route is considered as a major route for internalizing particles to be processed (digested) through the endo-lysosomal “conventional” endocytosis, presumably mitigating any AgNP toxic effect. Otherwise, protein expression of the identified trypsins (two forms were identified with slightly differences in MW and p*I*) showed two different profiles with significant increases and decreases that were related to different regulation mechanisms or pathways. After clathrin blockade, a significant increase in carbonyl content was recorded with only slight increase in trypsin expression. This could be explained by a possible increase in caspases-dependent apoptosis after prevention of the cytoprotective role of clathrin-mediated endocytosis. Inhibition of apoptotic processes (mostly linked to HSP70 up-regulation) following ROS generation could trigger accumulation of unfolded/misfolded proteins in the ER. This is especially likely following a depletion of PDI (The ER’s most abundant protein), which was found to be decreased by both treatments notwithstanding its role in free thiol reduction.

These results are in good agreement with those of [16] where *M*. *edulis* PDI was reported as having free thiols in a reduced state confirming its importance as a redox buffer. Furthermore, PDI has been found to associate with misfolded proteins and with endogenous proteins such as procollagen in the ER. As previously reported, PDI has been found to promote matrix/cytoskeletal organization which could explain its expression modification found here. AgNPs-associated injury has previously been documented in mussel gill and digestive gland by [27, 57]. Thus, PDI identification, in the current study, to be associated to procollagen-proline dioxygenase is not surprising. CLPs (spot n° 155) are lectins combining properties of cytokines and growth factors. CLP expression significantly decreased when exposed to AgNP prior to and after clathrin-mediated endocytosis inhibition with a higher effect identified on exposure to AgNP alone.

Actin (spots n° 402 & 422) showed moderate decrease in free thiols when exposed to AgNP alone whilst, in the presence of amantadine, thiol decreases were significantly coupled to increased expression. This increase was significant after inhibition of uptake. Such results may be due to the ability of AgNP to trigger cytoskeletal disruption and disorganization [1]. Ag+ leached from NPs could directly bind to proteins of the cytoskeleton or indirectly via—SH group binding, affect Ca^2+^ homestasis or functioning of specific proteins such as Ca^2+^ ATPases [1].

VWF proteins play roles as adhesive glycoprotein mediating interaction of platelets with collagen in damaged vascular sub-endothelium. This was supported by observed increase in free thiol content reflecting an ability of cells to restore homeostatic equilibrium. [57] reported that AgNP generated an inflammatory response, which could promote angiogenesis through VEGFR 2-dependent proliferation and migration following VWF activation. The observed significant decrease in expression of this protein, after blockade of clathrin uptake route, again highlights a cytoprotective role for this uptake route in NP toxicity.

Finally, PPIsomerases [spot n° 486] expression was strongly decreased (5-fold change) when exposed to AgNP alone, which might be explained by an alteration of its activity following oxidative stress, which is supported further by a significant reduction in its free thiol content.

### Role of blockade of clathrin-mediated endocytosis in protein PTM and differential expression

Subcellular organelles and compartmentalization facilitate rapid, precise and simultaneous control of a wide range of chemical processes in cells. An important element for optimal functioning of processes such as endocytosis is maintenance of an acid pH within vesicle lumens. There is a pattern of decreasing pH going from cytosol (pH 7.0) to early endosomes (pH 6.0), late endosomes (pH 5.9) and lysosomes (pH 5.0). This acidification enables selective activation of lysosomal hydrolases, for example [58–59]. pH gradient dissipation towards more alkaline pH values impacts on protein transport and sorting in endocytic pathways and tends to undermine lysosomal degradation as hydrolases have acidic pH optima [58–59]. Organelle acidification can be purposefully disrupted under unbalanced redox equilibrium status by various agents including excessive oxidation of AgNP resulting in Ag+ release from NP under the pH influence of lysosomes (which favor Ag+ accumulation in the lysosome resulting in lysosomal membrane destabilization; [30]). Overall, the resulting compromised protective role of autophagy could be explained here by its failure under oxidative stress conditions as NPs induce cell injury including damaging autophagy and lysosomal function [14].

## Conclusions

**Fig 2:**
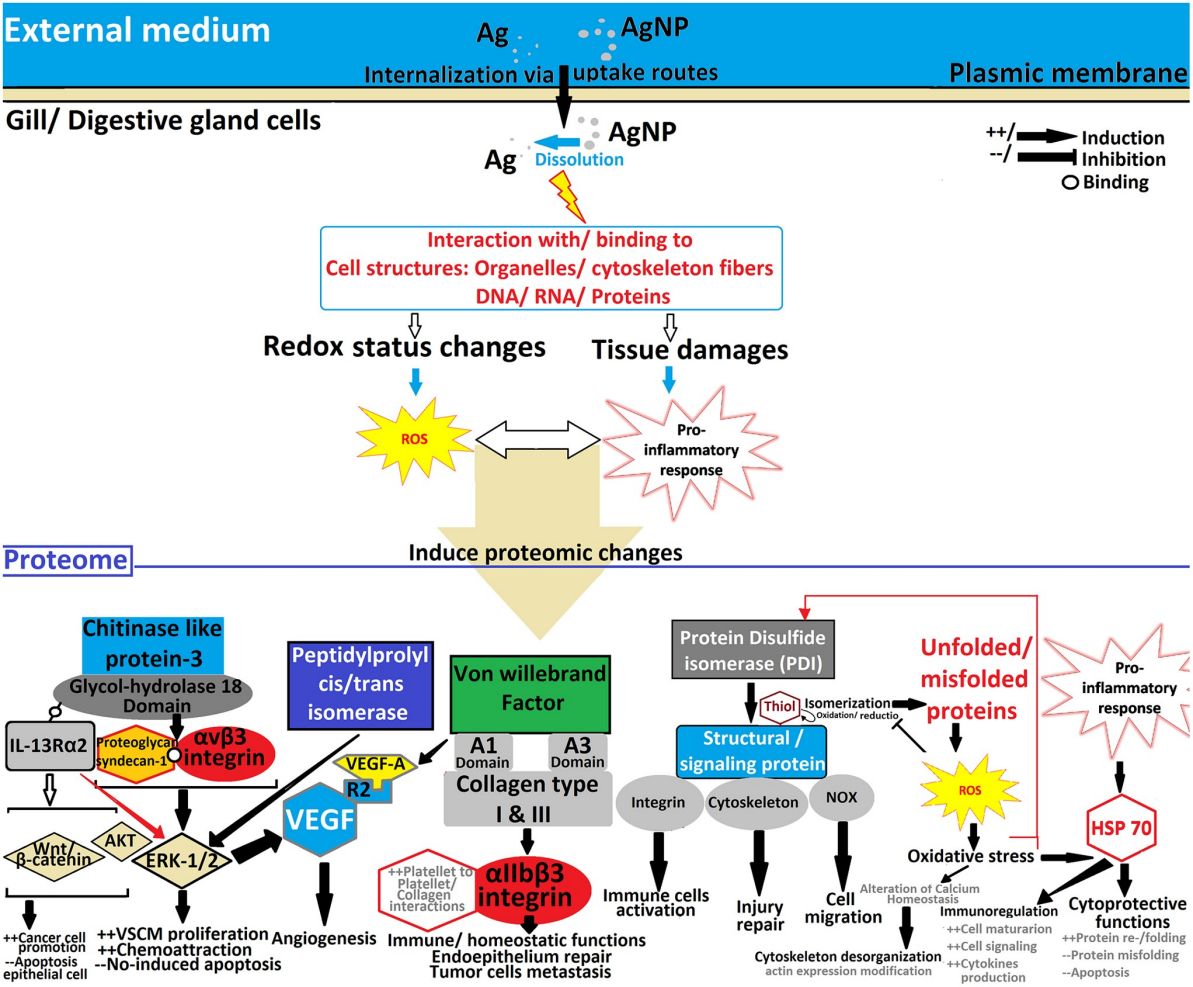
Schema describing the major predicted oxidative status and its related regulation mechanisms induced by AgNP through generation of ROS and inflammatory response.

Overall, the results of the current study report an intensified oxidative stress status after the clathrin-mediated route of NP uptake was inhibited. This highlights a protective role against AgNP toxicity for this pathway, which is considered the major route for trafficking in endo-lysosomal endocytosis. Alteration of the endocytic pathway prevents lysosomal degradation and results in a compromised protective role of autophagy. Otherwise, identified proteins highlighted different interplaying mechanism of regulation (e.g. apoptosis or programmed cell death, immune cell activation, angiogenesis). However, a general model describing the major predicted oxidative situation and its related mechanistic regulation has been drawn in Fig 2, as follows: AgNP induces generation of ROS and inflammatory response, which lead to accumulation of unfolded/misfolded proteins in the endoplasmic reticulum.

This follows depletion/down-regulation of PDI (blockade of uptake route showed the highest effect), alteration in PPIsomerase activity (important down-regulation when exposed to AgNP alone), and up-regulation of chaperon proteins (HSP70). Simultaneously, decreased expression of CLPs (with both exposures; higher effect recorded with AgNP alone) most probably promotes apoptosis, resulting in involvement of buffering molecules as actin and collagen like proteins to induce cytoskeletal reorganization (i.e. tissue repair and/or buffering potential cellular damage).

## Supporting information

S1 Fig Output of the Progenesis same-spot software analyses of 2-dimensional gel electrophoresis featuring comparisons of gel images to the image of reference gel. (PDF)

S1 File Brief description of the contents of supplementary documents S1 Fig and S2 File. (DOCX)

S2 File Output of the Progenesis same-spot software analyses of 2-dimensional gel electrophoresis: Full details of features values showing significant changes. (PDF)
