# Supplementary material for: Redox proteomic insights into involvement of clathrin-mediated endocytosis in silver nanoparticles toxicity to Mytilus galloprovincialis
Source: PLoS One. 2018 Oct 29;13(10):e0205765. doi: 10.1371/journal.pone.0205765 (PMC6205585; doi:10.1371/journal.pone.0205765)
Supplement: S1 Fig — (PDF) [file pone.0205765.s001.pdf]

Comm FTSC Digestive gland

Experiment: Comm FTSC Digestive gland

Report created: 22/12/2015 12:07:14

Reference image

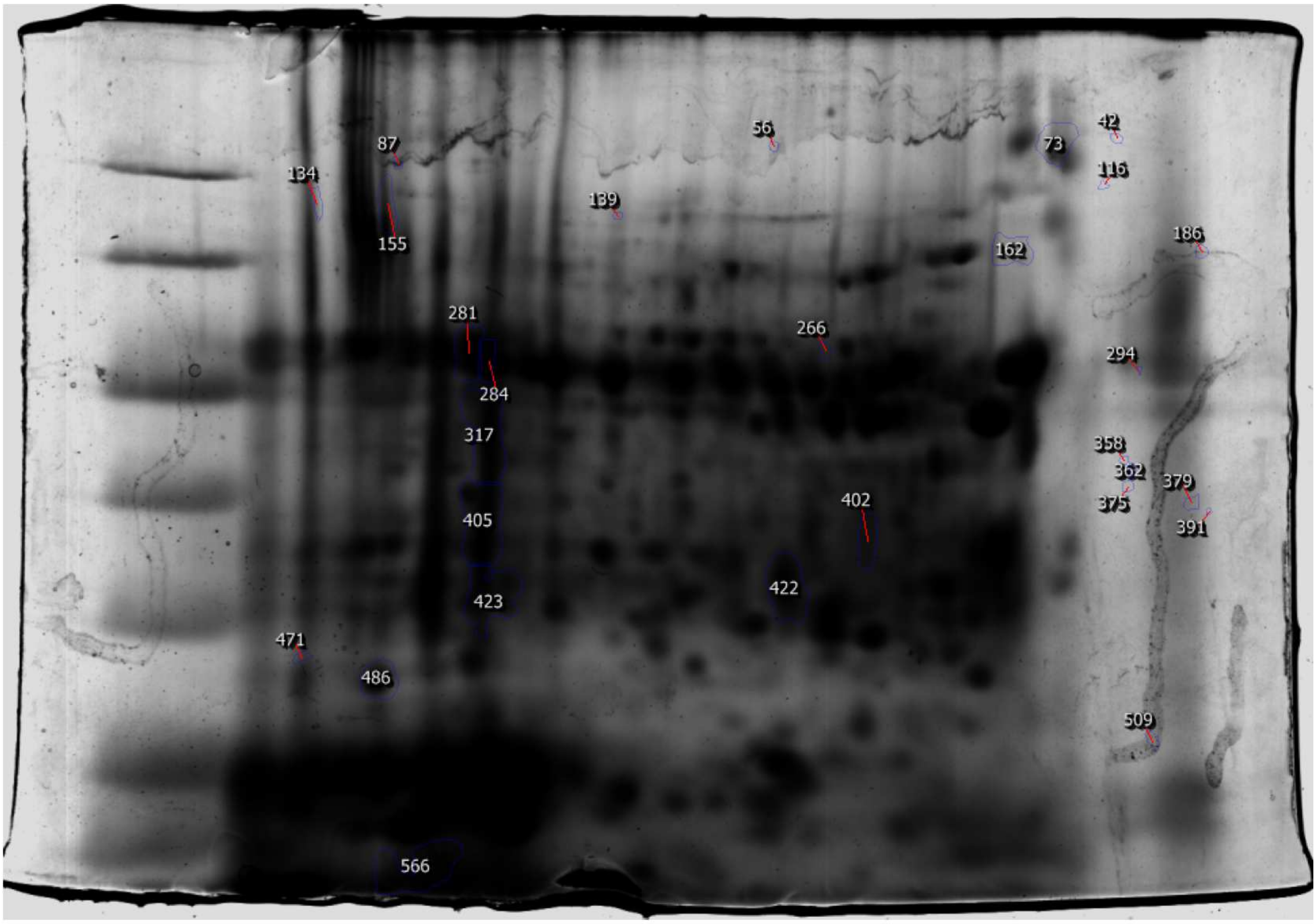

Experiment Design

Condition	Control	Ag50	Ag50+Amant
Replicates	2	2	2

Spots

#	Anova (p)	Fold	Tags	Notes	pI	MW	Protein Accession	Protein Description	Protein pI	Protein MW	Protein URL	Average Normalised Volumes		
												Control	Ag50	Ag50+Amant
266	0.001	3.8										2068.767	7877.707	2323.349
509	0.002	6.1										6909.977	1139.788	4166.555
162	0.003	2.2										2.437e+004	3.666e+004	5.295e+004
139	0.003	2.1										476.292	980.278	476.948
379	0.003	4.5										2519.910	8907.904	1999.094
486	0.008	5.0										8.841e+004	1.770e+004	6.289e+004
362	0.010	4.6										359.683	1649.693	507.505

Comm IAF Digestive gland

Experiment: Comm IAF Digestive gland

Report created: 22/12/2015 11:42:13

Reference image

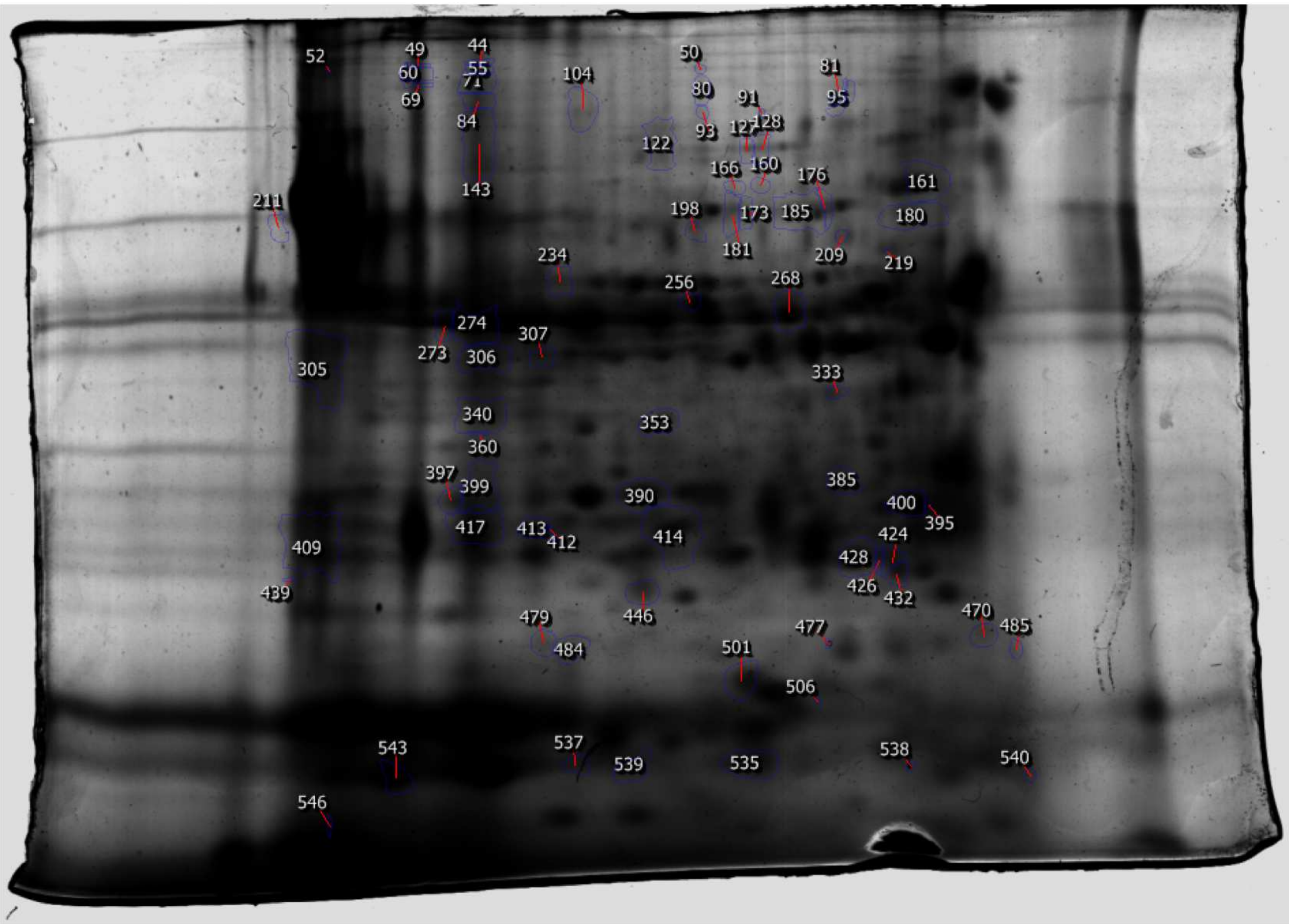

Experiment Design

Condition	Control	Ag50	Ag50+Amant
Replicates	2	2	2

Spots

#	Anova (p)	Fold	Tags	Notes	pI	MW	Protein Accession	Protein Description	Protein pI	Protein MW	Protein URL	Average Normalised Volumes		
												Control	Ag50	Ag50+Amant
80	2.675e-004	1.7										7092.072	6387.979	1.073e+004
546	2.808e-004	2.6										2306.223	1220.827	3191.972
477	3.207e-004	4.3										1501.317	349.420	478.164
428	0.001	1.9										6.725e+004	3.953e+004	7.391e+004
49	0.001	3.5										5787.316	3972.842	1632.780
60	0.001	2.4										2.393e+004	2.097e+004	9989.816
274	0.001	2.6										1.151e+005	1.061e+005	4.424e+004

Younes Comm FTSC Gills

Experiment: Younes Comm FTSC Gills

Report created: 22/12/2015 12:18:55

Reference image

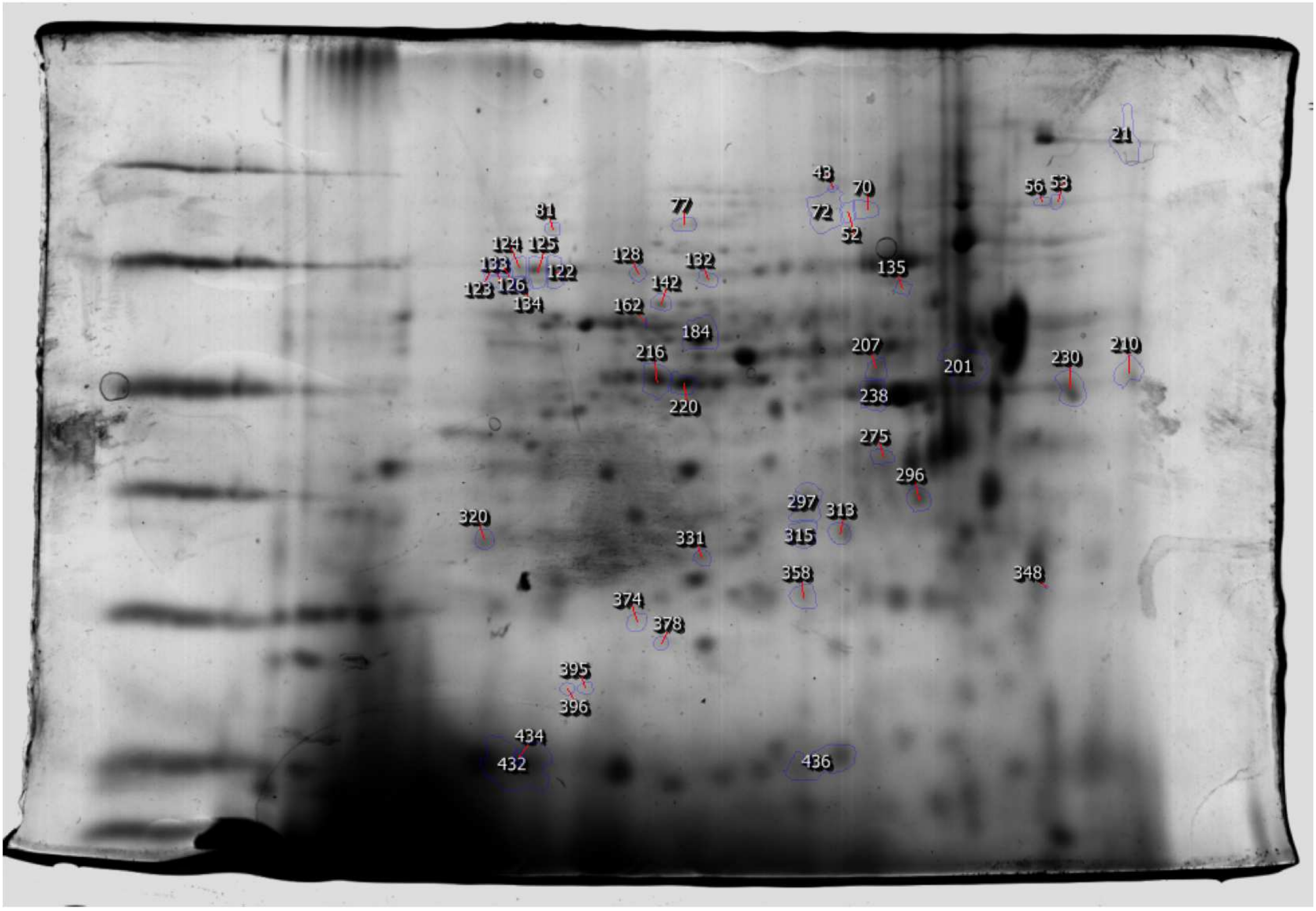

Experiment Design

Condition	Control	Ag50	Ag50+Amant
Replicates	2	2	2

Spots

#	Anova (p)	Fold	Tags	Notes	pI	MW	Protein Accession	Protein Description	Protein pI	Protein MW	Protein URL	Average Normalised Volumes		
												Control	Ag50	Ag50+Amant
320	3.531e-004	2.8										3519.223	5628.040	1983.307
238	0.002	1.9										7.442e+004	6.919e+004	3.998e+004
184	0.003	1.8										2.141e+004	3.934e+004	3.692e+004
220	0.005	4.8										5494.209	2.662e+004	1.948e+004
122	0.005	2.7										6910.671	1.891e+004	1.872e+004
162	0.006	1.5										572.182	882.553	612.822
132	0.006	2.2										2367.546	4254.008	5102.760
142	0.006	3.9										1757.939	6158.747	6884.593
[illegible]	[illegible]	[illegible]										[illegible]	[illegible]	[illegible]

Younes Comm IAF Gills

Experiment: Younes Comm IAF Gills

Report created: 22/12/2015 12:30:21

Reference image

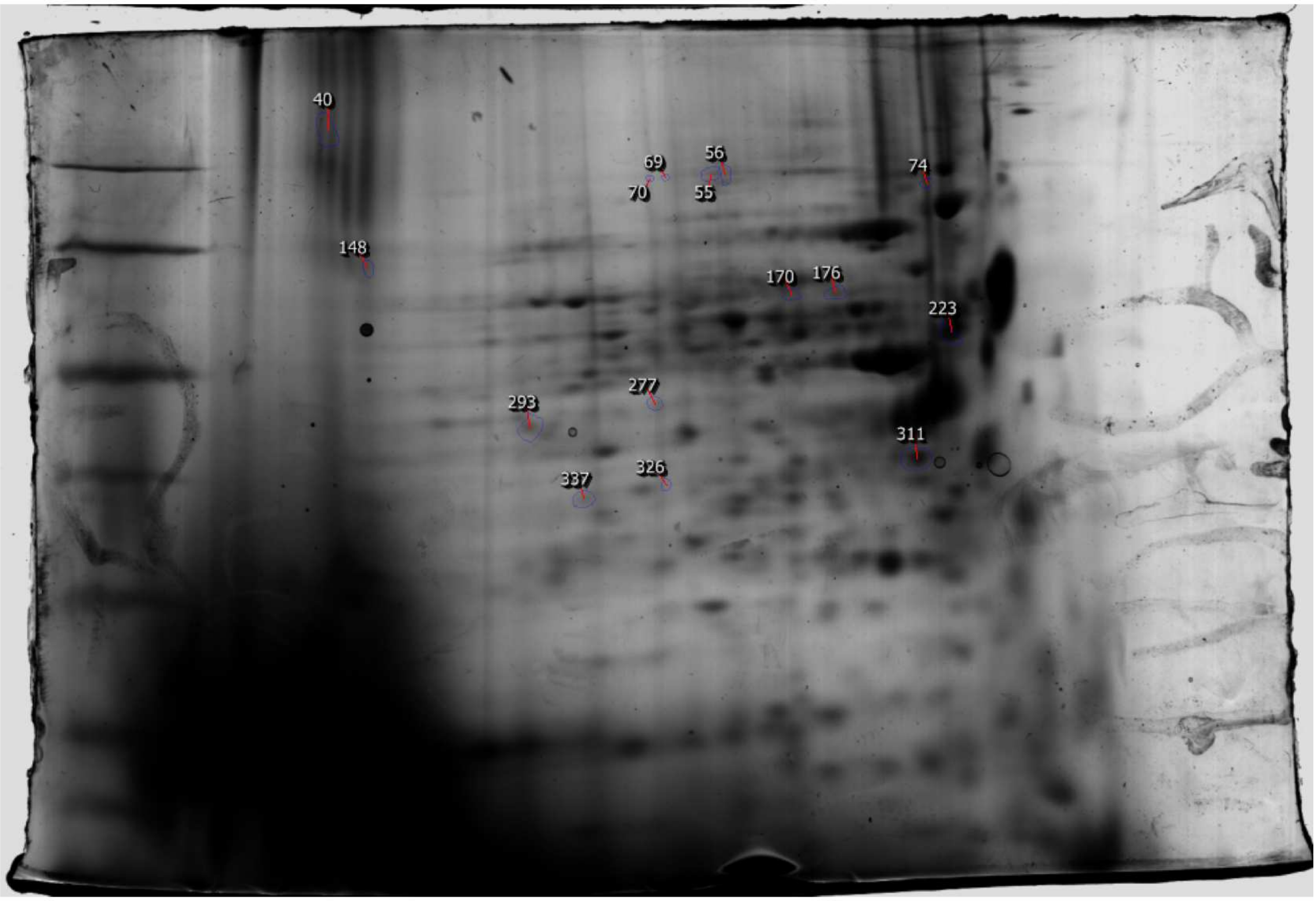

Experiment Design

Condition	Control	Ag50	Ag50+Amant
Replicates	2	2	2

Spots

#	Anova (p)	Fold	Tags	Notes	pI	MW	Protein Accession	Protein Description	Protein pI	Protein MW	Protein URL	Average Normalised Volumes		
												Control	Ag50	Ag50+Amant
55	8.787e-005	3.7										2796.984	5484.644	1480.067
70	0.004	4.3										324.923	697.084	163.253
148	0.005	2.8										2114.146	761.499	830.908
69	0.006	7.1										323.283	800.612	113.105
326	0.012	3.1										615.344	587.220	1829.790
223	0.018	2.4										1.323e+004	6297.628	5411.294
293	0.019	2.0										8735.312	1.767e+004	9116.154
277	0.023	2.1										2811.259	1368.490	2699.291
311	0.032	2.8										1.709e+004	1.842e+004	6584.466
74	0.033	2.1										2280.681	1235.220	1101.959

Younes FTSC Digestive gland

Experiment: Younes FTSC Digestive gland

Report created: 22/12/2015 10:48:10

Reference image

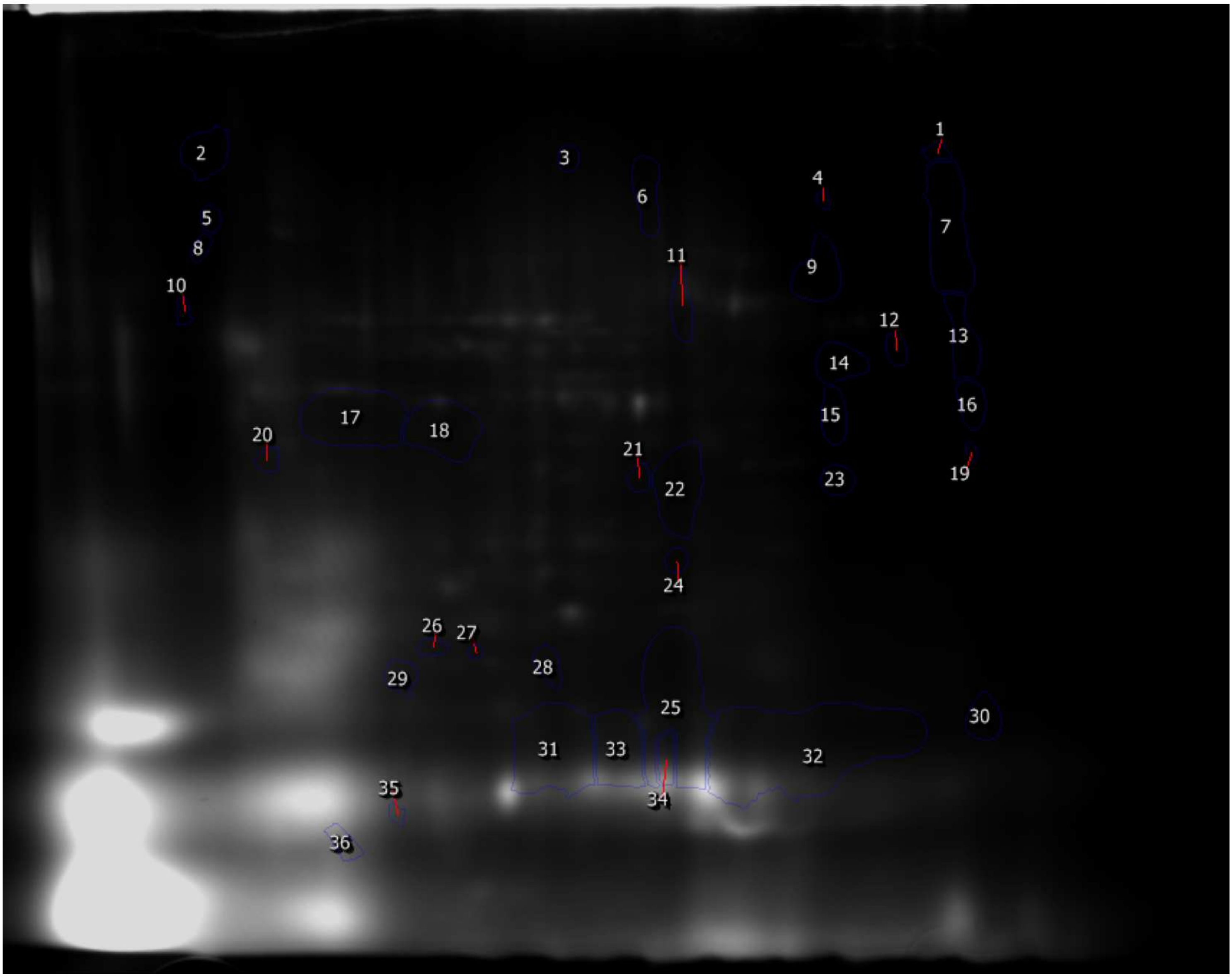

Experiment Design

Condition	Control	Ag50	Ag50+Amant
Replicates	2	2	2

Spots

#	Anova (p)	Fold	Tags	Notes	pI	MW	Protein Accession	Protein Description	Protein pI	Protein MW	Protein URL	Average Normalised Volumes		
												Control	Ag50	Ag50+Amant
11	0.137	2.1										4026.799	8614.313	7978.368
26	0.145	2.4										1873.272	1173.456	795.234

Younes FTSC Gills

Experiment: Younes FTSC Gills

Report created: 22/12/2015 11:18:45

Reference image

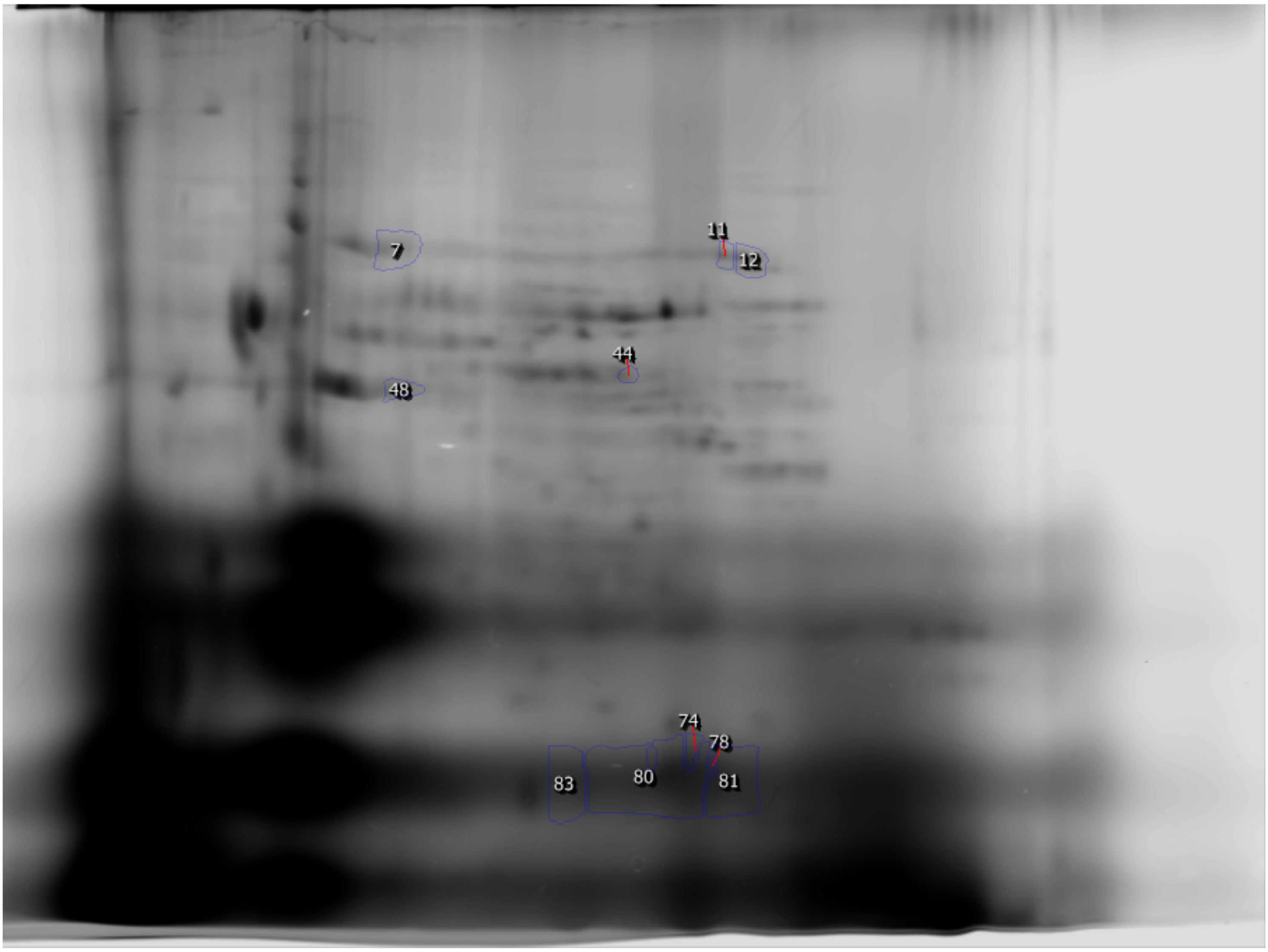

Experiment Design

Condition	Control	Ag50	Ag+Amant
Replicates	2	2	2

Spots

#	Anova (p)	Fold	Tags	Notes	pI	MW	Protein Accession	Protein Description	Protein pI	Protein MW	Protein URL	Average Normalised Volumes		
												Control	Ag50	Ag+Amant
80	0.002	2.7										1.183e+005	8.999e+004	4.365e+004
11	0.004	2.6										1174.056	1925.854	3016.544
7	0.005	3.0										6374.531	6939.084	1.898e+004
48	0.009	3.5										2200.404	3597.704	1020.882
[illegible]	[illegible]	[illegible]										[illegible]	[illegible]	[illegible]

Younes IAF Digestive gland

Experiment: Younes IAF Digestive gland

Report created: 22/12/2015 11:02:18

Reference image

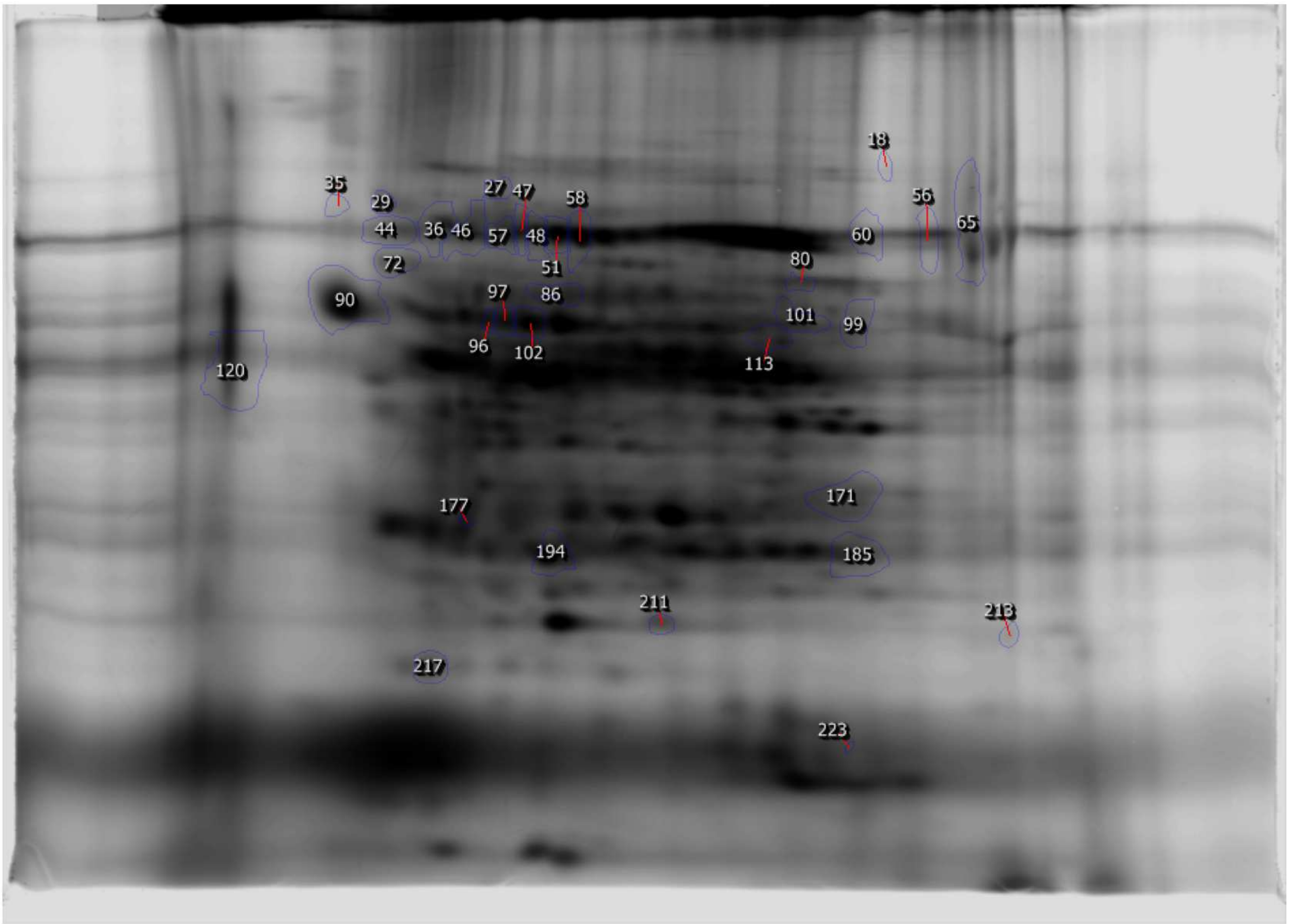

Experiment Design

Condition	Control	Ag50	Ag50+Amant
Replicates	2	2	2

Spots

#	Anova (p)	Fold	Tags	Notes	pI	MW	Protein Accession	Protein Description	Protein pI	Protein MW	Protein URL	Average Normalised Volumes		
												Control	Ag50	Ag50+Amant
211	1.118e-004	5.8										663.436	3852.552	1250.123
36	5.449e-004	1.8										1.021e+004	1.228e+004	1.827e+004
65	6.484e-004	3.2										6.127e+004	1.930e+004	3.642e+004
46	0.001	2.0										9322.541	1.673e+004	1.876e+004
35	0.001	4.2										4432.856	1122.669	1061.093
44	0.002	2.2										7272.546	1.542e+004	1.605e+004
[illegible]	[illegible]	[illegible]										[illegible]	[illegible]	[illegible]

Younes IAF Gills

Experiment: Younes IAF Gills

Report created: 22/12/2015 11:27:09

Reference image

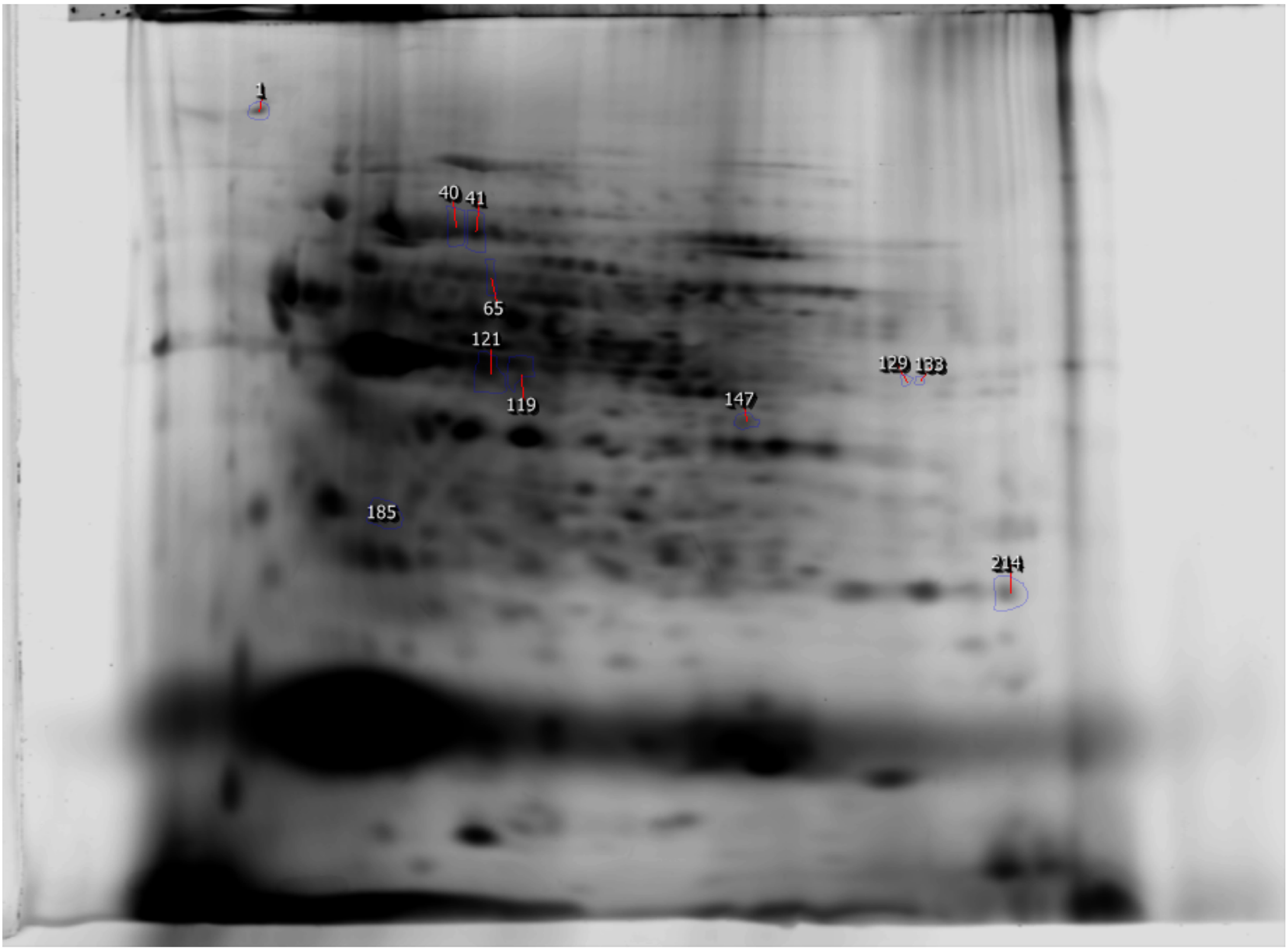

Experiment Design

Condition	Control	Ag50	Ag50+Amant
Replicates	2	2	2

Spots

#	Anova (p)	Fold	Tags	Notes	pI	MW	Protein Accession	Protein Description	Protein pI	Protein MW	Protein URL	Average Normalised Volumes		
												Control	Ag50	Ag50+Amant
119	0.003	1.9										7630.601	1.276e+004	6718.368
41	0.003	1.5										7842.361	6956.848	5147.865
1	0.004	6.2										1941.800	2607.275	420.348
129	0.005	5.3										146.132	78.960	415.796
147	0.018	2.1										1865.296	3094.422	1443.348
