# Supplementary material for: Redox proteomic insights into involvement of clathrin-mediated endocytosis in silver nanoparticles toxicity to Mytilus galloprovincialis
Source: PLoS One. 2018 Oct 29;13(10):e0205765. doi: 10.1371/journal.pone.0205765 (PMC6205585; doi:10.1371/journal.pone.0205765)
Supplement: S1 File — (DOCX) [file pone.0205765.s002.docx]

**Brief description of Supplementary documents S1 and S2:**

**S1: Output of the Progenesis same-spot software analyses of 2-dimentional gel electrophoresis featuring comparisons of gel images to the image of reference gel.** S1 contains comparisons of images from 2D gel separations of proteins labeled with fluorescent 5-iodoacetamido-fluorescein (IAF), 5-fluoresceinthiosemicarbazide (FTSC). Thereafter those same gels were stained with colloidal coomassie (Com). Such a procedure enabled selection of spots of interest (showing a 1.5-fold change between treatments as well as having a p < 0.05 in ANOVA), automatically generated by the software, and show features from the fluorescence images matched to the coomassie-stained features.

**S2: Output of the Progenesis same-spot software analyses of 2-dimentional gel electrophoresis: full details of features values showing significant changes (tags: ANOVA<0.05 and fold changes>=1.5).** S2 contains full detailed values of the comparisons between different treatments.

Note: all the supplemental materials are the output of the analysis from the same worksheet of the same software. The analysis performed using the Progenesis-Same-Spots software (Nonlinear Dynamics Limited, UK).
