# Supplementary material for: Redox proteomic insights into involvement of clathrin-mediated endocytosis in silver nanoparticles toxicity to Mytilus galloprovincialis
Source: PLoS One. 2018 Oct 29;13(10):e0205765. doi: 10.1371/journal.pone.0205765 (PMC6205585; doi:10.1371/journal.pone.0205765)

Comm FTSC Digestive gland

Experiment: Comm FTSC Digestive gland

Report created: 22/12/2015 12:07:14

Reference image

Experiment Design

Condition	Control	Ag50	Ag50+Amant
Replicates	2	2	2

Spots

#	Anova (p)	Fold	Tags	Notes	pI	MW	Protein Accession	Protein Description	Protein pI	Protein MW	Protein URL	Average Normalised Volumes		
												Control	Ag50	Ag50+Amant
266	0.001	3.8										2068.767	7877.707	2323.349
509	0.002	6.1										6909.977	1139.788	4166.555
162	0.003	2.2										2.437e+004	3.666e+004	5.295e+004
139	0.003	2.1										476.292	980.278	476.948
379	0.003	4.5										2519.910	8907.904	1999.094
486	0.008	5.0										8.841e+004	1.770e+004	6.289e+004
362	0.010	4.6										359.683	1649.693	507.505
186	0.010	5.9										4342.317	1544.049	731.466
405	0.011	2.9										1.746e+005	9.848e+004	6.121e+004
284	0.011	3.9										1.871e+004	3.468e+004	8957.324
375	0.014	5.2										608.995	3151.910	870.360
73	0.015	5.4										7.968e+004	1.478e+004	1.906e+004
56	0.017	2.8										1267.272	1450.878	521.998
294	0.019	3.8										587.425	1137.711	296.618
402	0.020	2.3										8267.880	1.644e+004	1.875e+004
422	0.023	1.8										8.084e+004	8.901e+004	1.479e+005
391	0.025	4.6										301.305	705.573	154.426
471	0.025	4.1										5217.337	1260.038	3109.684
566	0.026	1.9										2.019e+005	1.644e+005	3.204e+005
317	0.028	2.3										1.951e+005	8.923e+004	8.606e+004
423	0.028	1.7										1.204e+005	1.163e+005	7.191e+004
281	0.029	3.6										9.410e+004	7.411e+004	2.589e+004
155	0.031	5.5										7.298e+004	1.335e+004	4.465e+004
87	0.035	4.2										4389.618	1288.820	1043.005
116	0.041	7.0										260.528	1823.333	946.051
42	0.043	3.9										670.621	2146.837	556.712
134	0.045	9.8										7084.098	2375.810	2.336e+004
358	0.048	6.9										483.999	3328.716	656.628

Tags	
	Anova p-value ≤ 0.05
	Max fold change ≥ 1.5

Identifier 266

Position (904, 372)

Notes

- Anova p-value ≤ 0.05
- Max fold change ≥ 1.5

Control	Ag50	Ag50+Amant
Com Scan samples 37 FTSC	Com Scan samples 38 FTSC	Com Scan samples 65 FTSC

Identifier 509

Position (1266, 805)

Notes

- Anova p-value ≤ 0.05
- Max fold change ≥ 1.5

Control	Ag50	Ag50+Amant
Com Scan samples 37 FTSC	Com Scan samples 38 FTSC	Com Scan samples 65 FTSC

Identifier 162

Position (1100, 252)

Notes

- Anova p-value ≤ 0.05
- Max fold change ≥ 1.5

Control	Ag50	Ag50+Amant
Com Scan samples 37 FTSC	Com Scan samples 38 FTSC	Com Scan samples 65 FTSC

Identifier 139

Position (678, 232)

Notes

- Anova p-value ≤ 0.05
- Max fold change ≥ 1.5

Control	Ag50	Ag50+Amant
Com Scan samples 37 FTSC	Com Scan samples 38 FTSC	Com Scan samples 65 FTSC

Identifier 379

Position (1321, 541)

Notes

- Anova p-value ≤ 0.05
- Max fold change ≥ 1.5

Control	Ag50	Ag50+Amant
Com Scan samples 37 FTSC	Com Scan samples 38 FTSC	Com Scan samples 65 FTSC

Identifier 486

Position (414, 754)

Notes

- Anova p-value ≤ 0.05
- Max fold change ≥ 1.5

Control	Ag50	Ag50+Amant
Com Scan samples 37 FTSC	Com Scan samples 38 FTSC	Com Scan samples 65 FTSC

Identifier 362

Position (1248, 508)

Notes

- Anova p-value ≤ 0.05
- Max fold change ≥ 1.5

Control	Ag50	Ag50+Amant
Com Scan samples 37 FTSC	Com Scan samples 38 FTSC	Com Scan samples 65 FTSC

Identifier 186

Position (1320, 272)

Notes

- Anova p-value ≤ 0.05
- Max fold change ≥ 1.5

Control	Ag50	Ag50+Amant
Com Scan samples 37 FTSC	Com Scan samples 38 FTSC	Com Scan samples 65 FTSC

Identifier 405

Position (528, 596)

Notes

- Anova p-value ≤ 0.05
- Max fold change ≥ 1.5

Control	Ag50	Ag50+Amant
Com Scan samples 37 FTSC	Com Scan samples 38 FTSC	Com Scan samples 65 FTSC

Identifier 284

Position (541, 392)

Notes

- 🟥 Anova p-value ≤ 0.05
- 🟩 Max fold change ≥ 1.5

Control	Ag50	Ag50+Amant
Com Scan samples 37 FTSC	Com Scan samples 38 FTSC	Com Scan samples 65 FTSC

Identifier 375

Position (1242, 535)

Notes

- 🟥 Anova p-value ≤ 0.05
- 🟩 Max fold change ≥ 1.5

Control	Ag50	Ag50+Amant
Com Scan samples 37 FTSC	Com Scan samples 38 FTSC	Com Scan samples 65 FTSC

Identifier 73

Position (1161, 163)

Notes

- 🟥 Anova p-value ≤ 0.05
- 🟩 Max fold change ≥ 1.5

Control	Ag50	Ag50+Amant
Com Scan samples 37 FTSC	Com Scan samples 38 FTSC	Com Scan samples 65 FTSC

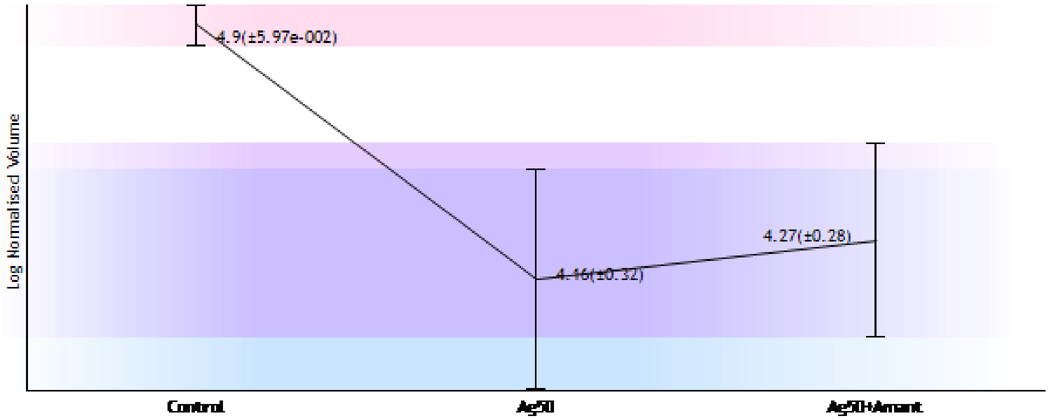

Identifier 56

Position (851, 154)

Notes

- 🟥 Anova p-value ≤ 0.05
- 🟩 Max fold change ≥ 1.5

Control	Ag50	Ag50+Amant
Com Scan samples 37 FTSC	Com Scan samples 38 FTSC	Com Scan samples 65 FTSC

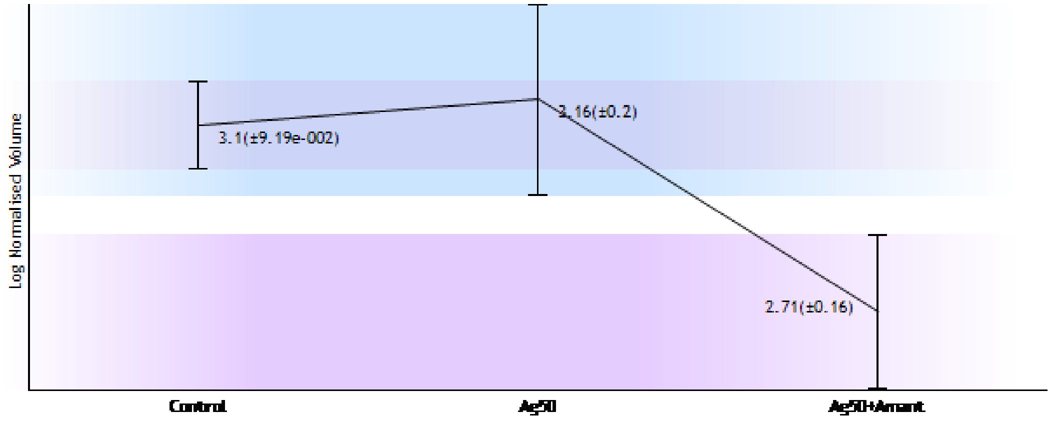

Identifier 294

Position (1256, 403)

Notes

- Anova p-value ≤ 0.05
- Max fold change ≥ 1.5

Control	Ag50	Ag50+Amant
Com Scan samples 37 FTSC	Com Scan samples 38 FTSC	Com Scan samples 65 FTSC

Identifier 402

Position (953, 590)

Notes

- Anova p-value ≤ 0.05
- Max fold change ≥ 1.5

Control	Ag50	Ag50+Amant
Com Scan samples 37 FTSC	Com Scan samples 38 FTSC	Com Scan samples 65 FTSC

Identifier 422

Position (862, 630)

Notes

- Anova p-value ≤ 0.05
- Max fold change ≥ 1.5

Control	Ag50	Ag50+Amant
Com Scan samples 37 FTSC	Com Scan samples 38 FTSC	Com Scan samples 65 FTSC

Identifier 391

Position (1333, 556)

Notes

- Anova p-value ≤ 0.05

- Max fold change ≥ 1.5

Control	Ag50	Ag50+Amant
Com Scan samples 37 FTSC	Com Scan samples 38 FTSC	Com Scan samples 65 FTSC

Identifier 471

Position (332, 723)

Notes

- Anova p-value ≤ 0.05
- Max fold change ≥ 1.5

Control	Ag50	Ag50+Amant
Com Scan samples 37 FTSC	Com Scan samples 38 FTSC	Com Scan samples 65 FTSC

Identifier 566

Position (451, 964)

Notes

- Anova p-value ≤ 0.05
- Max fold change ≥ 1.5

Control	Ag50	Ag50+Amant
Com Scan samples 37 FTSC	Com Scan samples 38 FTSC	Com Scan samples 65 FTSC

Identifier 317

Position (533, 437)

Notes

- Anova p-value ≤ 0.05
- Max fold change ≥ 1.5

Control	Ag50	Ag50+Amant
Com Scan samples 37 FTSC	Com Scan samples 38 FTSC	Com Scan samples 65 FTSC

Identifier 423

Position (528, 631)

Notes

- Anova p-value ≤ 0.05
- Max fold change ≥ 1.5

Control	Ag50	Ag50+Amant
Com Scan samples 37 FTSC	Com Scan samples 38 FTSC	Com Scan samples 65 FTSC

Identifier 281

Position (521, 389)

Notes

- Anova p-value ≤ 0.05
- Max fold change ≥ 1.5

Control	Ag50	Ag50+Amant
Com Scan samples 37 FTSC	Com Scan samples 38 FTSC	Com Scan samples 65 FTSC

Identifier 155

Position (422, 248)

Notes

- Anova p-value ≤ 0.05
- Max fold change ≥ 1.5

Control	Ag50	Ag50+Amant
Com Scan samples 37 FTSC	Com Scan samples 38 FTSC	Com Scan samples 65 FTSC

Identifier 87

Position (436, 175)

Notes

- Anova p-value ≤ 0.05
- Max fold change ≥ 1.5

Control	Ag50	Ag50+Amant
Com Scan samples 37 FTSC	Com Scan samples 38 FTSC	Com Scan samples 65 FTSC

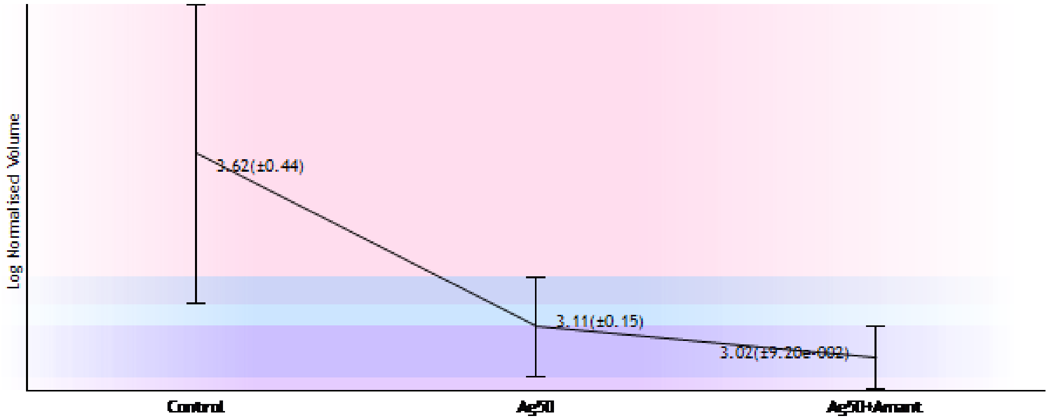

Identifier 116

Position (1215, 194)

Notes

- Anova p-value ≤ 0.05
- Max fold change ≥ 1.5

Control	Ag50	Ag50+Amant

Control	Ag50	Ag50+Amant
Com Scan samples 37 FTSC	Com Scan samples 38 FTSC	Com Scan samples 65 FTSC

Identifier 42

Position (1232, 148)

Notes

- Anova p-value ≤ 0.05
- Max fold change ≥ 1.5

Control	Ag50	Ag50+Amant
Com Scan samples 37 FTSC	Com Scan samples 38 FTSC	Com Scan samples 65 FTSC

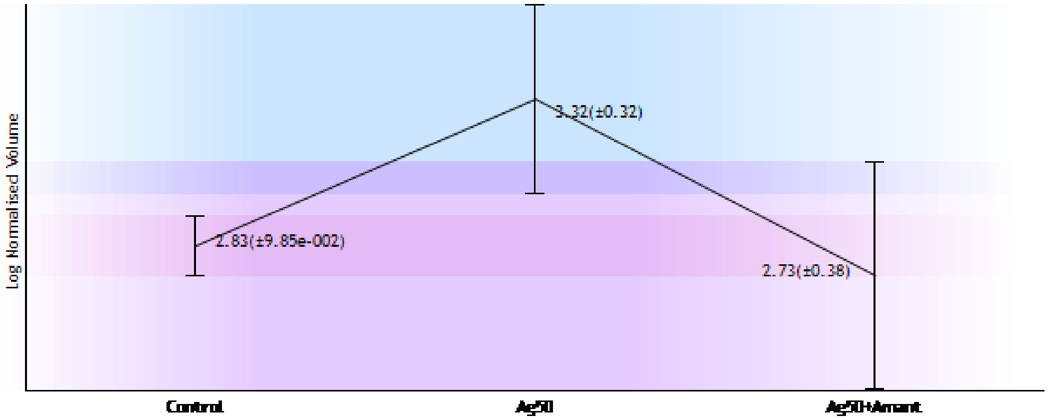

Identifier 134

Position (347, 227)

Notes

- Anova p-value ≤ 0.05
- Max fold change ≥ 1.5

Control	Ag50	Ag50+Amant
Com Scan samples 37 FTSC	Com Scan samples 38 FTSC	Com Scan samples 65 FTSC

Identifier 358

Position (1239, 502)

Notes

- Anova p-value ≤ 0.05
- Max fold change ≥ 1.5

Control	Ag50	Ag50+Amant
Com Scan samples 37 FTSC	Com Scan samples 38 FTSC	Com Scan samples 65 FTSC

Comm IAF Digestive gland

Experiment: Comm IAF Digestive gland

Report created: 22/12/2015 11:42:13

Reference image

Experiment Design

Condition	Control	Ag50	Ag50+Amant
Replicates	2	2	2

Spots

#	Anova (p)	Fold	Tags	Notes	pI	MW	Protein Accession	Protein Description	Protein pI	Protein MW	Protein URL	Average Normalised Volumes		
												Control	Ag50	Ag50+Amant
80	2.675e-004	1.7										7092.072	6387.979	1.073e+004
546	2.808e-004	2.6										2306.223	1220.827	3191.972
477	3.207e-004	4.3										1501.317	349.420	478.164
428	0.001	1.9										6.725e+004	3.953e+004	7.391e+004
49	0.001	3.5										5787.316	3972.842	1632.780
60	0.001	2.4										2.393e+004	2.097e+004	9989.816
274	0.001	2.6										1.151e+005	1.061e+005	4.424e+004
84	0.002	3.2										1.324e+004	2.717e+004	8428.964
50	0.003	1.6										1284.032	798.701	1287.643
306	0.003	2.0										4.113e+004	6.682e+004	3.327e+004
211	0.003	5.0										8070.129	1.243e+004	4.049e+004
307	0.003	2.3										5237.559	8367.065	1.216e+004
506	0.004	6.1										53.236	237.108	323.599
69	0.005	2.3										1.074e+004	8352.660	4704.056
95	0.005	2.2										6140.437	5421.613	1.210e+004
93	0.006	2.4										1851.653	1117.763	2640.737
44	0.007	3.5										3197.605	6993.640	2002.834
409	0.008	2.4										3.305e+005	1.399e+005	1.819e+005
333	0.008	1.9										4850.084	2531.736	2892.245
180	0.008	2.1										3.669e+004	5.812e+004	7.702e+004
501	0.009	1.6										5.060e+004	3.513e+004	5.690e+004
161	0.009	2.0										5.868e+004	1.051e+005	1.156e+005
256	0.009	1.6										1.531e+004	9638.893	1.528e+004
400	0.009	2.4										3.271e+004	1.874e+004	1.355e+004
538	0.010	4.2										1956.444	463.409	518.088
128	0.010	1.6										1.077e+004	1.679e+004	1.494e+004
426	0.011	2.1										5346.121	2604.582	4951.392
273	0.011	2.0										3.023e+004	3.378e+004	1.711e+004

#	Anova (p)	Fold	Tags	Notes	pI	MW	Protein Accession	Protein Description	Protein pI	Protein MW	Protein URL	Average Normalised Volumes		
												Control	Ag50	Ag50+Amant
104	0.011	1.7										1.851e+004	1.262e+004	2.173e+004
360	0.012	3.6										547.643	1382.143	384.509
537	0.013	1.7										1.038e+004	6318.583	6220.957
417	0.014	2.3										1.122e+005	6.702e+004	4.936e+004
395	0.015	2.2										671.726	856.757	385.236
81	0.016	2.5										2246.089	2507.184	5710.749
470	0.016	1.8										8015.427	1.058e+004	5867.306
397	0.018	2.3										1.556e+004	1.240e+004	6755.931
181	0.020	1.8										1.321e+004	2.410e+004	2.244e+004
143	0.020	3.0										6.297e+004	1.231e+005	4.145e+004
122	0.021	1.6										3.763e+004	5.886e+004	3.657e+004
424	0.021	2.1										1.719e+004	8223.744	1.454e+004
219	0.022	2.0										61.342	89.535	45.165
353	0.022	1.5										1.807e+004	2.384e+004	2.778e+004
390	0.024	1.7										3.675e+004	6.374e+004	4.545e+004
539	0.025	4.3										1.508e+004	7161.595	3.079e+004
209	0.025	2.3										1437.160	1033.568	2396.483
485	0.026	1.5										3583.188	2317.852	2352.497
185	0.027	2.3										5.339e+004	7.950e+004	1.230e+005
543	0.028	2.3										3.446e+004	1.487e+004	1.757e+004
127	0.028	1.6										7974.764	1.256e+004	1.065e+004
413	0.028	1.7										1.174e+004	1.339e+004	2.041e+004
479	0.029	1.6										1.080e+004	1.128e+004	1.717e+004
412	0.029	3.0										25.612	37.982	76.466
432	0.031	1.7										1.314e+004	7517.869	1.129e+004
439	0.032	5.8										2443.475	419.897	480.290
52	0.032	7.1										91.561	654.507	186.242
166	0.032	1.8										3476.475	6358.647	4221.059
340	0.032	1.7										7.817e+004	7.036e+004	4.537e+004
198	0.033	1.8										4891.708	8603.288	8993.776
176	0.035	2.0										8641.906	1.718e+004	1.467e+004
446	0.036	1.5										1.882e+004	2.134e+004	1.384e+004
234	0.039	1.8										1.712e+004	3.073e+004	3.141e+004
160	0.040	2.0										3196.734	6382.575	4576.113
535	0.041	2.2										4.134e+004	2.886e+004	6.277e+004
484	0.041	1.5										1.119e+004	1.362e+004	1.680e+004
305	0.042	2.1										3.420e+005	1.610e+005	2.563e+005
385	0.043	1.7										1.072e+004	1.771e+004	1.097e+004
540	0.043	2.2										1788.827	811.115	1224.076
91	0.044	2.8										732.103	359.157	264.742
399	0.044	2.0										3.706e+004	5.487e+004	2.752e+004
71	0.044	2.9										1.729e+004	3.842e+004	1.342e+004
173	0.045	1.6										7553.062	1.125e+004	1.196e+004
268	0.047	1.6										4.825e+004	3.912e+004	6.313e+004

#	Anova (p)	Fold	Tags	Notes	pI	MW	Protein Accession	Protein Description	Protein pI	Protein MW	Protein URL	Average Normalised Volumes		
												Control	Ag50	Ag50+Amant
414	0.049	1.6	🟥🟦									1.317e+005	1.612e+005	2.053e+005
55	0.050	3.6	🟥🟦									3977.371	9849.690	2769.017

Tags	
🟥	Anova p-value ≤ 0.05
🟦	Max fold change ≥ 1.5

Identifier 80

Position (799, 112)

Notes

- 🟥 Anova p-value ≤ 0.05
- 🟦 Max fold change ≥ 1.5

Control	Ag50	Ag50+Amant
Com Scan samples 37 IAF	Com Scan samples 38 IAF	Com Scan samples 65 IAF

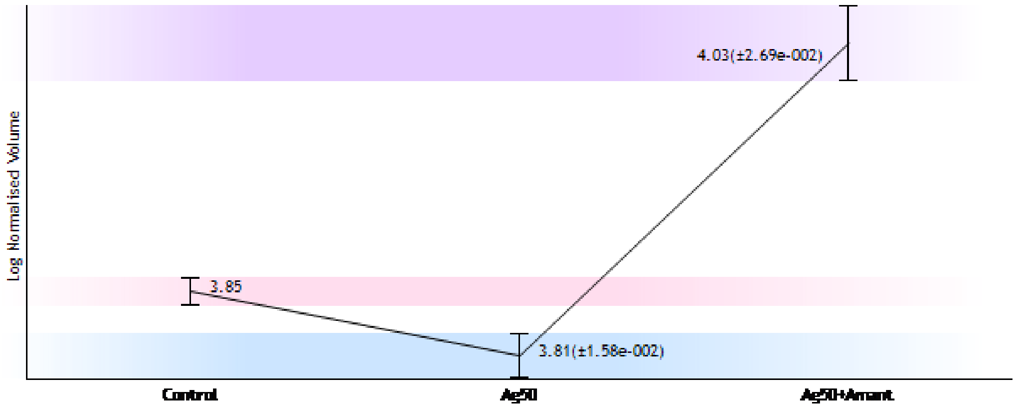

Identifier 546

Position (372, 925)

Notes

- 🟥 Anova p-value ≤ 0.05
- 🟦 Max fold change ≥ 1.5

Control	Ag50	Ag50+Amant
Com Scan samples 37 IAF	Com Scan samples 38 IAF	Com Scan samples 65 IAF

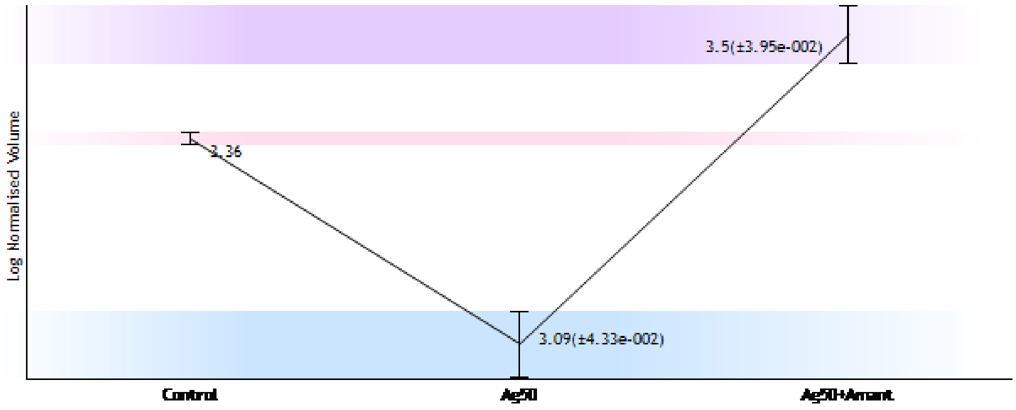

Identifier 477

Position (946, 728)

Notes

- Anova p-value ≤ 0.05
- Max fold change ≥ 1.5

Control	Ag50	Ag50+Amant
Com Scan samples 37 IAF	Com Scan samples 38 IAF	Com Scan samples 65 IAF

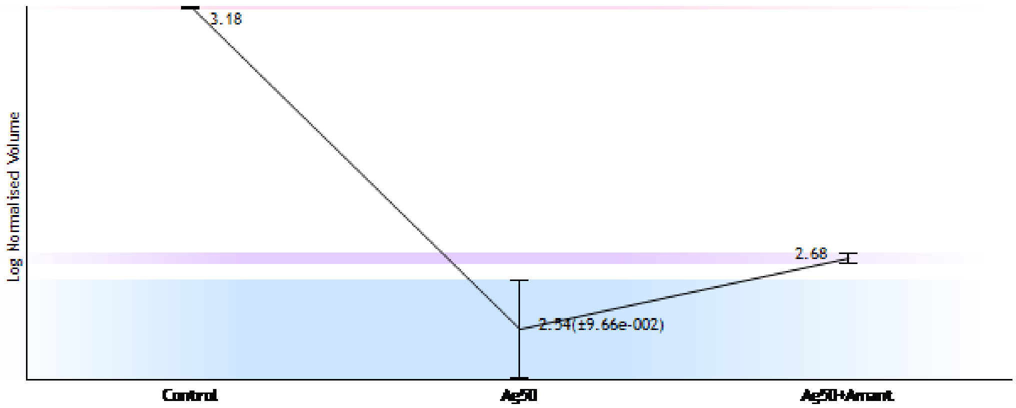

Identifier 428

Position (973, 628)

Notes

- Anova p-value ≤ 0.05
- Max fold change ≥ 1.5

Control	Ag50	Ag50+Amant
Com Scan samples 37 IAF	Com Scan samples 38 IAF	Com Scan samples 65 IAF

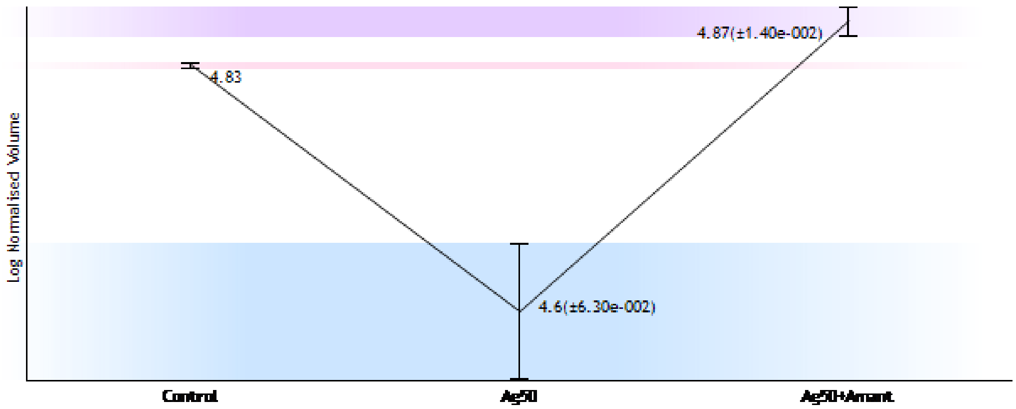

Identifier 49

Position (473, 68)

Notes

- Anova p-value ≤ 0.05
- Max fold change ≥ 1.5

Control	Ag50	Ag50+Amant

Control	Ag50	Ag50+Amant
Com Scan samples 37 IAF	Com Scan samples 38 IAF	Com Scan samples 65 IAF

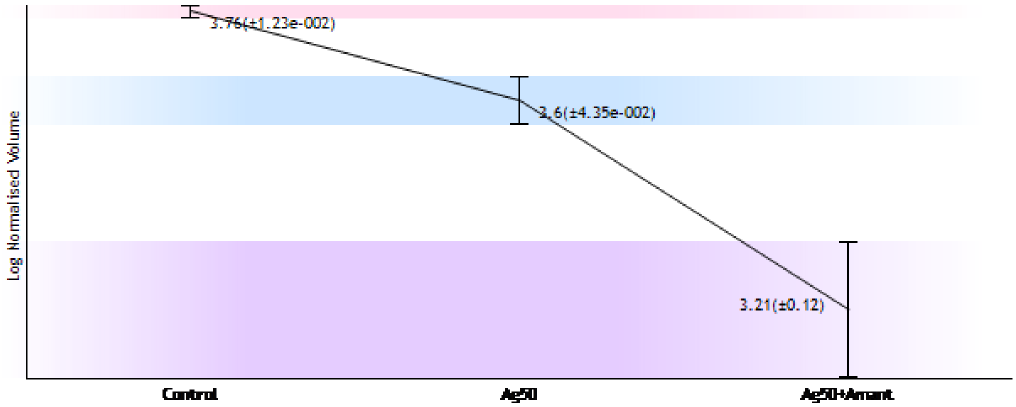

Identifier 60

Position (474, 79)

Notes

- Anova p-value ≤ 0.05
- Max fold change ≥ 1.5

Control	Ag50	Ag50+Amant
Com Scan samples 37 IAF	Com Scan samples 38 IAF	Com Scan samples 65 IAF

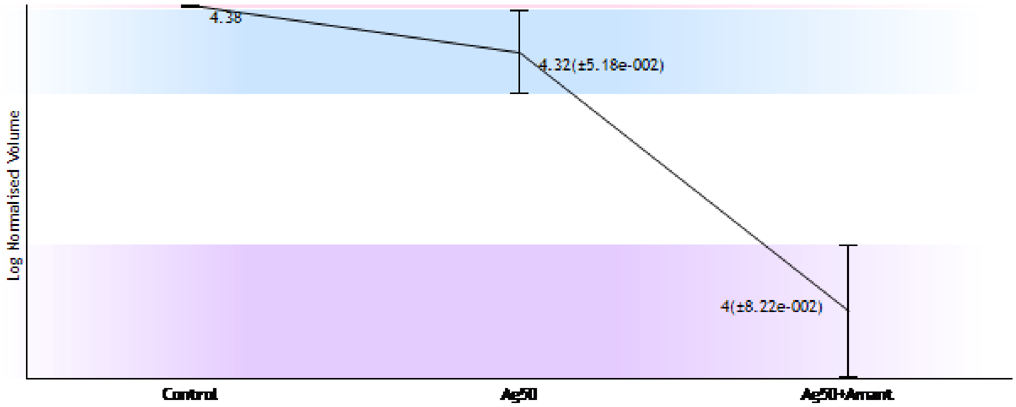

Identifier 274

Position (541, 359)

Notes

- Anova p-value ≤ 0.05
- Max fold change ≥ 1.5

Control	Ag50	Ag50+Amant
Com Scan samples 37 IAF	Com Scan samples 38 IAF	Com Scan samples 65 IAF

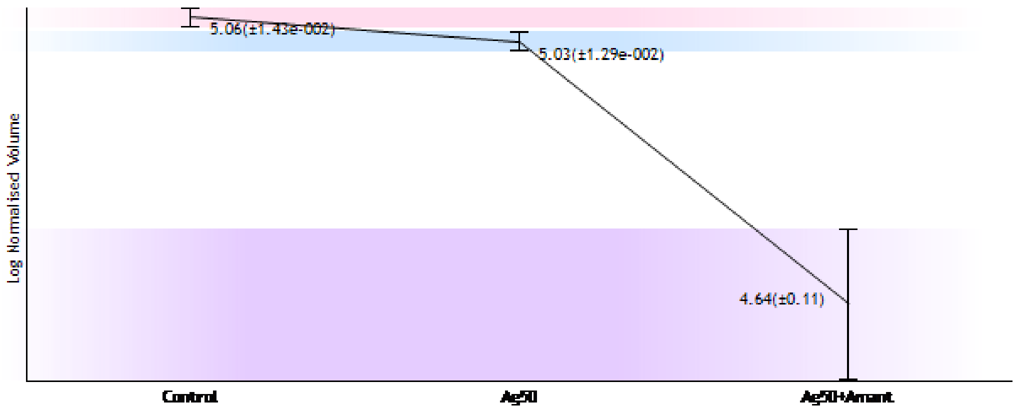

Identifier 84

Position (541, 114)

Notes

- Anova p-value ≤ 0.05
- Max fold change ≥ 1.5

Control	Ag50	Ag50+Amant
Com Scan samples 37 IAF	Com Scan samples 38 IAF	Com Scan samples 65 IAF

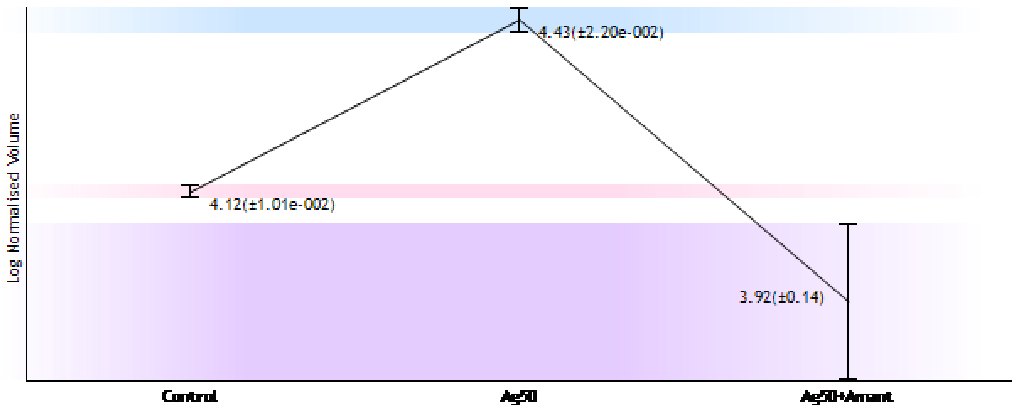

Identifier 50

Position (796, 71)

Notes

- Anova p-value ≤ 0.05
- Max fold change ≥ 1.5

Control	Ag50	Ag50+Amant
Com Scan samples 37 IAF	Com Scan samples 38 IAF	Com Scan samples 65 IAF

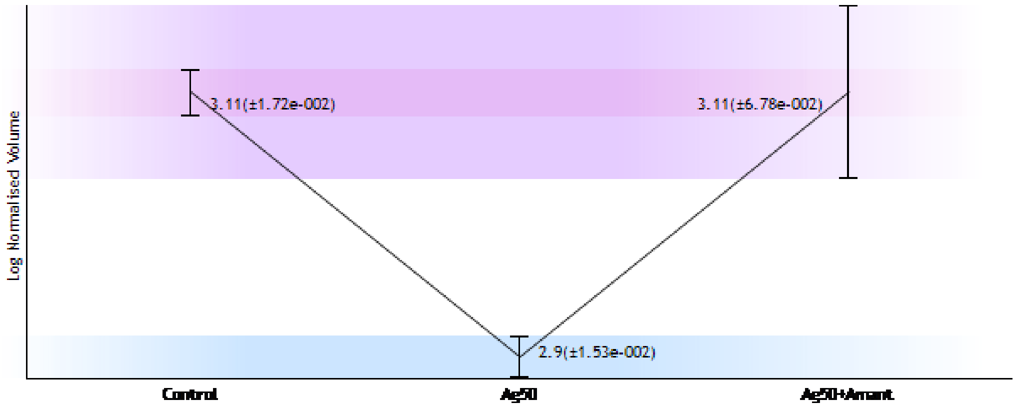

Identifier 306

Position (549, 398)

Notes

- Anova p-value ≤ 0.05
- Max fold change ≥ 1.5

Control	Ag50	Ag50+Amant
Com Scan samples 37 IAF	Com Scan samples 38 IAF	Com Scan samples 65 IAF

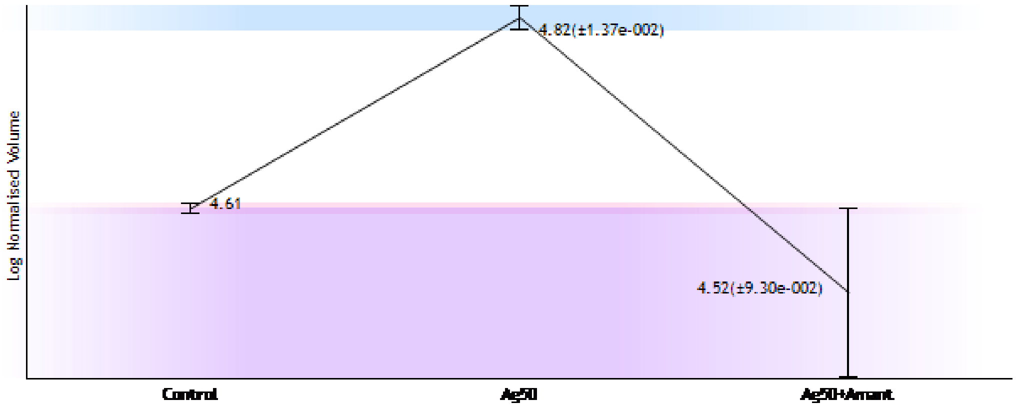

Identifier 211

Position (315, 266)

Notes

- Anova p-value ≤ 0.05
- Max fold change ≥ 1.5

Control	Ag50	Ag50+Amant
Com Scan samples 37 IAF	Com Scan samples 38 IAF	Com Scan samples 65 IAF

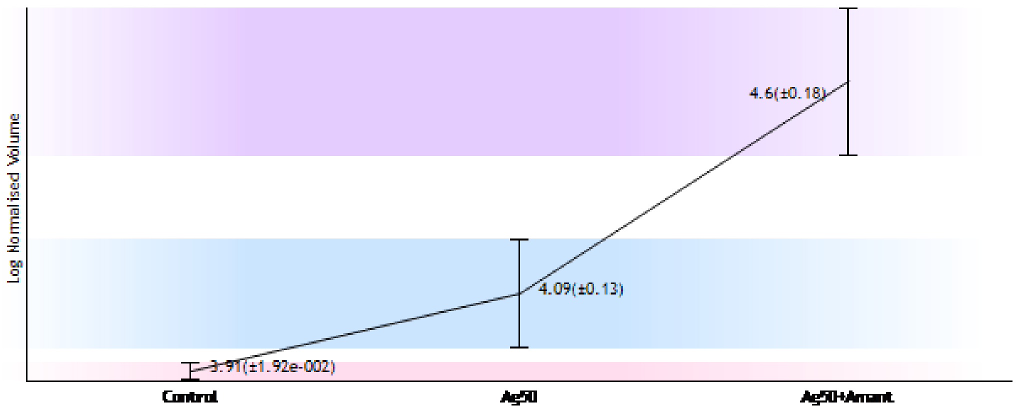

Identifier 307

Position (614, 398)

Notes

- 🟥 Anova p-value ≤ 0.05
- 🟦 Max fold change ≥ 1.5

Control	Ag50	Ag50+Amant
Com Scan samples 37 IAF	Com Scan samples 38 IAF	Com Scan samples 65 IAF

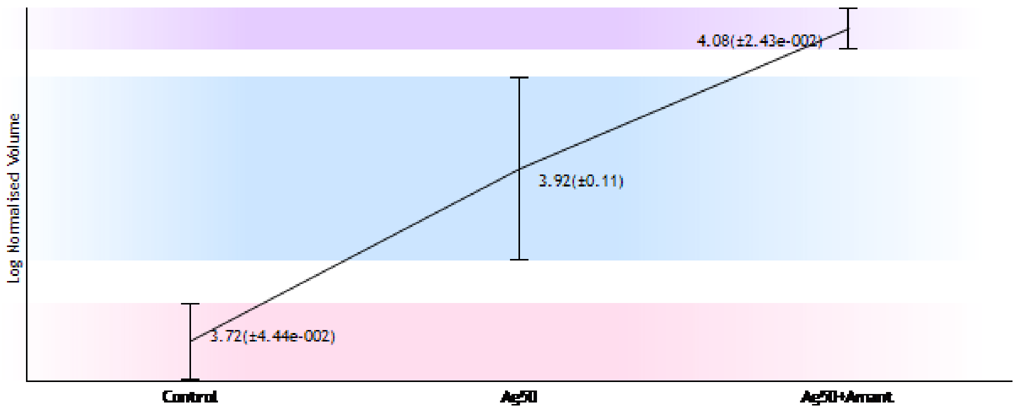

Identifier 506

Position (930, 792)

Notes

- 🟥 Anova p-value ≤ 0.05
- 🟦 Max fold change ≥ 1.5

Control	Ag50	Ag50+Amant
Com Scan samples 37 IAF	Com Scan samples 38 IAF	Com Scan samples 65 IAF

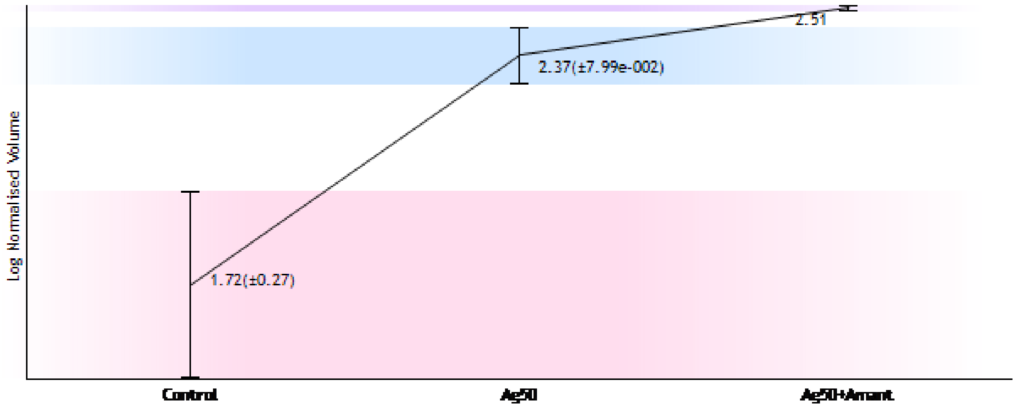

Identifier 69

Position (473, 93)

Notes

- Anova p-value ≤ 0.05
- Max fold change ≥ 1.5

Control	Ag50	Ag50+Amant
Com Scan samples 37 IAF	Com Scan samples 38 IAF	Com Scan samples 65 IAF

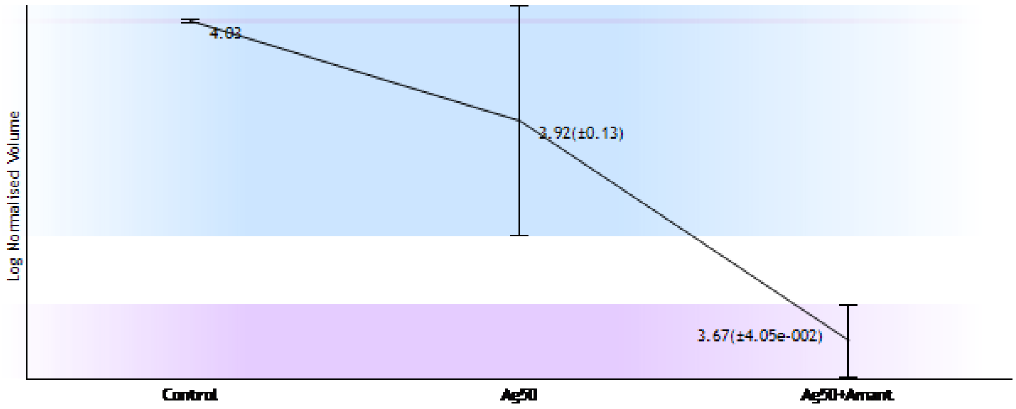

Identifier 95

Position (957, 126)

Notes

- Anova p-value ≤ 0.05
- Max fold change ≥ 1.5

Control	Ag50	Ag50+Amant
Com Scan samples 37 IAF	Com Scan samples 38 IAF	Com Scan samples 65 IAF

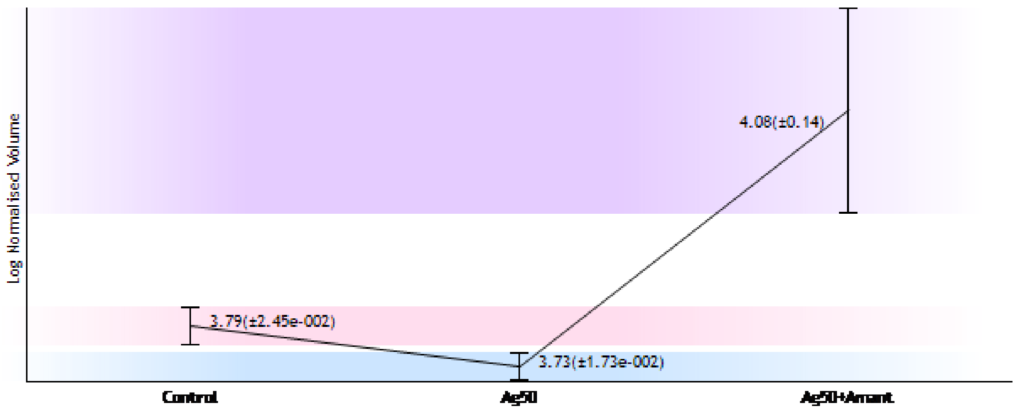

Identifier 93

Position (800, 122)

Notes

- Anova p-value ≤ 0.05
- Max fold change ≥ 1.5

Control	Ag50	Ag50+Amant
Com Scan samples 37 IAF	Com Scan samples 38 IAF	Com Scan samples 65 IAF

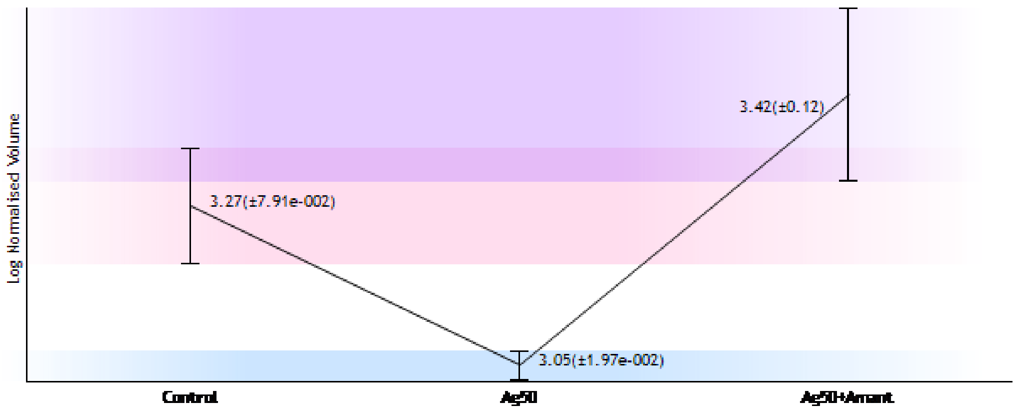

Identifier 44

Position (558, 60)

Notes

- Anova p-value ≤ 0.05
- Max fold change ≥ 1.5

Control	Ag50	Ag50+Amant
Com Scan samples 37 IAF	Com Scan samples 38 IAF	Com Scan samples 65 IAF

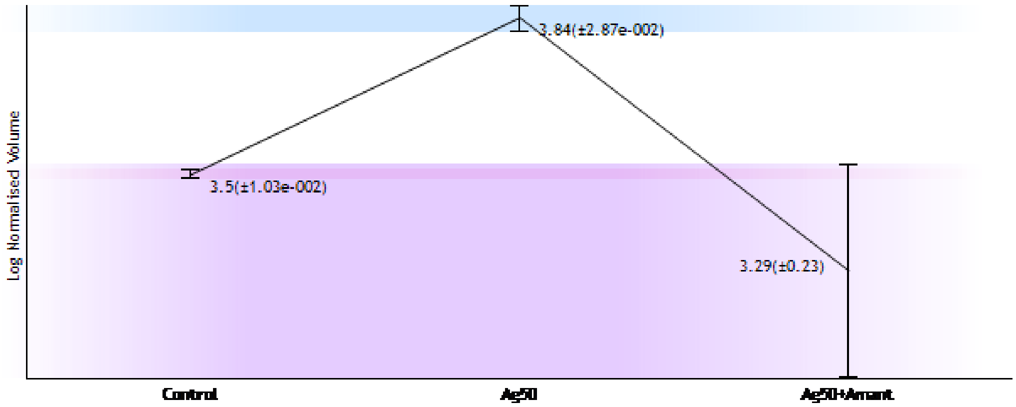

Identifier 409

Position (366, 593)

Notes

- Anova p-value ≤ 0.05
- Max fold change ≥ 1.5

Control	Ag50	Ag50+Amant
Com Scan samples 37 IAF	Com Scan samples 38 IAF	Com Scan samples 65 IAF

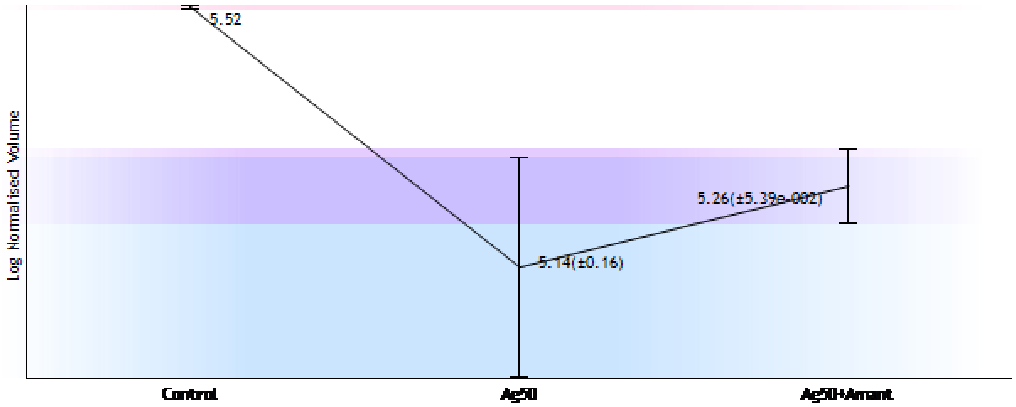

Identifier 333

Position (948, 440)

Notes

- Anova p-value ≤ 0.05
- Max fold change ≥ 1.5

Control	Ag50	Ag50+Amant
Com Scan samples 37 IAF	Com Scan samples 38 IAF	Com Scan samples 65 IAF

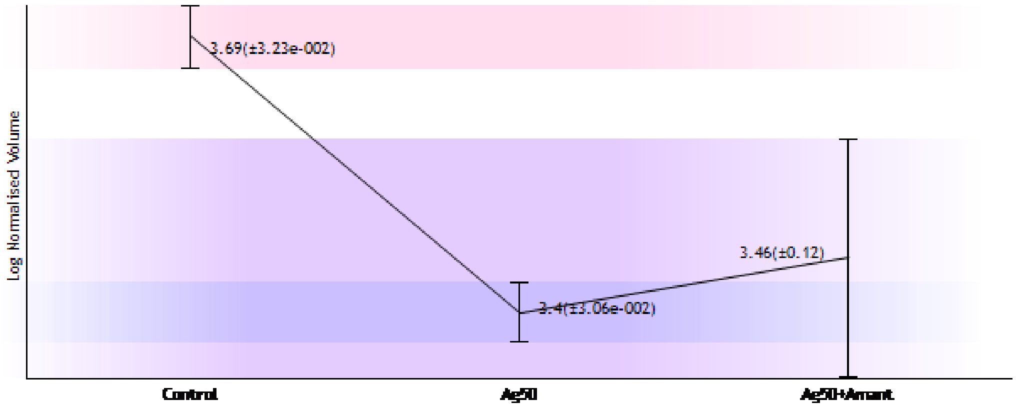

Identifier 180

Position (1028, 234)

Notes

- Anova p-value ≤ 0.05
- Max fold change ≥ 1.5

Control	Ag50	Ag50+Amant
Com Scan samples 37 IAF	Com Scan samples 38 IAF	Com Scan samples 65 IAF

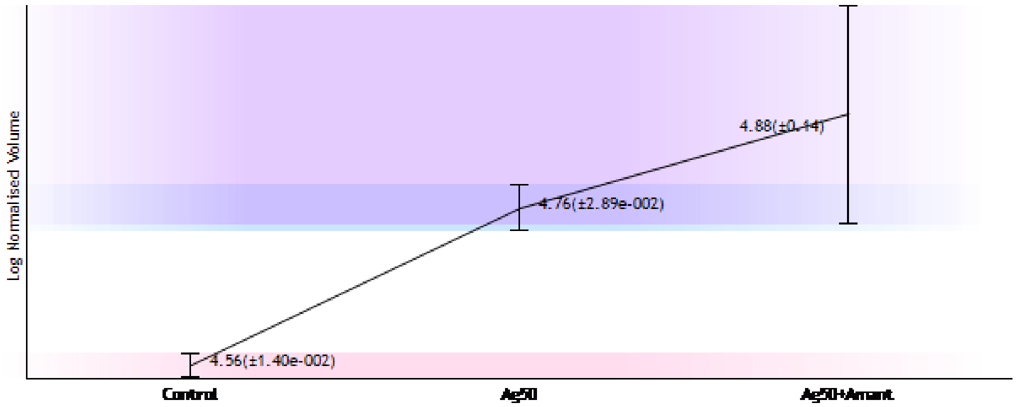

Identifier 501

Position (859, 774)

Notes

- Anova p-value ≤ 0.05
- Max fold change ≥ 1.5

Control	Ag50	Ag50+Amant
Com Scan samples 37 IAF	Com Scan samples 38 IAF	Com Scan samples 65 IAF

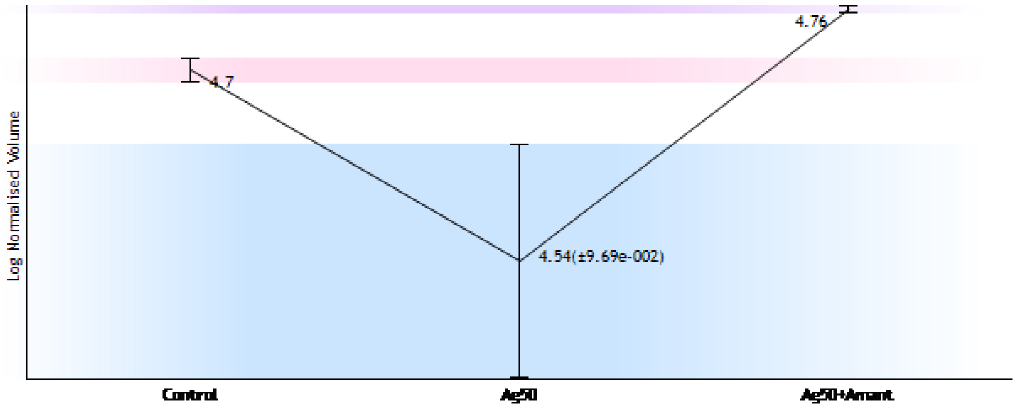

Identifier 161

Position (1045, 206)

Notes

- Anova p-value ≤ 0.05
- Max fold change ≥ 1.5

Control	Ag50	Ag50+Amant
Com Scan samples 37 IAF	Com Scan samples 38 IAF	Com Scan samples 65 IAF

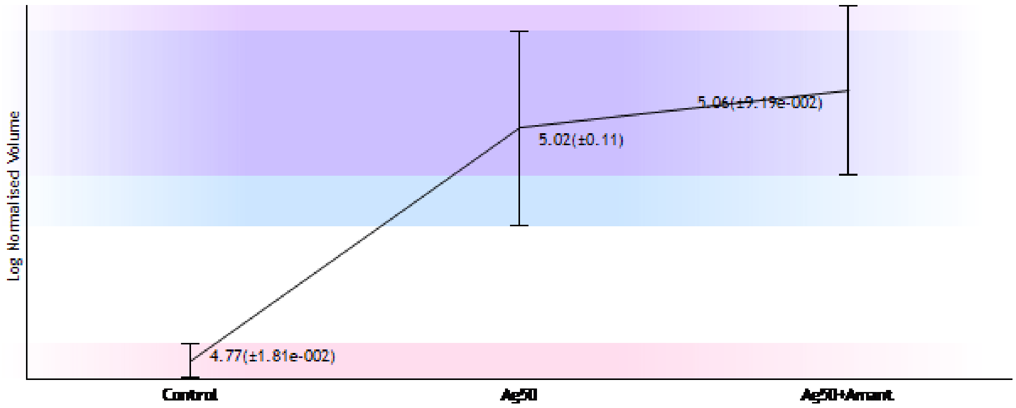

Identifier 256

Position (797, 344)

Notes

- Anova p-value ≤ 0.05
- Max fold change ≥ 1.5

Control	Ag50	Ag50+Amant
Com Scan samples 37 IAF	Com Scan samples 38 IAF	Com Scan samples 65 IAF

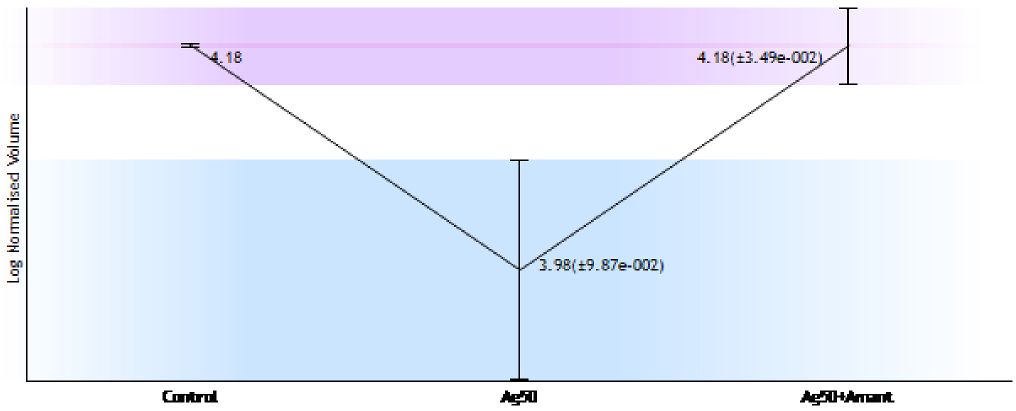

Identifier 400

Position (1042, 569)

Notes

- Anova p-value ≤ 0.05
- Max fold change ≥ 1.5

Control	Ag50	Ag50+Amant
Com Scan samples 37 IAF	Com Scan samples 38 IAF	Com Scan samples 65 IAF

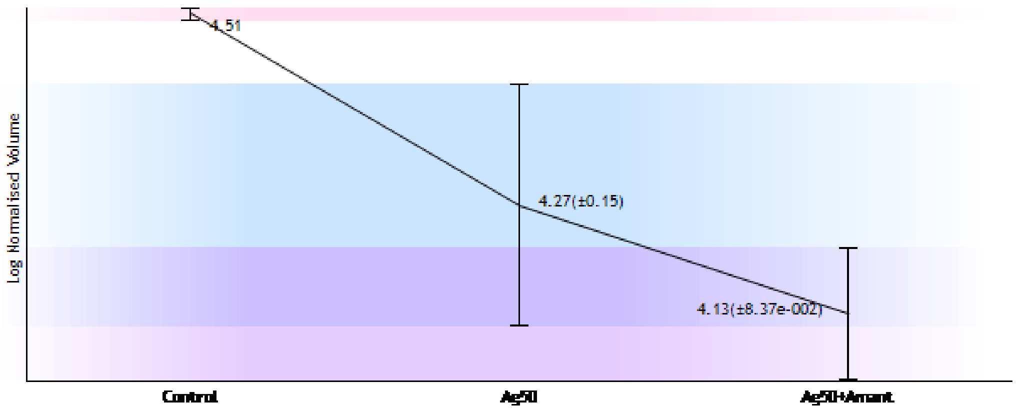

Identifier 538

Position (1035, 869)

Notes

- Anova p-value ≤ 0.05
- Max fold change ≥ 1.5

Control	Ag50	Ag50+Amant
Com Scan samples 37 IAF	Com Scan samples 38 IAF	Com Scan samples 65 IAF

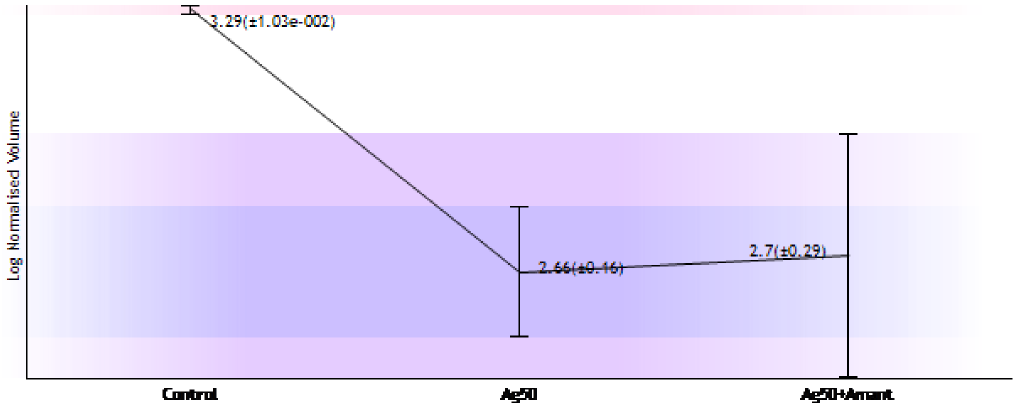

Identifier 128

Position (866, 163)

Notes

- Anova p-value ≤ 0.05
- Max fold change ≥ 1.5

Control	Ag50	Ag50+Amant
Com Scan samples 37 IAF	Com Scan samples 38 IAF	Com Scan samples 65 IAF

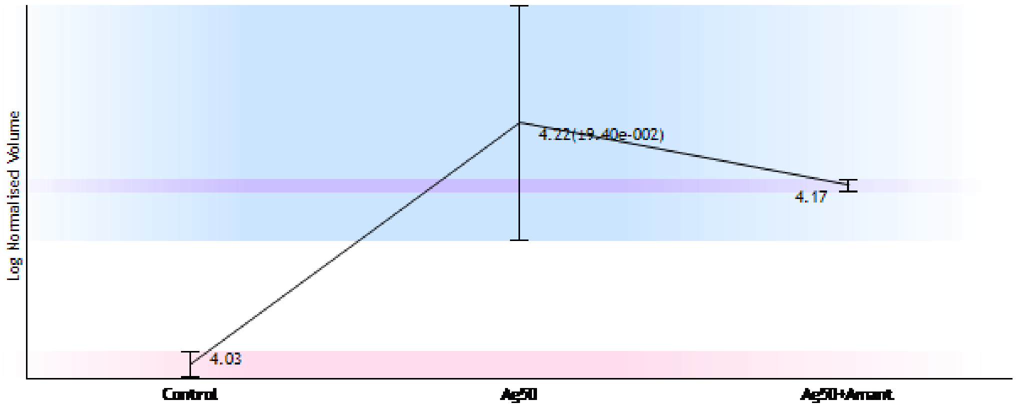

Identifier 426

Position (1004, 625)

Notes

- Anova p-value ≤ 0.05
- Max fold change ≥ 1.5

Control	Ag50	Ag50+Amant
Com Scan samples 37 IAF	Com Scan samples 38 IAF	Com Scan samples 65 IAF

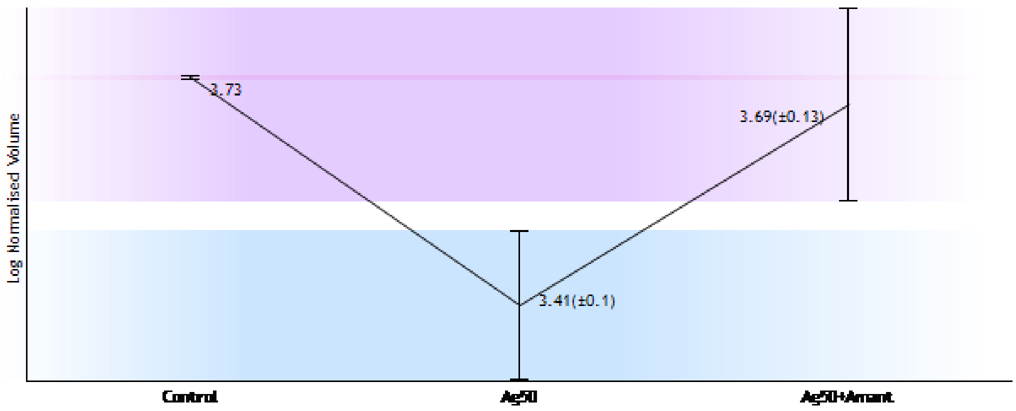

Identifier 273

Position (510, 359)

Notes

- Anova p-value ≤ 0.05
- Max fold change ≥ 1.5

Control	Ag50	Ag50+Amant
Com Scan samples 37 IAF	Com Scan samples 38 IAF	Com Scan samples 65 IAF

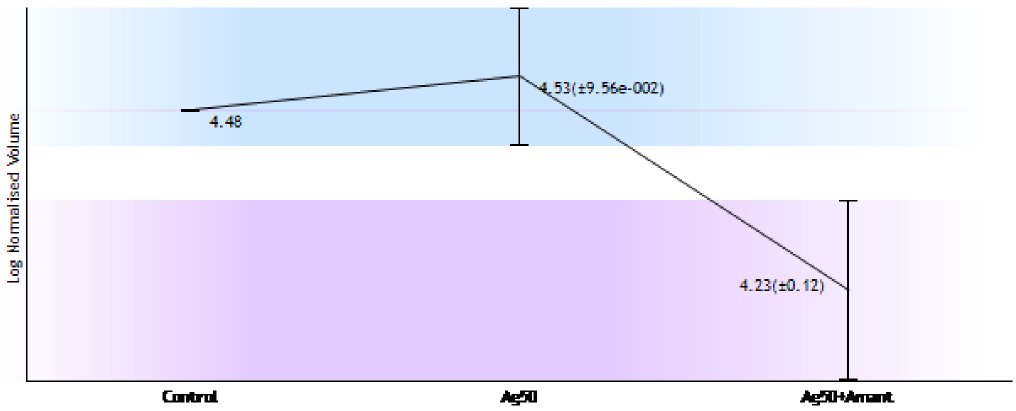

Identifier 104

Position (660, 137)

Notes

- Anova p-value ≤ 0.05
- Max fold change ≥ 1.5

Control	Ag50	Ag50+Amant
Com Scan samples 37 IAF	Com Scan samples 38 IAF	Com Scan samples 65 IAF

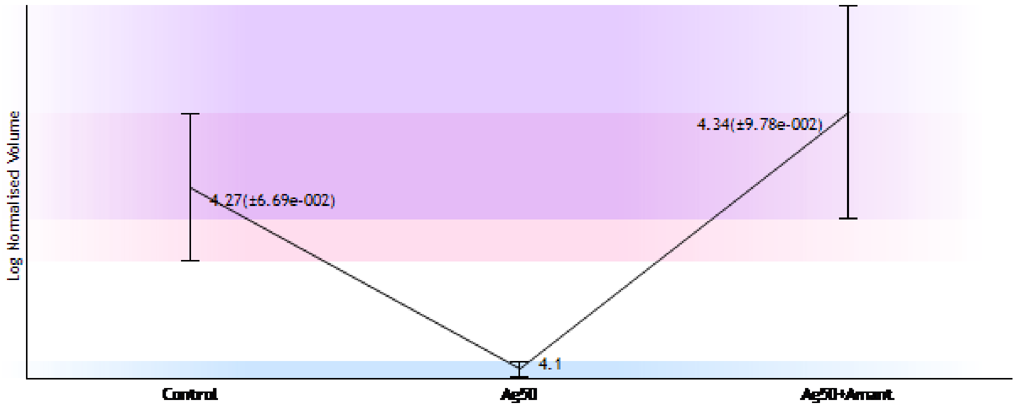

Identifier 360

Position (545, 489)

Notes

- Anova p-value ≤ 0.05
- Max fold change ≥ 1.5

Control	Ag50	Ag50+Amant
Com Scan samples 37 IAF	Com Scan samples 38 IAF	Com Scan samples 65 IAF

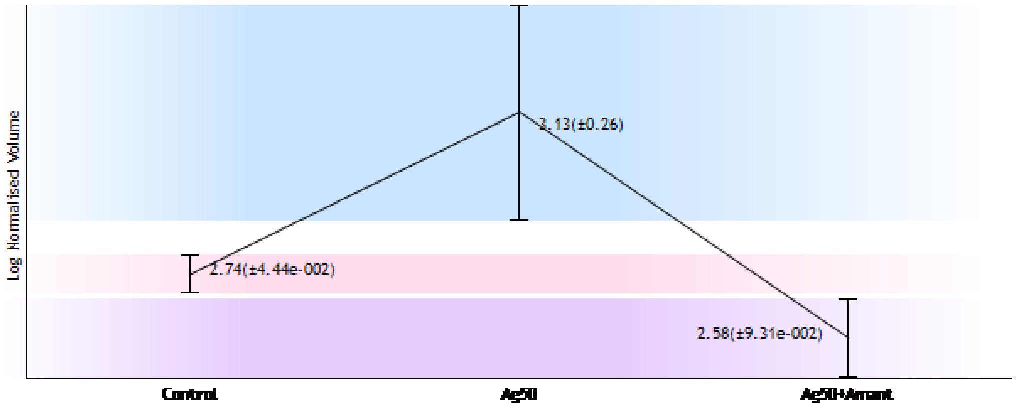

Identifier 537

Position (665, 864)

Notes

- Anova p-value ≤ 0.05
- Max fold change ≥ 1.5

Control	Ag50	Ag50+Amant
Com Scan samples 37 IAF	Com Scan samples 38 IAF	Com Scan samples 65 IAF

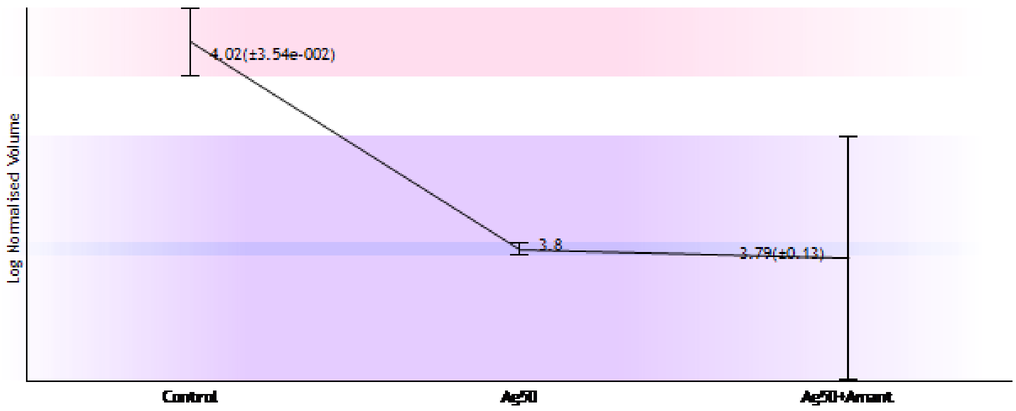

Identifier 417

Position (541, 605)

Notes

- Anova p-value ≤ 0.05
- Max fold change ≥ 1.5

Control	Ag50	Ag50+Amant
Com Scan samples 37 IAF	Com Scan samples 38 IAF	Com Scan samples 65 IAF

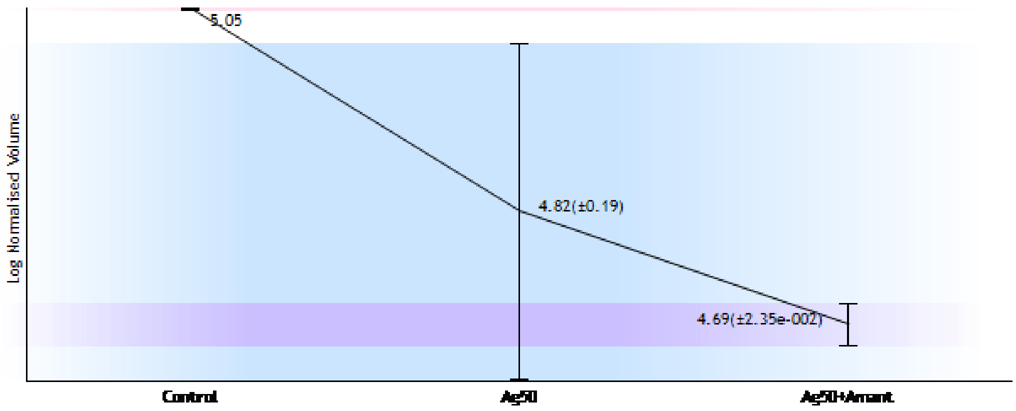

Identifier 395

Position (1058, 562)

Notes

- Anova p-value ≤ 0.05
- Max fold change ≥ 1.5

Control	Ag50	Ag50+Amant
Com Scan samples 37 IAF	Com Scan samples 38 IAF	Com Scan samples 65 IAF

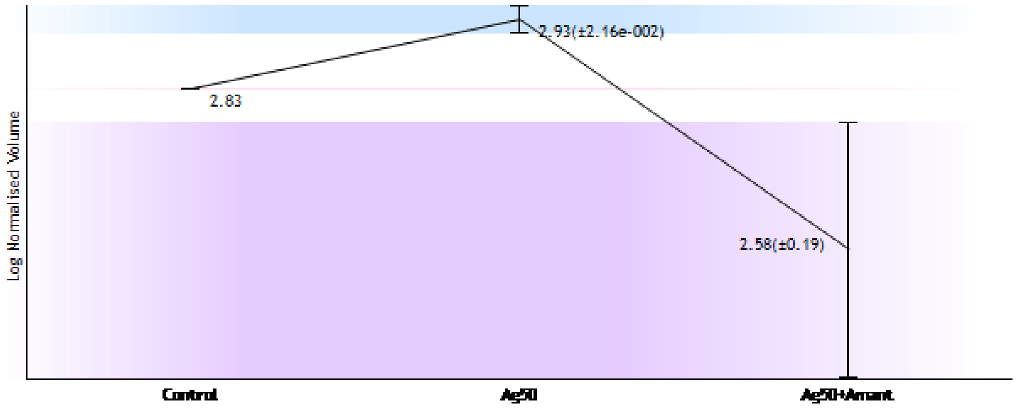

Identifier 81

Position (953, 112)

Notes

- Anova p-value ≤ 0.05
- Max fold change ≥ 1.5

Control	Ag50	Ag50+Amant
Com Scan samples 37 IAF	Com Scan samples 38 IAF	Com Scan samples 65 IAF

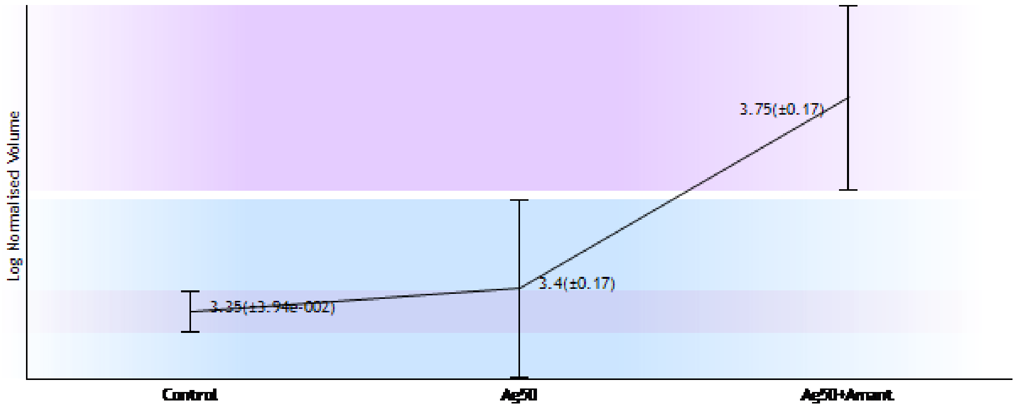

Identifier 470

Position (1117, 718)

Notes

- Anova p-value ≤ 0.05
- Max fold change ≥ 1.5

Control	Ag50	Ag50+Amant
Com Scan samples 37 IAF	Com Scan samples 38 IAF	Com Scan samples 65 IAF

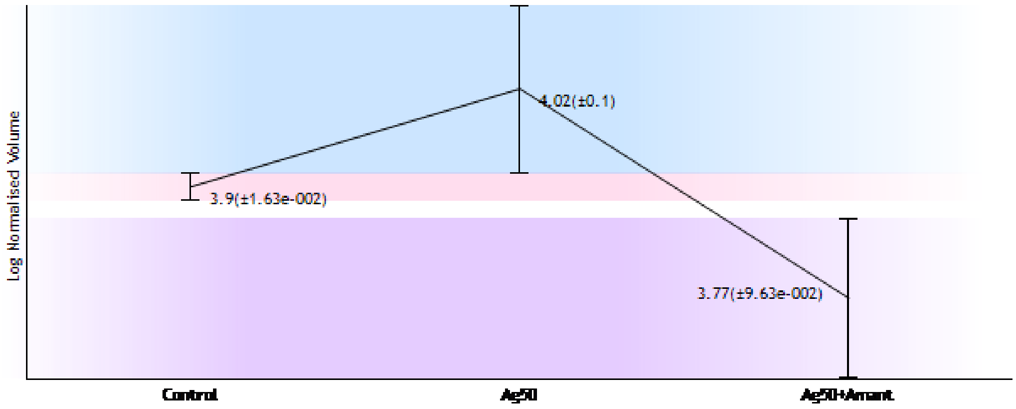

Identifier 397

Position (525, 565)

Notes

- Anova p-value ≤ 0.05
- Max fold change ≥ 1.5

Control	Ag50	Ag50+Amant
Com Scan samples 37 IAF	Com Scan samples 38 IAF	Com Scan samples 65 IAF

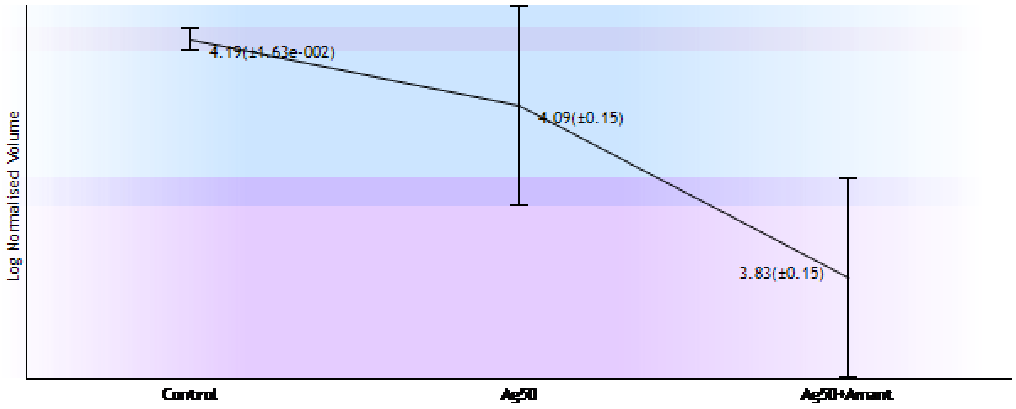

Identifier 181

Position (842, 235)

Notes

- Anova p-value ≤ 0.05
- Max fold change ≥ 1.5

Control	Ag50	Ag50+Amant
Com Scan samples 37 IAF	Com Scan samples 38 IAF	Com Scan samples 65 IAF

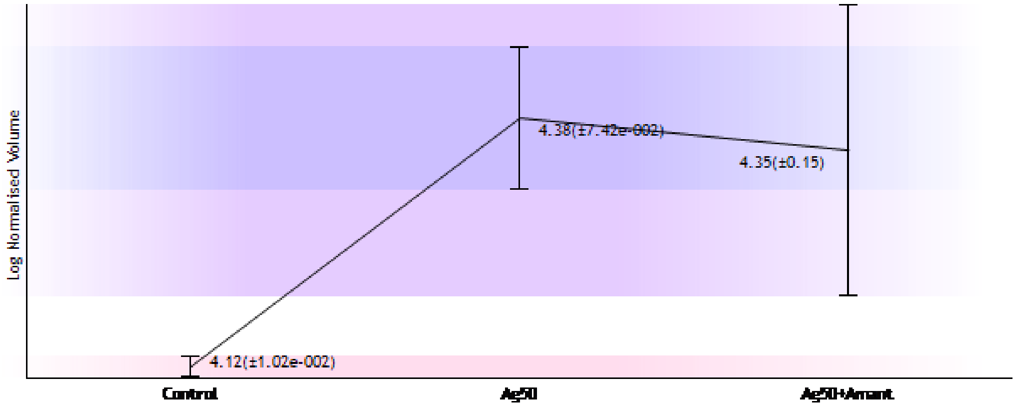

Identifier 143

Position (541, 180)

Notes

- Anova p-value ≤ 0.05
- Max fold change ≥ 1.5

Control	Ag50	Ag50+Amant
Com Scan samples 37 IAF	Com Scan samples 38 IAF	Com Scan samples 65 IAF

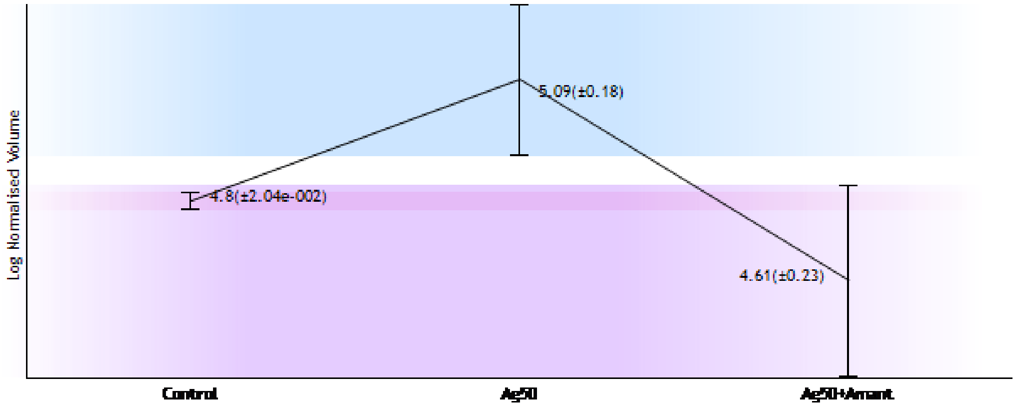

Identifier 122

Position (750, 161)

Notes

- Anova p-value ≤ 0.05
- Max fold change ≥ 1.5

Control	Ag50	Ag50+Amant
Com Scan samples 37 IAF	Com Scan samples 38 IAF	Com Scan samples 65 IAF

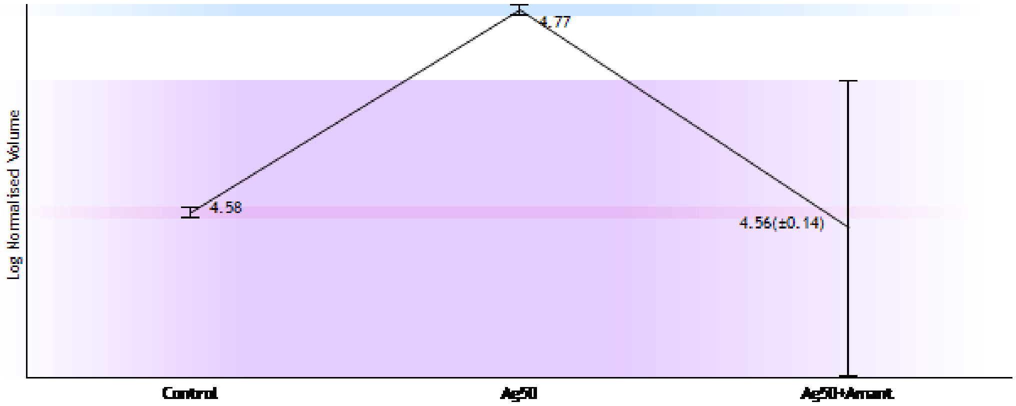

Identifier 424

Position (1009, 624)

Notes

- Anova p-value ≤ 0.05
- Max fold change ≥ 1.5

Control	Ag50	Ag50+Amant
Com Scan samples 37 IAF	Com Scan samples 38 IAF	Com Scan samples 65 IAF

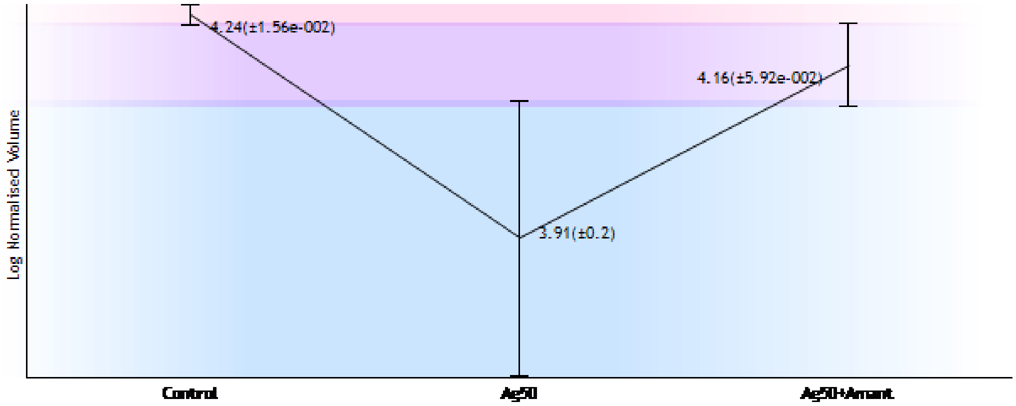

Identifier 219

Position (1010, 279)

Notes

- Anova p-value ≤ 0.05
- Max fold change ≥ 1.5

Control	Ag50	Ag50+Amant
Com Scan samples 37 IAF	Com Scan samples 38 IAF	Com Scan samples 65 IAF

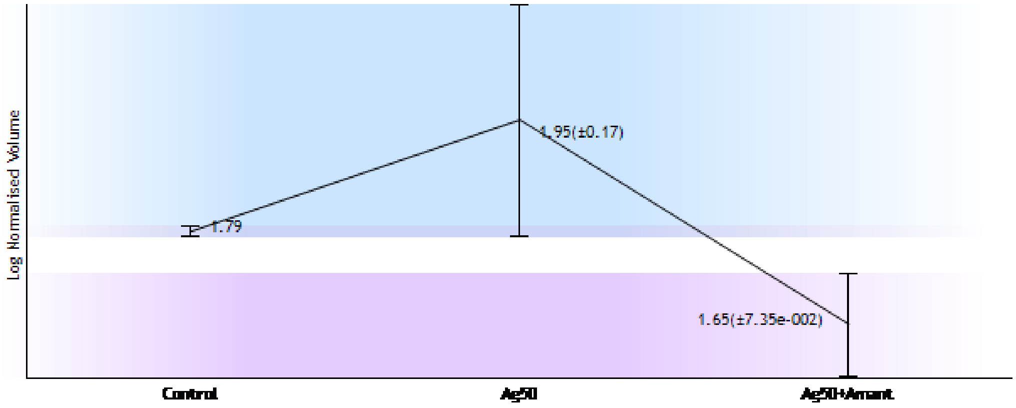

Identifier 353

Position (755, 473)

Notes

- Anova p-value ≤ 0.05
- Max fold change ≥ 1.5

Control	Ag50	Ag50+Amant
Com Scan samples 37 IAF	Com Scan samples 38 IAF	Com Scan samples 65 IAF

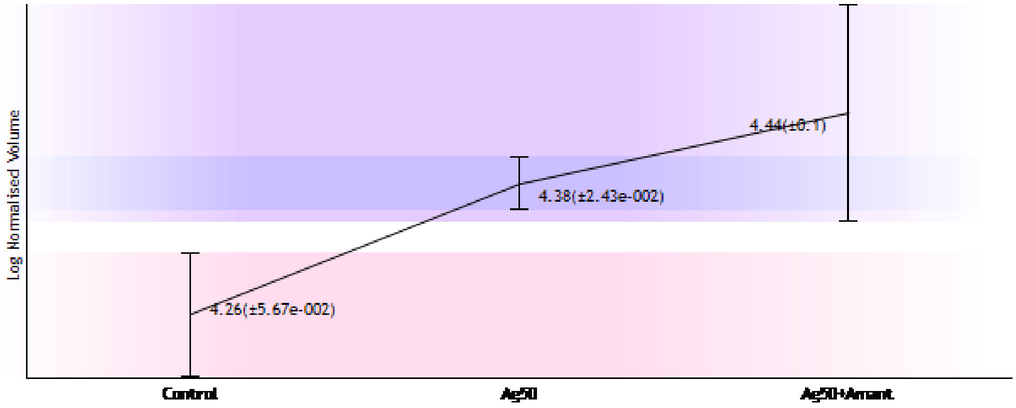

Identifier 390

Position (734, 557)

Notes

- Anova p-value ≤ 0.05
- Max fold change ≥ 1.5

Control	Ag50	Ag50+Amant
Com Scan samples 37 IAF	Com Scan samples 38 IAF	Com Scan samples 65 IAF

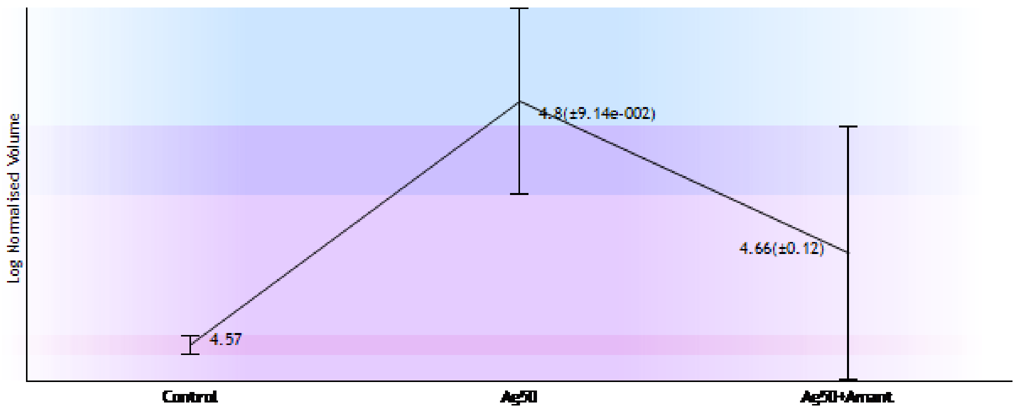

Identifier 539

Position (717, 872)

Notes

- Anova p-value ≤ 0.05
- Max fold change ≥ 1.5

Control	Ag50	Ag50+Amant
Com Scan samples 37 IAF	Com Scan samples 38 IAF	Com Scan samples 65 IAF

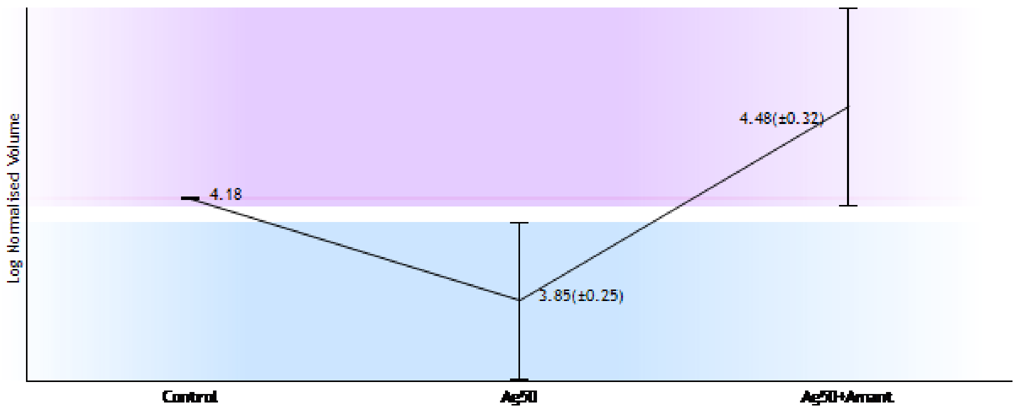

Identifier 209

Position (957, 263)

Notes

- Anova p-value ≤ 0.05
- Max fold change ≥ 1.5

Control	Ag50	Ag50+Amant
Com Scan samples 37 IAF	Com Scan samples 38 IAF	Com Scan samples 65 IAF

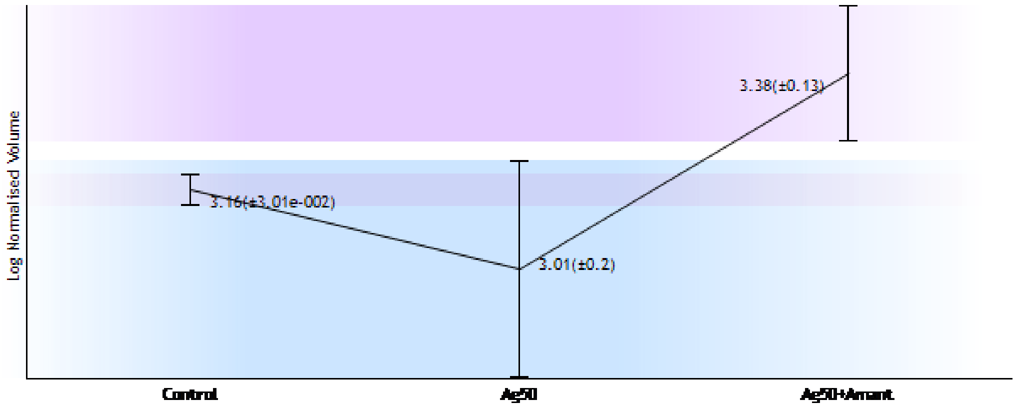

Identifier 485

Position (1156, 735)

Notes

- Anova p-value ≤ 0.05
- Max fold change ≥ 1.5

Control	Ag50	Ag50+Amant
Com Scan samples 37 IAF	Com Scan samples 38 IAF	Com Scan samples 65 IAF

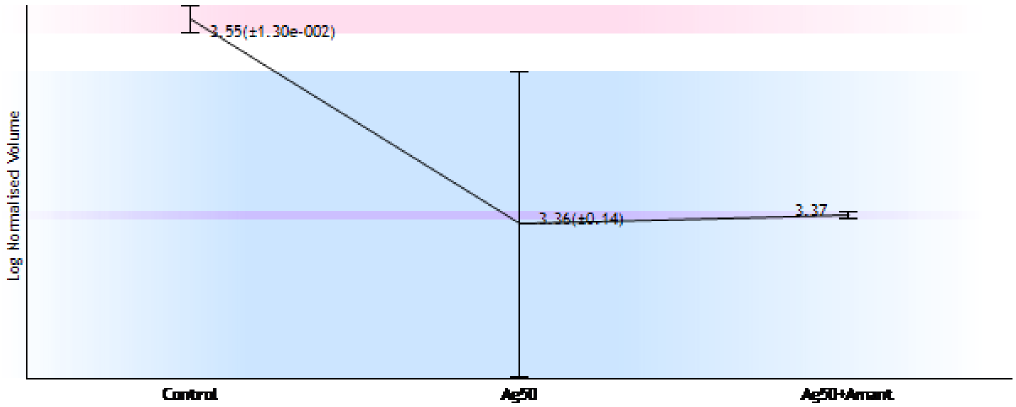

Identifier 185

Position (931, 238)

Notes

- Anova p-value ≤ 0.05
- Max fold change ≥ 1.5

Control	Ag50	Ag50+Amant
Com Scan samples 37 IAF	Com Scan samples 38 IAF	Com Scan samples 65 IAF

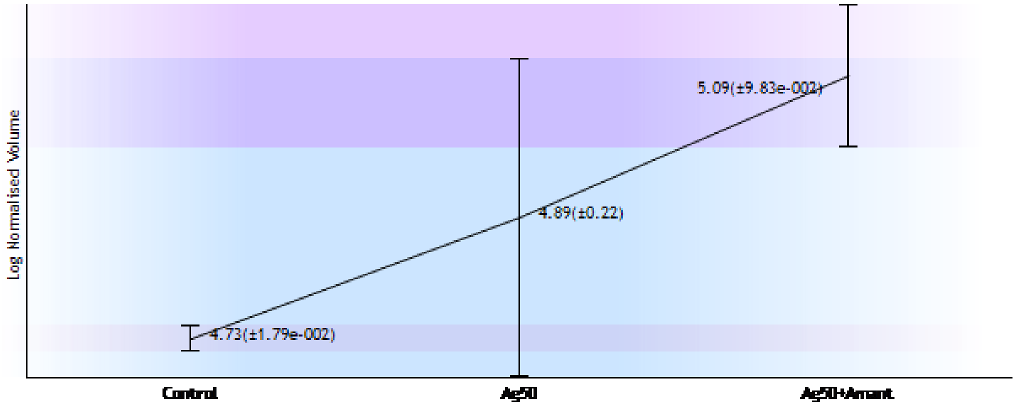

Identifier 543

Position (447, 878)

Notes

- Anova p-value ≤ 0.05
- Max fold change ≥ 1.5

Control	Ag50	Ag50+Amant
Com Scan samples 37 IAF	Com Scan samples 38 IAF	Com Scan samples 65 IAF

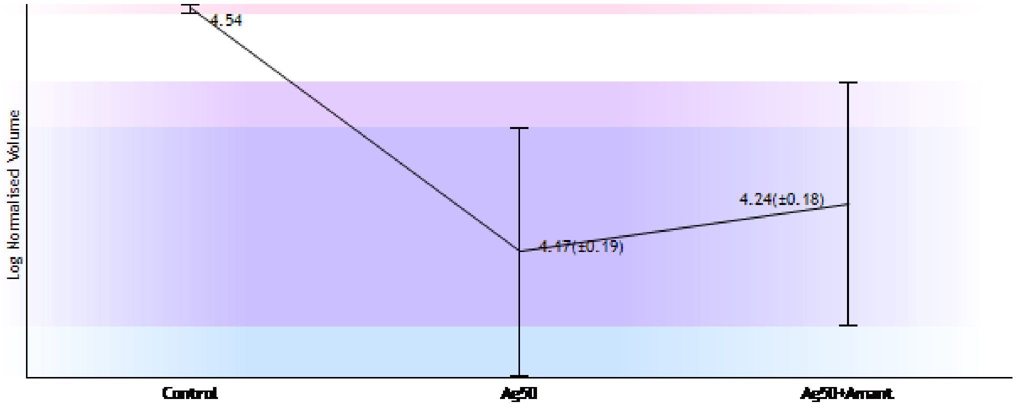

Identifier 127

Position (851, 163)

Notes

- Anova p-value ≤ 0.05
- Max fold change ≥ 1.5

Control	Ag50	Ag50+Amant
Com Scan samples 37 IAF	Com Scan samples 38 IAF	Com Scan samples 65 IAF

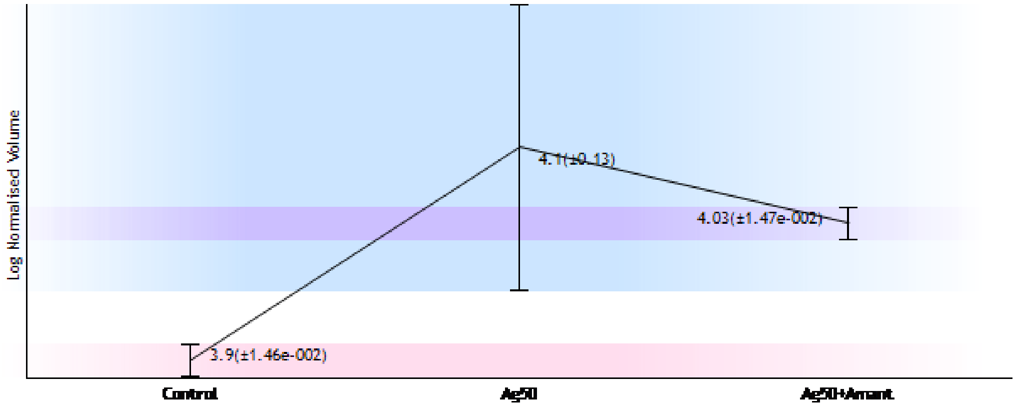

Identifier 413

Position (613, 598)

Notes

- Anova p-value ≤ 0.05
- Max fold change ≥ 1.5

Control	Ag50	Ag50+Amant
Com Scan samples 37 IAF	Com Scan samples 38 IAF	Com Scan samples 65 IAF

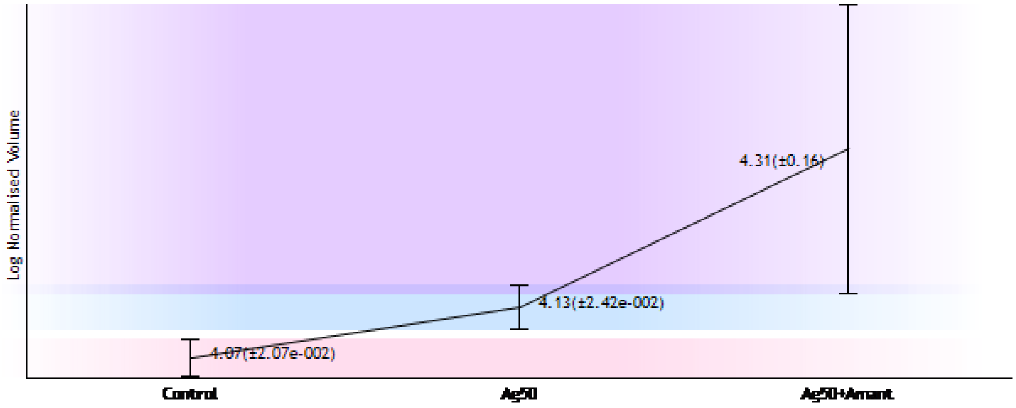

Identifier 479

Position (617, 730)

Notes

- Anova p-value ≤ 0.05
- Max fold change ≥ 1.5

Control	Ag50	Ag50+Amant
Com Scan samples 37 IAF	Com Scan samples 38 IAF	Com Scan samples 65 IAF

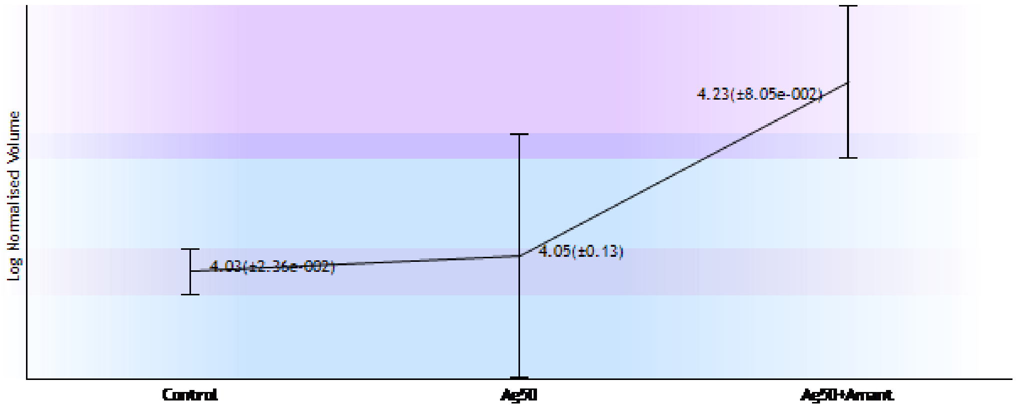

Identifier 412

Position (625, 596)

Notes

- Anova p-value ≤ 0.05
- Max fold change ≥ 1.5

Control	Ag50	Ag50+Amant
Com Scan samples 37 IAF	Com Scan samples 38 IAF	Com Scan samples 65 IAF

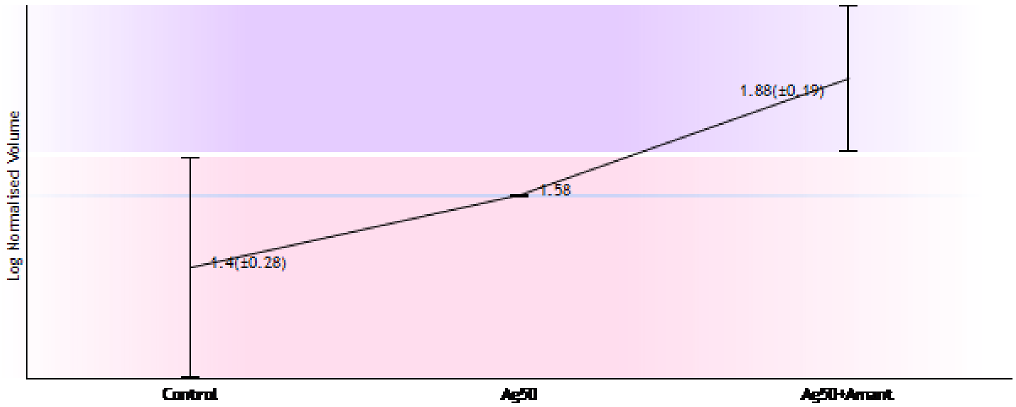

Identifier 432

Position (1027, 643)

Notes

- Anova p-value ≤ 0.05
- Max fold change ≥ 1.5

Control	Ag50	Ag50+Amant
Com Scan samples 37 IAF	Com Scan samples 38 IAF	Com Scan samples 65 IAF

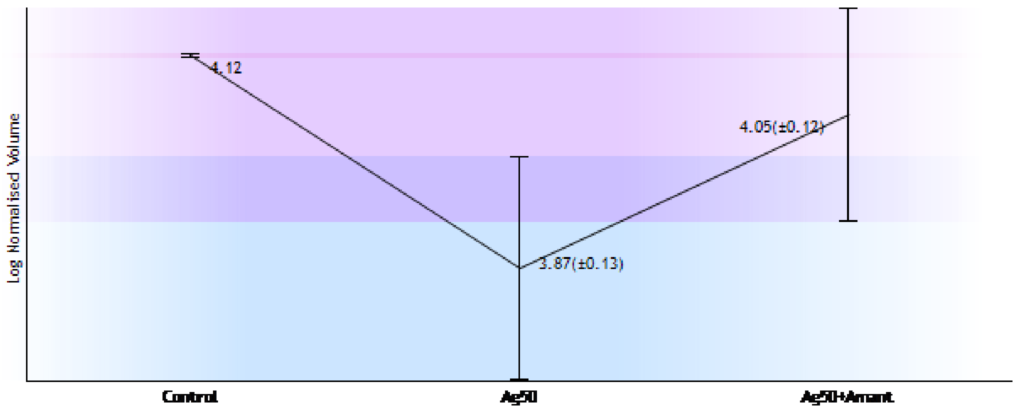

Identifier 439

Position (328, 654)

Notes

- Anova p-value ≤ 0.05
- Max fold change ≥ 1.5

Control	Ag50	Ag50+Amant
Com Scan samples 37 IAF	Com Scan samples 38 IAF	Com Scan samples 65 IAF

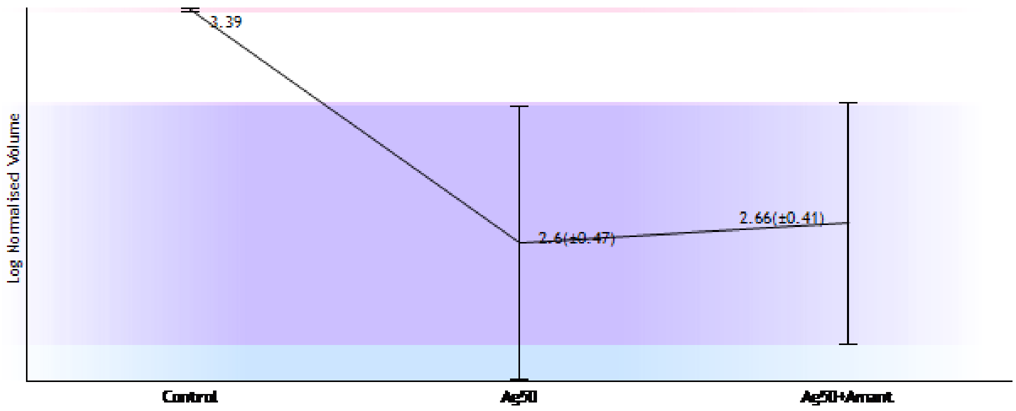

Identifier 52

Position (376, 72)

Notes

- Anova p-value ≤ 0.05
- Max fold change ≥ 1.5

Control	Ag50	Ag50+Amant
Com Scan samples 37 IAF	Com Scan samples 38 IAF	Com Scan samples 65 IAF

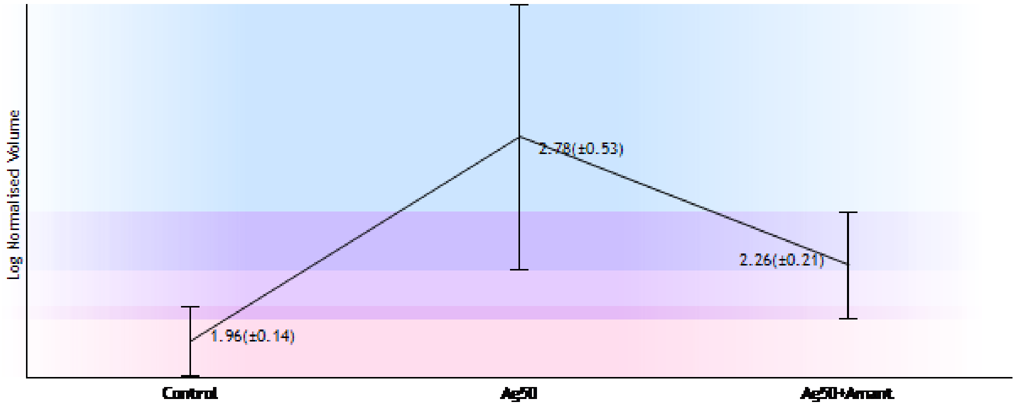

Identifier 166

Position (838, 209)

Notes

- Anova p-value ≤ 0.05
- Max fold change ≥ 1.5

Control	Ag50	Ag50+Amant
Com Scan samples 37 IAF	Com Scan samples 38 IAF	Com Scan samples 65 IAF

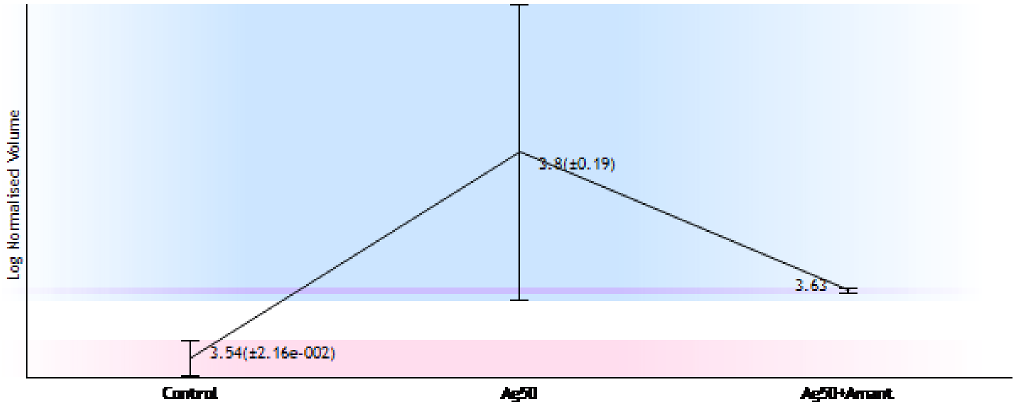

Identifier 340

Position (543, 453)

Notes

- Anova p-value ≤ 0.05
- Max fold change ≥ 1.5

Control	Ag50	Ag50+Amant
Com Scan samples 37 IAF	Com Scan samples 38 IAF	Com Scan samples 65 IAF

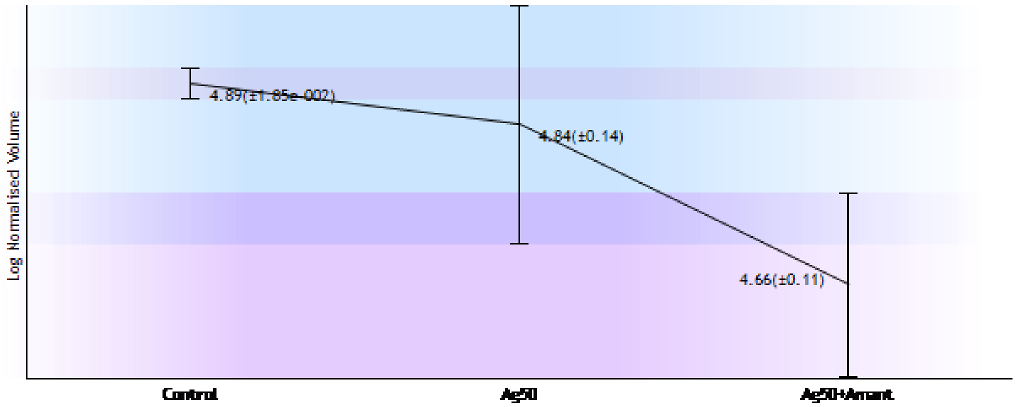

Identifier 198

Position (793, 248)

Notes

- Anova p-value ≤ 0.05
- Max fold change ≥ 1.5

Control	Ag50	Ag50+Amant
Com Scan samples 37 IAF	Com Scan samples 38 IAF	Com Scan samples 65 IAF

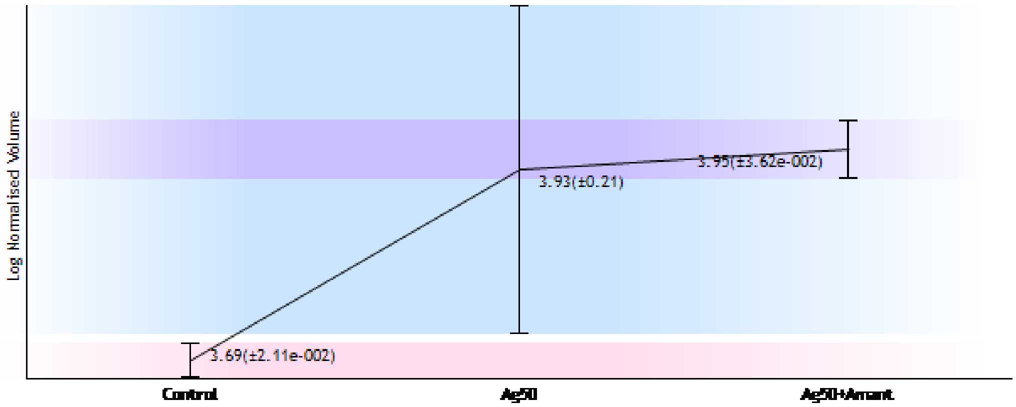

Identifier 176

Position (946, 231)

Notes

- Anova p-value ≤ 0.05
- Max fold change ≥ 1.5

Control	Ag50	Ag50+Amant
Com Scan samples 37 IAF	Com Scan samples 38 IAF	Com Scan samples 65 IAF

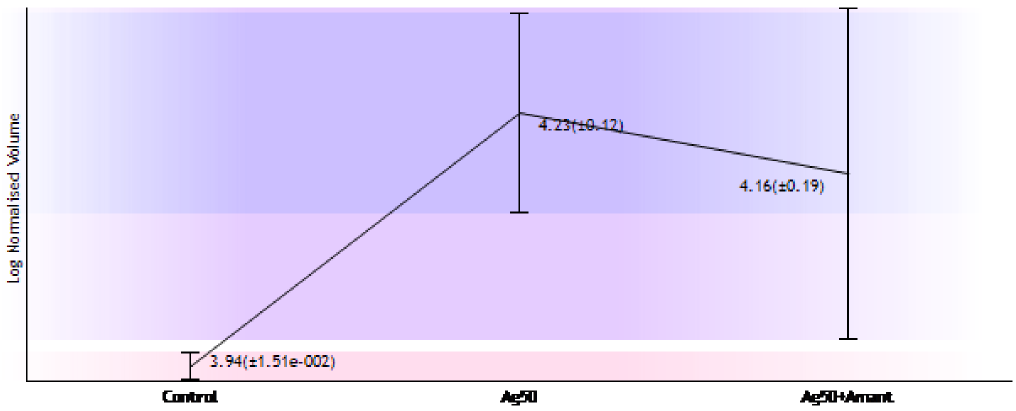

Identifier 446

Position (731, 662)

Notes

- Anova p-value ≤ 0.05
- Max fold change ≥ 1.5

Control	Ag50	Ag50+Amant
Com Scan samples 37 IAF	Com Scan samples 38 IAF	Com Scan samples 65 IAF

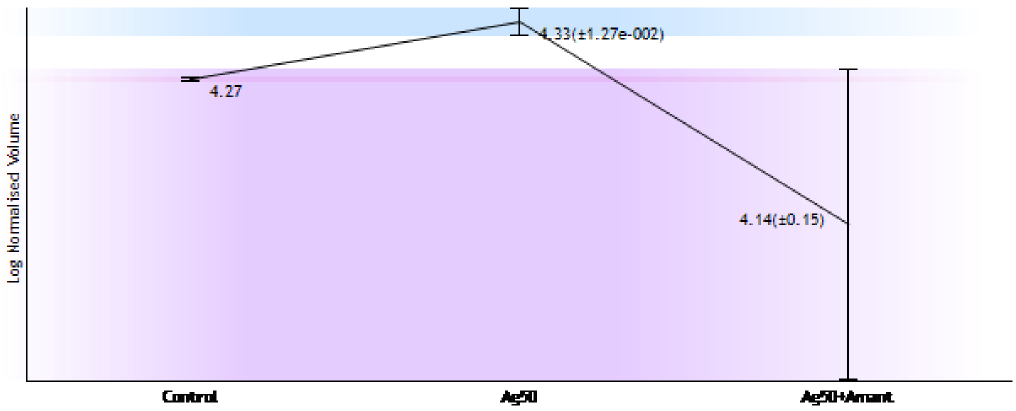

Identifier 234

Position (651, 314)

Notes

- Anova p-value ≤ 0.05
- Max fold change ≥ 1.5

Control	Ag50	Ag50+Amant
Com Scan samples 37 IAF	Com Scan samples 38 IAF	Com Scan samples 65 IAF

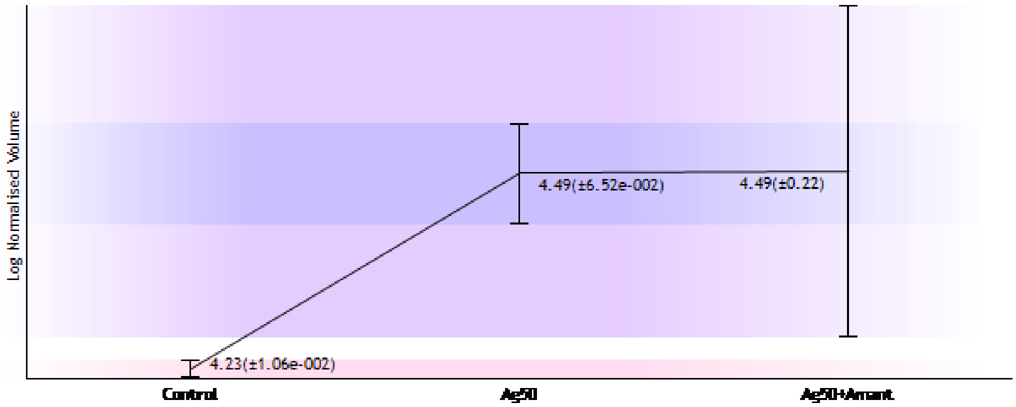

Identifier 160

Position (867, 206)

Notes

- Anova p-value ≤ 0.05
- Max fold change ≥ 1.5

Control	Ag50	Ag50+Amant
Com Scan samples 37 IAF	Com Scan samples 38 IAF	Com Scan samples 65 IAF

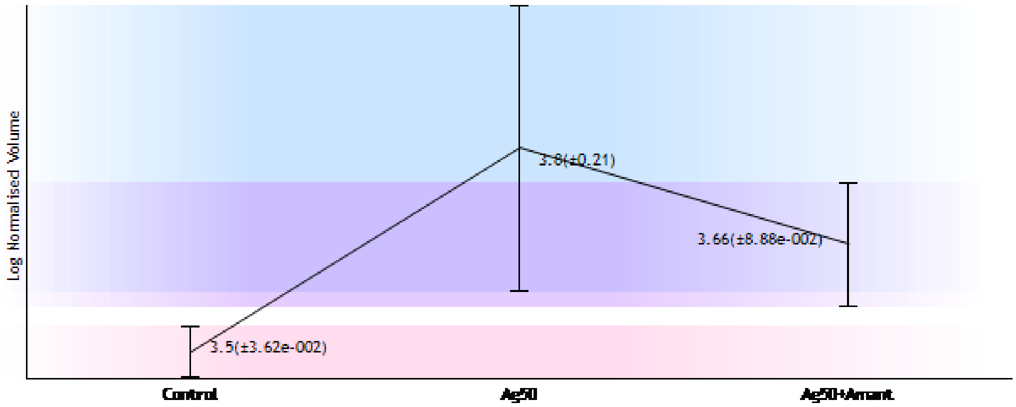

Identifier 535

Position (860, 862)

Notes

- Anova p-value ≤ 0.05
- Max fold change ≥ 1.5

Control	Ag50	Ag50+Amant
Com Scan samples 37 IAF	Com Scan samples 38 IAF	Com Scan samples 65 IAF

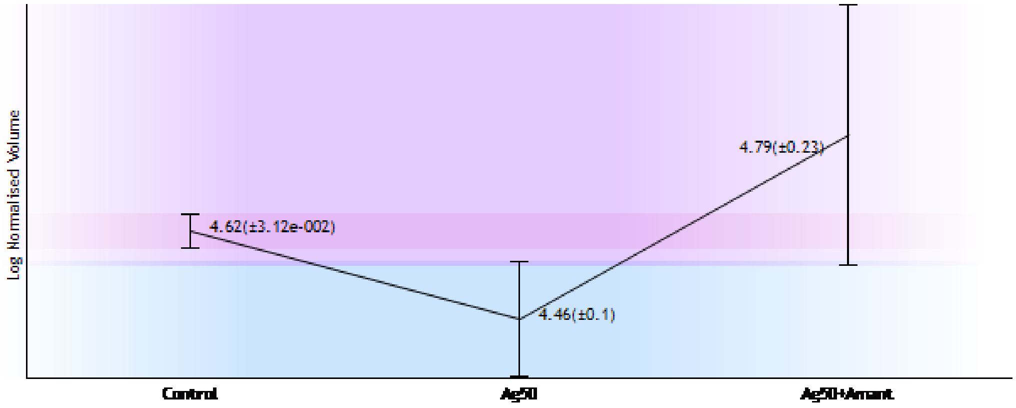

Identifier 484

Position (645, 734)

Notes

- Anova p-value ≤ 0.05
- Max fold change ≥ 1.5

Control	Ag50	Ag50+Amant
Com Scan samples 37 IAF	Com Scan samples 38 IAF	Com Scan samples 65 IAF

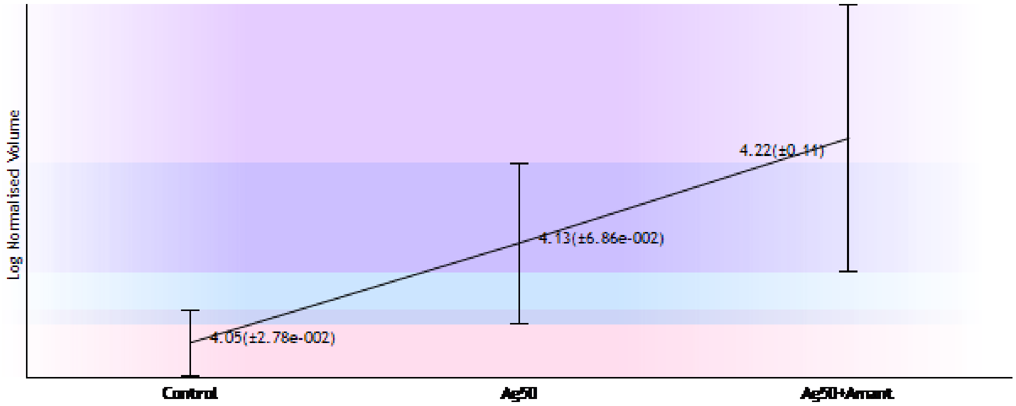

Identifier 305

Position (352, 398)

Notes

- Anova p-value ≤ 0.05
- Max fold change ≥ 1.5

Control	Ag50	Ag50+Amant
Com Scan samples 37 IAF	Com Scan samples 38 IAF	Com Scan samples 65 IAF

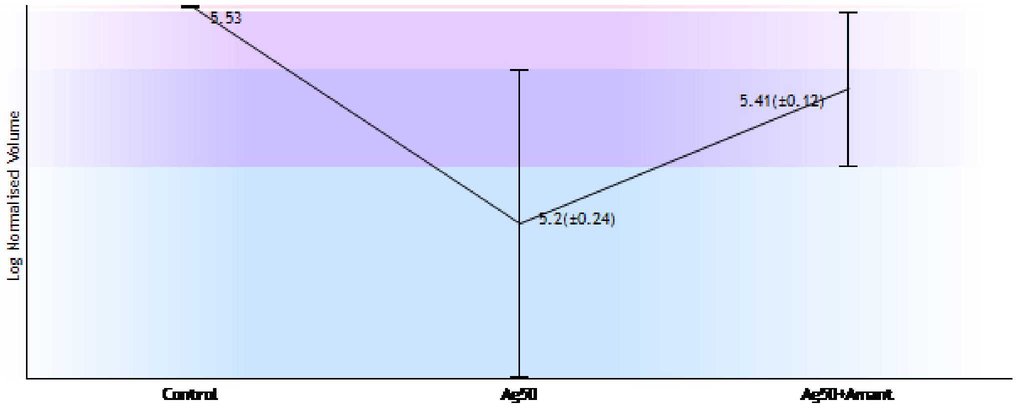

Identifier 385

Position (957, 546)

Notes

- Anova p-value ≤ 0.05
- Max fold change ≥ 1.5

Control	Ag50	Ag50+Amant
Com Scan samples 37 IAF	Com Scan samples 38 IAF	Com Scan samples 65 IAF

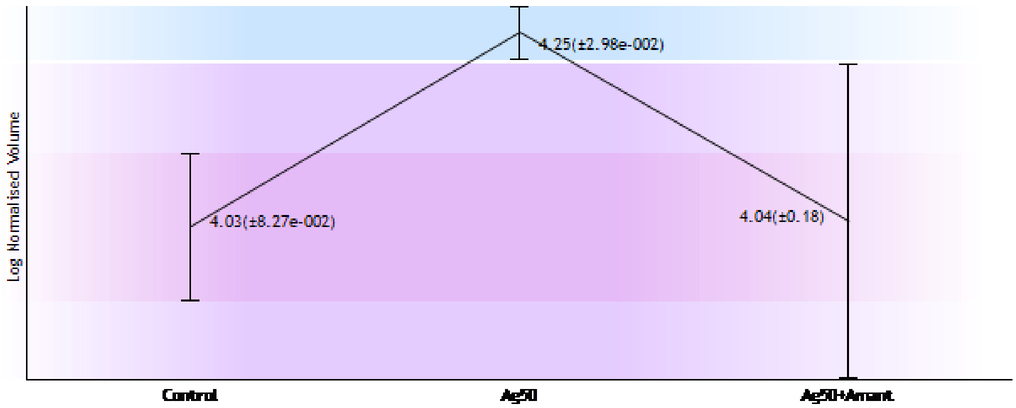

Identifier 540

Position (1172, 873)

Notes

- Anova p-value ≤ 0.05
- Max fold change ≥ 1.5

Control	Ag50	Ag50+Amant
Com Scan samples 37 IAF	Com Scan samples 38 IAF	Com Scan samples 65 IAF

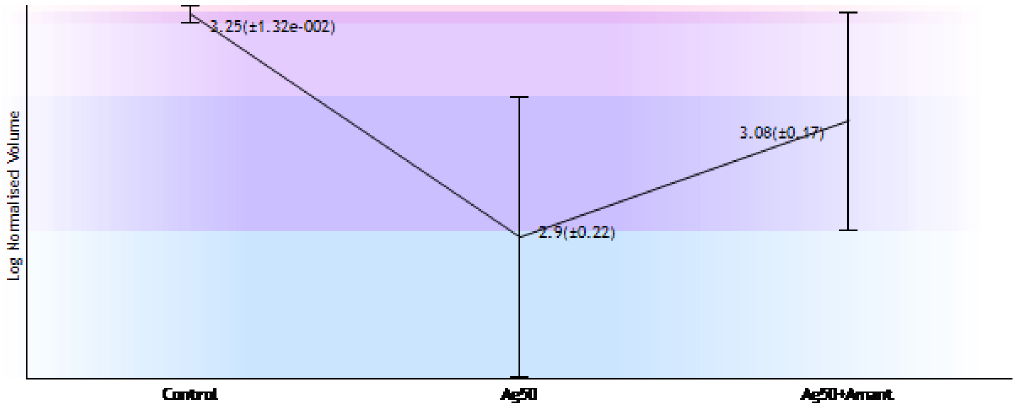

Identifier 91

Position (867, 121)

Notes

- Anova p-value ≤ 0.05
- Max fold change ≥ 1.5

Control	Ag50	Ag50+Amant
Com Scan samples 37 IAF	Com Scan samples 38 IAF	Com Scan samples 65 IAF

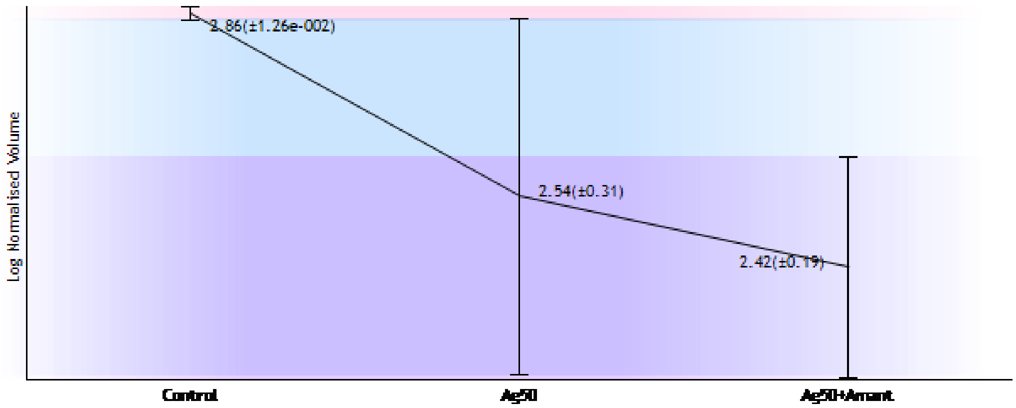

Identifier 399

Position (539, 567)

Notes

- Anova p-value ≤ 0.05
- Max fold change ≥ 1.5

Control	Ag50	Ag50+Amant
Com Scan samples 37 IAF	Com Scan samples 38 IAF	Com Scan samples 65 IAF

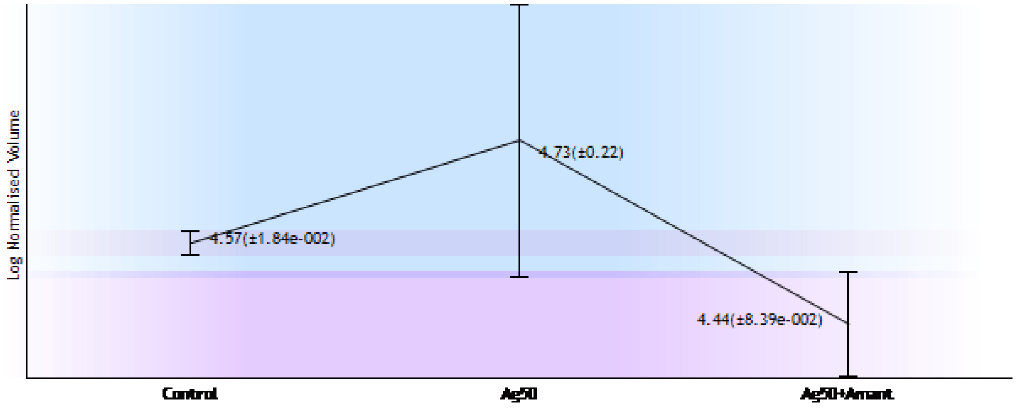

Identifier 71

Position (540, 96)

Notes

- Anova p-value ≤ 0.05
- Max fold change ≥ 1.5

Control	Ag50	Ag50+Amant
Com Scan samples 37 IAF	Com Scan samples 38 IAF	Com Scan samples 65 IAF

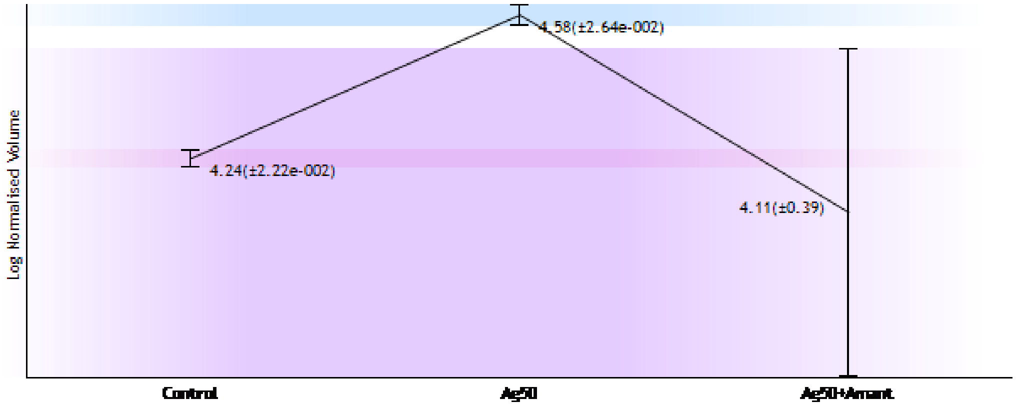

Identifier 173

Position (846, 218)

Notes

- Anova p-value ≤ 0.05
- Max fold change ≥ 1.5

Control	Ag50	Ag50+Amant
Com Scan samples 37 IAF	Com Scan samples 38 IAF	Com Scan samples 65 IAF

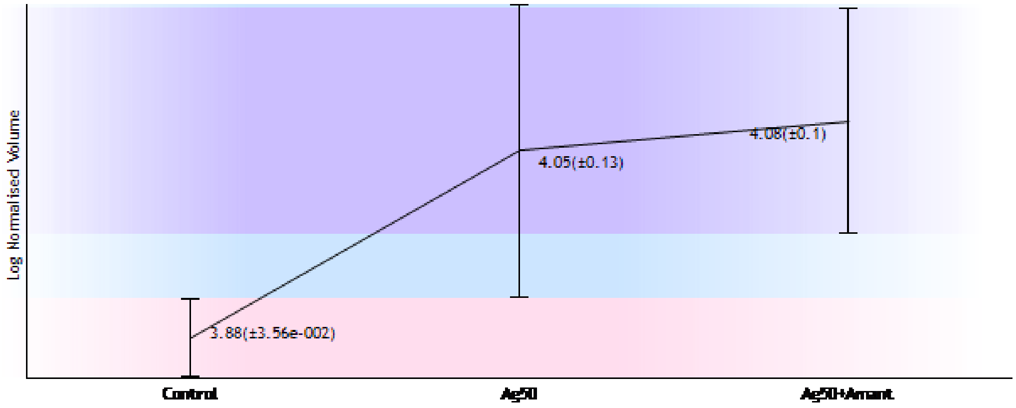

Identifier 268

Position (899, 355)

Notes

- Anova p-value ≤ 0.05
- Max fold change ≥ 1.5

Control	Ag50	Ag50+Amant
Com Scan samples 37 IAF	Com Scan samples 38 IAF	Com Scan samples 65 IAF

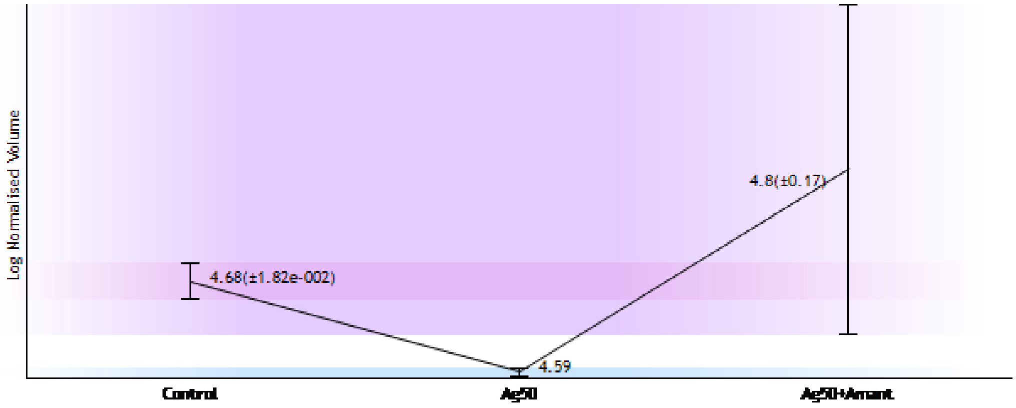

Identifier 414

Position (763, 600)

Notes

- Anova p-value ≤ 0.05
- Max fold change ≥ 1.5

Control	Ag50	Ag50+Amant
Com Scan samples 37 IAF	Com Scan samples 38 IAF	Com Scan samples 65 IAF

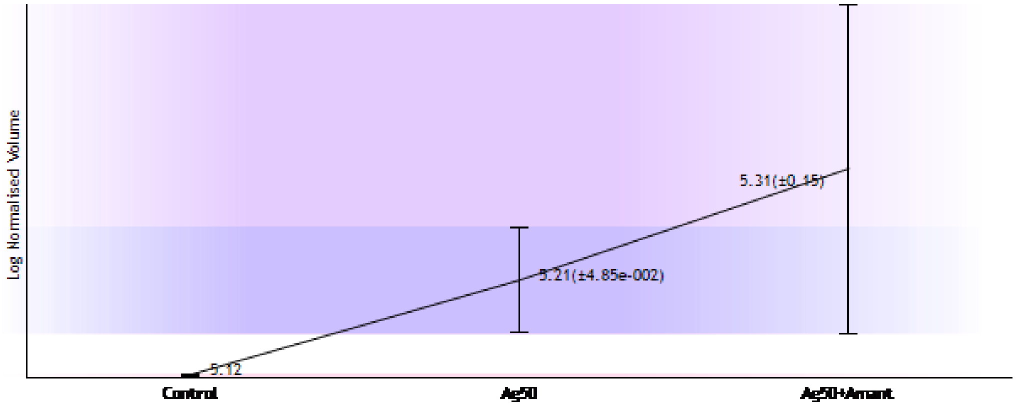

Identifier 55

Position (542, 74)

Notes

- Anova p-value ≤ 0.05
- Max fold change ≥ 1.5

Control	Ag50	Ag50+Amant
Com Scan samples 37 IAF	Com Scan samples 38 IAF	Com Scan samples 65 IAF

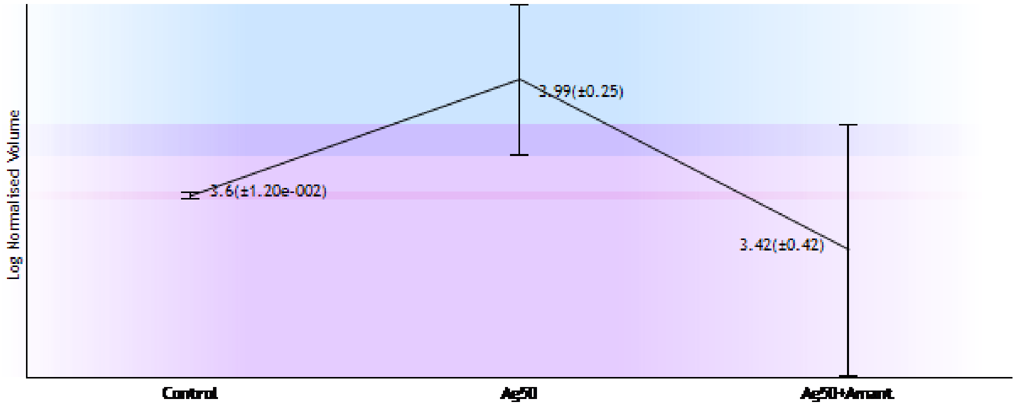

Younes Comm FTSC Gills

Experiment: Younes Comm FTSC Gills

Report created: 22/12/2015 12:18:55

Reference image

Experiment Design

Condition	Control	Ag50	Ag50+Amant
Replicates	2	2	2

Spots

#	Anova (p)	Fold	Tags	Notes	pI	MW	Protein Accession	Protein Description	Protein pI	Protein MW	Protein URL	Average Normalised Volumes		
												Control	Ag50	Ag50+Amant
320	3.531e-004	2.8										3519.223	5628.040	1983.307
238	0.002	1.9										7.442e+004	6.919e+004	3.998e+004
184	0.003	1.8										2.141e+004	3.934e+004	3.692e+004
220	0.005	4.8										5494.209	2.662e+004	1.948e+004
122	0.005	2.7										6910.671	1.891e+004	1.872e+004
162	0.006	1.5										572.182	882.553	612.822
132	0.006	2.2										2367.546	4254.008	5102.760
142	0.006	3.9										1757.939	6158.747	6884.593
53	0.007	3.7										1675.825	2465.367	672.592
126	0.008	4.6										1121.850	1879.291	5209.214
134	0.009	3.5										1796.200	5118.961	6267.776
56	0.010	6.0										1465.746	1628.256	273.496
21	0.010	2.7										9738.170	1.657e+004	6170.673
315	0.012	2.1										2.486e+004	1.166e+004	1.749e+004
348	0.013	1.8										230.624	223.921	129.428
70	0.014	2.5										5537.231	7579.573	1.376e+004
216	0.016	2.5										1.760e+004	4.333e+004	2.750e+004
296	0.016	1.8										2.028e+004	1.343e+004	1.117e+004
123	0.016	3.3										1215.660	961.153	3184.014
297	0.016	1.9										2.766e+004	1.573e+004	2.956e+004
125	0.017	4.7										6532.094	2.073e+004	3.100e+004
52	0.020	6.3										1907.347	3630.385	1.195e+004
201	0.021	2.2										1.150e+005	8.032e+004	5.307e+004
434	0.023	2.2										3565.290	2222.131	1591.795
313	0.024	1.9										6500.279	1.213e+004	1.195e+004
275	0.024	1.8										6582.333	4846.176	8850.067
230	0.025	5.5										2.895e+004	2.721e+004	5282.074
210	0.025	2.3										1.257e+004	1.330e+004	5737.759

#	Anova (p)	Fold	Tags	Notes	pI	MW	Protein Accession	Protein Description	Protein pI	Protein MW	Protein URL	Average Normalised Volumes		
												Control	Ag50	Ag50+Amant
432	0.025	1.9										2.715e+005	1.477e+005	1.394e+005
133	0.026	2.9										4424.643	7405.189	1.281e+004
396	0.026	1.6										1982.678	1237.114	1305.195
436	0.037	2.2										8.741e+004	4.860e+004	4.011e+004
77	0.037	2.7										1645.818	4487.280	3312.166
124	0.038	6.2										2878.230	8505.389	1.770e+004
72	0.039	3.8										1.682e+004	1.785e+004	6.319e+004
395	0.041	3.5										3557.733	1016.366	1156.640
207	0.043	2.5										1.462e+004	1.330e+004	3.332e+004
135	0.043	2.7										5087.593	2215.560	5889.903
331	0.043	2.4										2897.516	5812.754	7037.758
358	0.043	2.0										1.358e+004	6821.654	8980.947
378	0.044	1.6										2430.136	1838.494	1502.847
374	0.048	1.6										5053.880	4396.088	6980.712
43	0.048	2.6										176.251	270.621	460.722
81	0.049	1.7										1665.298	2784.355	1615.682
128	0.049	2.1										1231.879	2405.537	2587.670

Tags	
	Anova p-value ≤ 0.05
	Max fold change ≥ 1.5

Identifier 320

Position (544, 602)

Notes

Anova p-value ≤ 0.05

Max fold change ≥ 1.5

Control	Ag50	Ag50+Amant
Com Scan samples 1FTSC	Com Scan samples 2 FTSC	Com Scan samples 29 FTSC

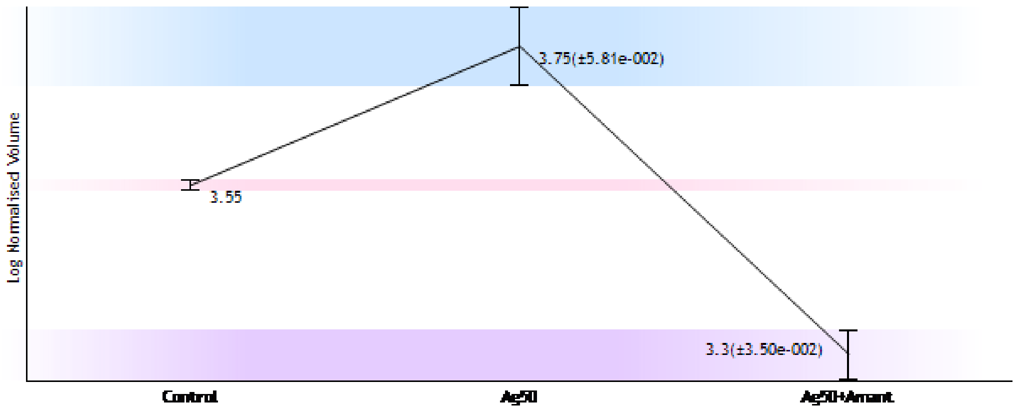

Identifier 238

Position (982, 442)

Notes

Anova p-value ≤ 0.05
Max fold change ≥ 1.5

Control	Ag50	Ag50+Amant
Com Scan samples 1FTSC	Com Scan samples 2 FTSC	Com Scan samples 29 FTSC

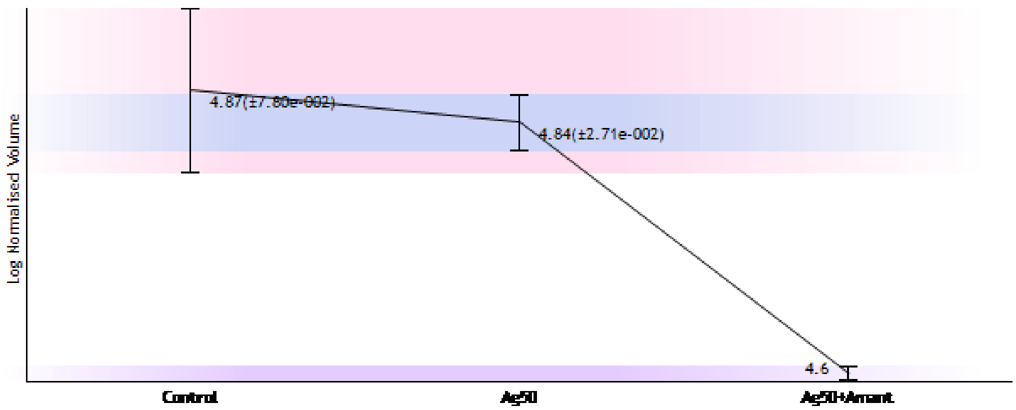

Identifier 184

Position (783, 379)

Notes

Anova p-value ≤ 0.05
Max fold change ≥ 1.5

Control	Ag50	Ag50+Amant
Com Scan samples 1FTSC	Com Scan samples 2 FTSC	Com Scan samples 29 FTSC

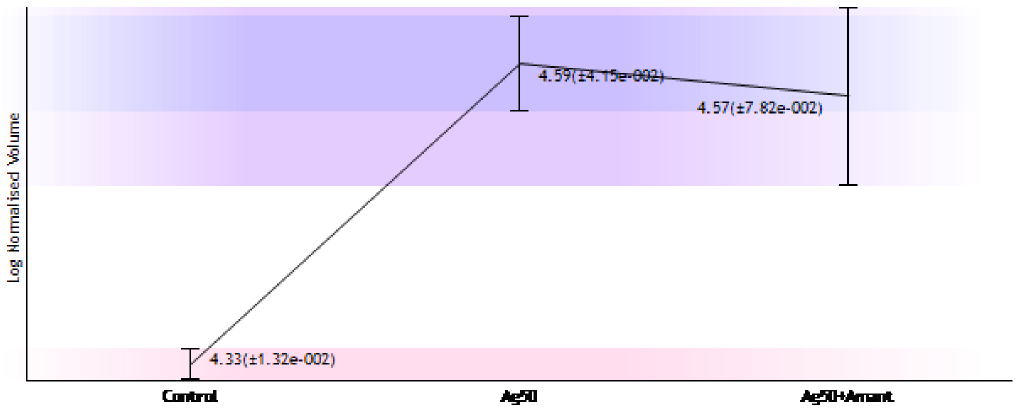

Identifier 220

Position (767, 427)

Notes

Anova p-value ≤ 0.05
Max fold change ≥ 1.5

Control	Ag50	Ag50+Amant
Com Scan samples 1FTSC	Com Scan samples 2 FTSC	Com Scan samples 29 FTSC

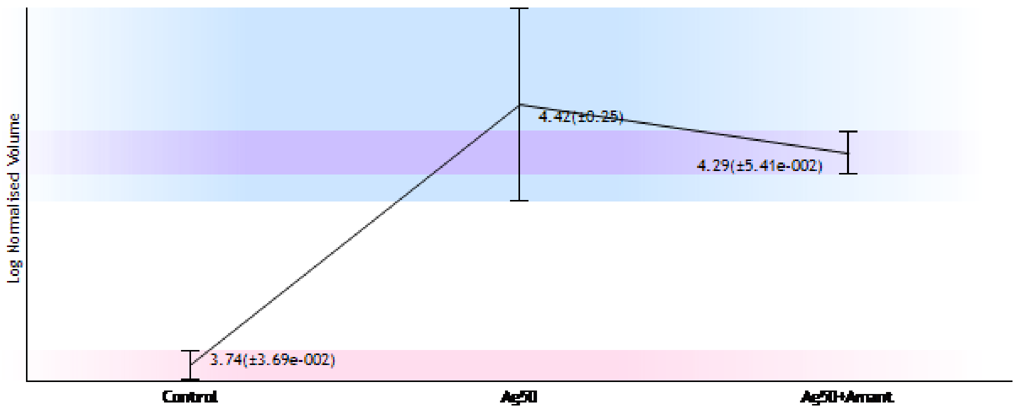

Identifier 122

Position (618, 300)

Notes

- Anova p-value ≤ 0.05
- Max fold change ≥ 1.5

Control	Ag50	Ag50+Amant
Com Scan samples 1FTSC	Com Scan samples 2 FTSC	Com Scan samples 29 FTSC

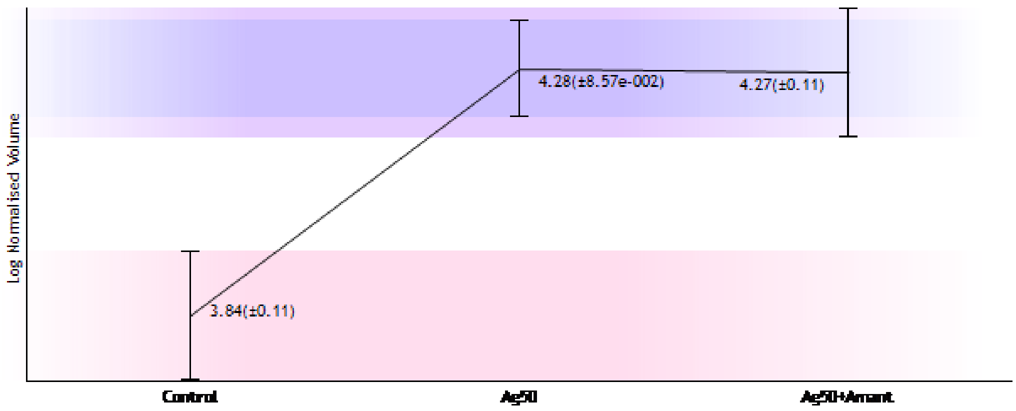

Identifier 162

Position (723, 354)

Notes

- Anova p-value ≤ 0.05
- Max fold change ≥ 1.5

Control	Ag50	Ag50+Amant
Com Scan samples 1FTSC	Com Scan samples 2 FTSC	Com Scan samples 29 FTSC

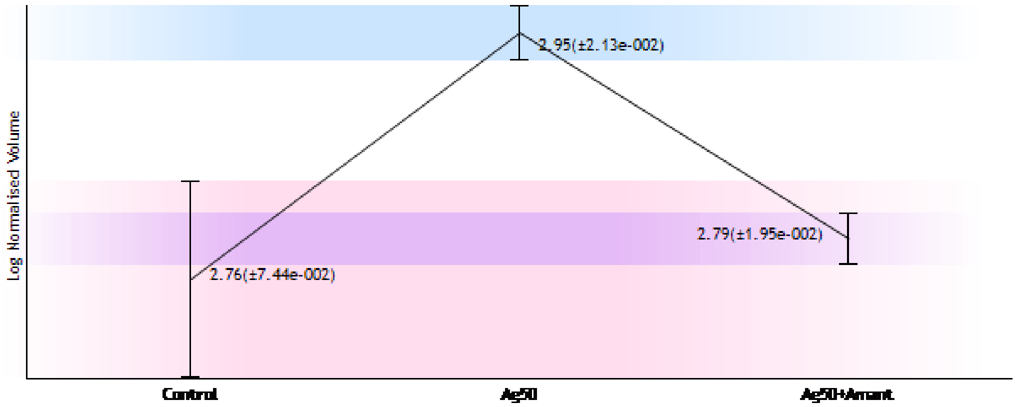

Identifier 132

Position (788, 308)

Notes

- Anova p-value ≤ 0.05
- Max fold change ≥ 1.5

Control	Ag50	Ag50+Amant
Com Scan samples 1FTSC	Com Scan samples 2 FTSC	Com Scan samples 29 FTSC

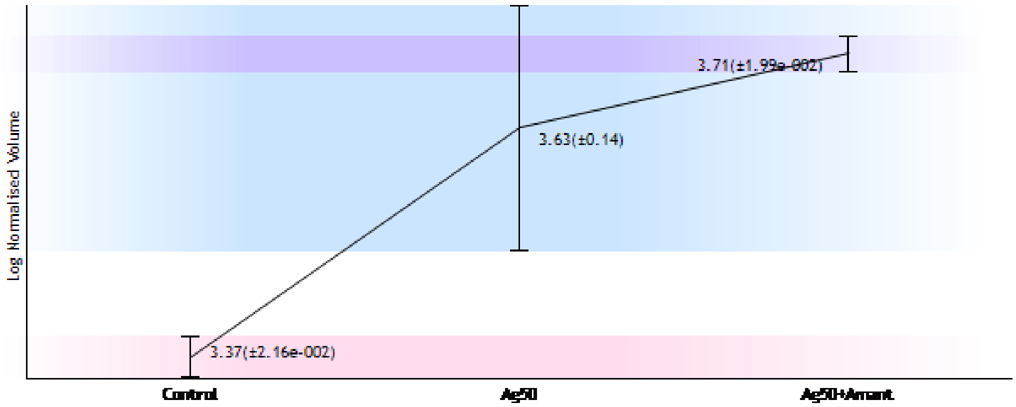

Identifier 142

Position (739, 337)

Notes

- Anova p-value ≤ 0.05
- Max fold change ≥ 1.5

Control	Ag50	Ag50+Amant
Com Scan samples 1FTSC	Com Scan samples 2 FTSC	Com Scan samples 29 FTSC

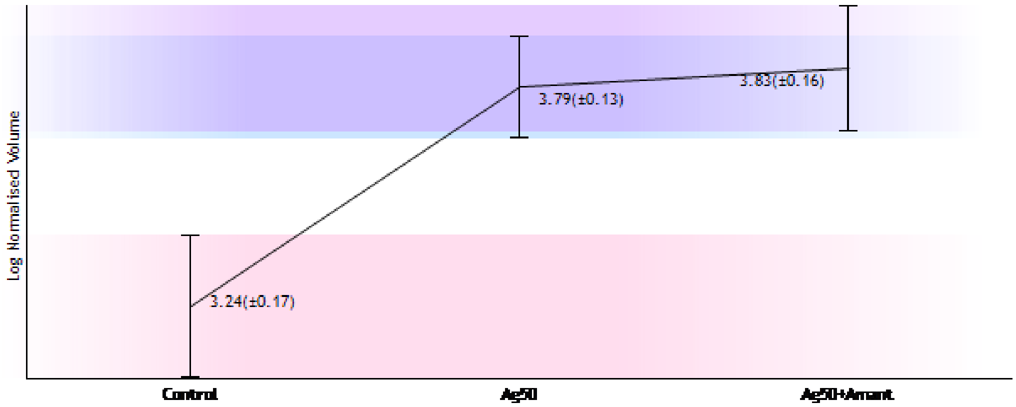

Identifier 53

Position (1181, 222)

Notes

- Anova p-value ≤ 0.05
- Max fold change ≥ 1.5

Control	Ag50	Ag50+Amant
Com Scan samples 1FTSC	Com Scan samples 2 FTSC	Com Scan samples 29 FTSC

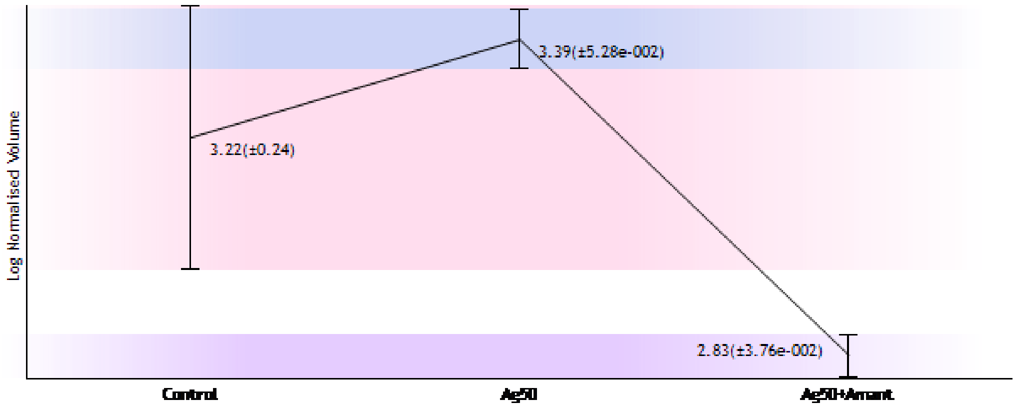

Identifier 126

Position (567, 302)

Notes

- Anova p-value ≤ 0.05
- Max fold change ≥ 1.5

Control	Ag50	Ag50+Amant
Com Scan samples 1FTSC	Com Scan samples 2 FTSC	Com Scan samples 29 FTSC

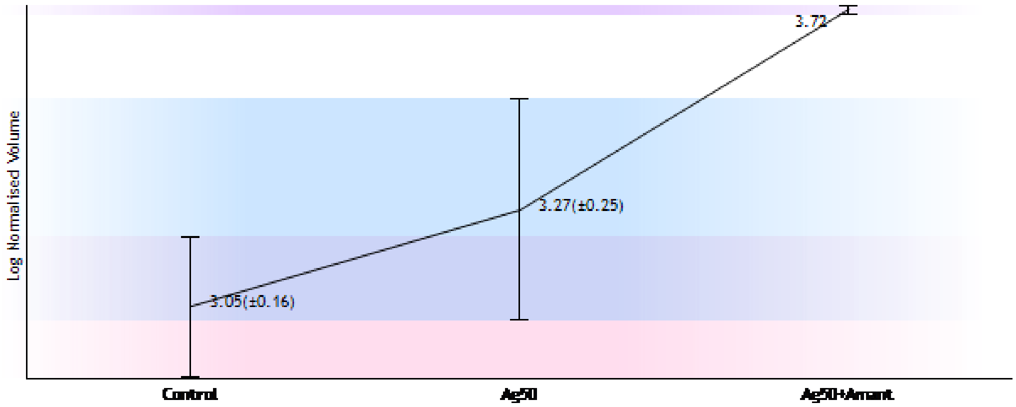

Identifier 134

Position (582, 312)

Notes

- Anova p-value ≤ 0.05
- Max fold change ≥ 1.5

Control	Ag50	Ag50+Amant
Com Scan samples 1FTSC	Com Scan samples 2 FTSC	Com Scan samples 29 FTSC

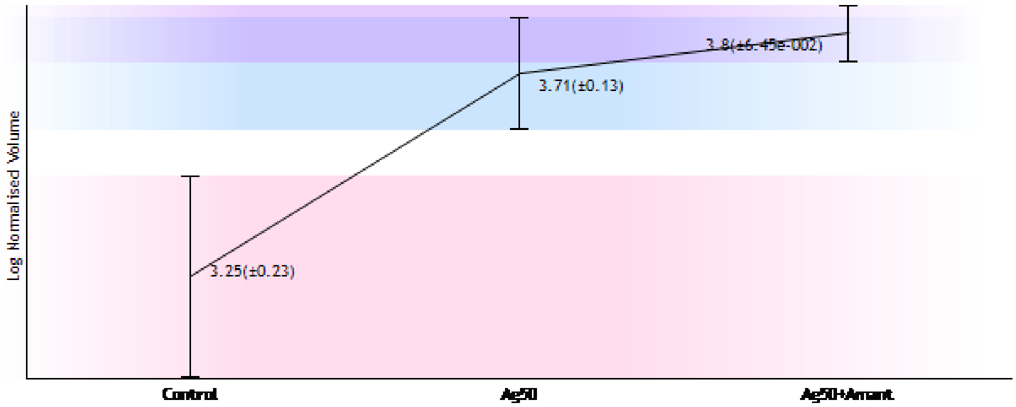

Identifier 56

Position (1173, 223)

Notes

- Anova p-value ≤ 0.05
- Max fold change ≥ 1.5

Control	Ag50	Ag50+Amant
Com Scan samples 1FTSC	Com Scan samples 2 FTSC	Com Scan samples 29 FTSC

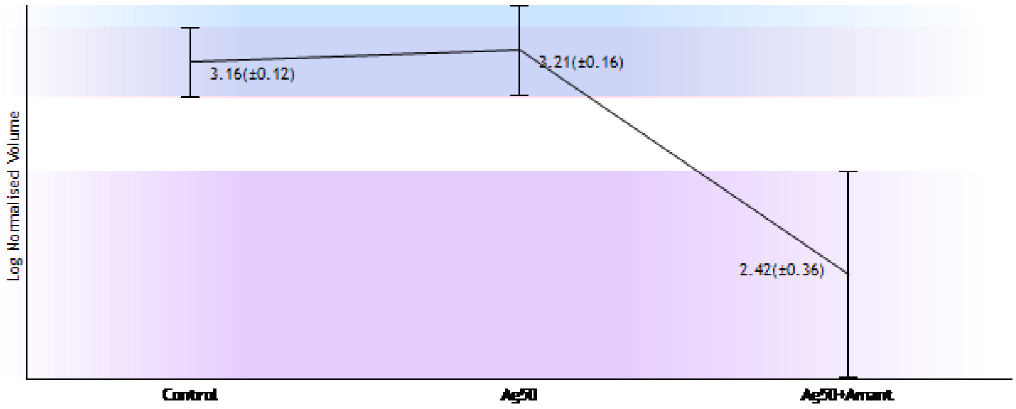

Identifier 21

Position (1255, 154)

Notes

- Anova p-value ≤ 0.05
- Max fold change ≥ 1.5

Control	Ag50	Ag50+Amant
Com Scan samples 1FTSC	Com Scan samples 2 FTSC	Com Scan samples 29 FTSC

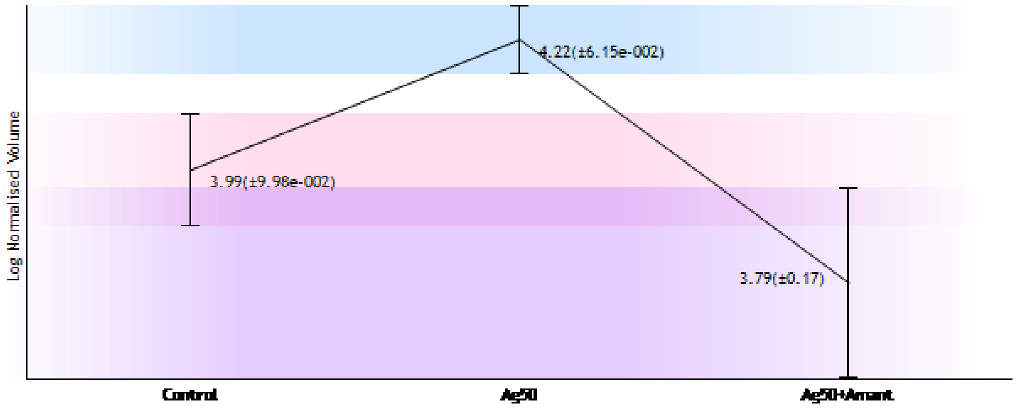

Identifier 315

Position (898, 595)

Notes

- Anova p-value ≤ 0.05
- Max fold change ≥ 1.5

Control	Ag50	Ag50+Amant
Com Scan samples 1FTSC	Com Scan samples 2 FTSC	Com Scan samples 29 FTSC

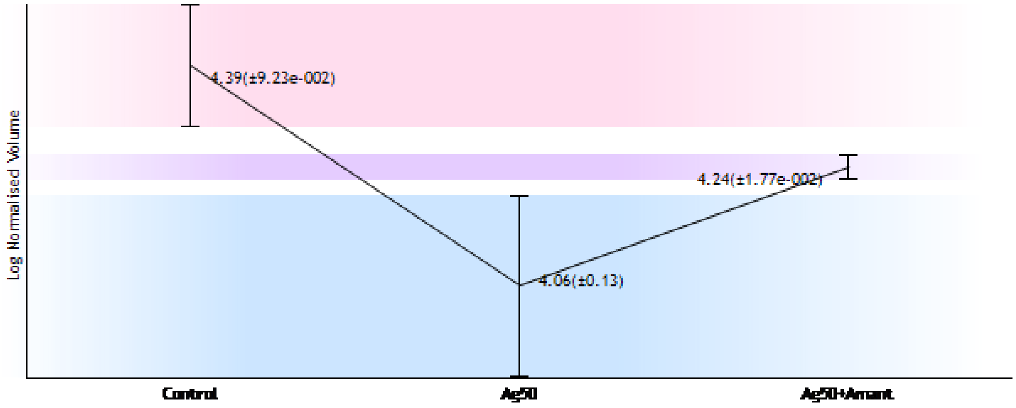

Identifier 348

Position (1174, 656)

Notes

- Anova p-value ≤ 0.05
- Max fold change ≥ 1.5

Control	Ag50	Ag50+Amant
Com Scan samples 1FTSC	Com Scan samples 2 FTSC	Com Scan samples 29 FTSC

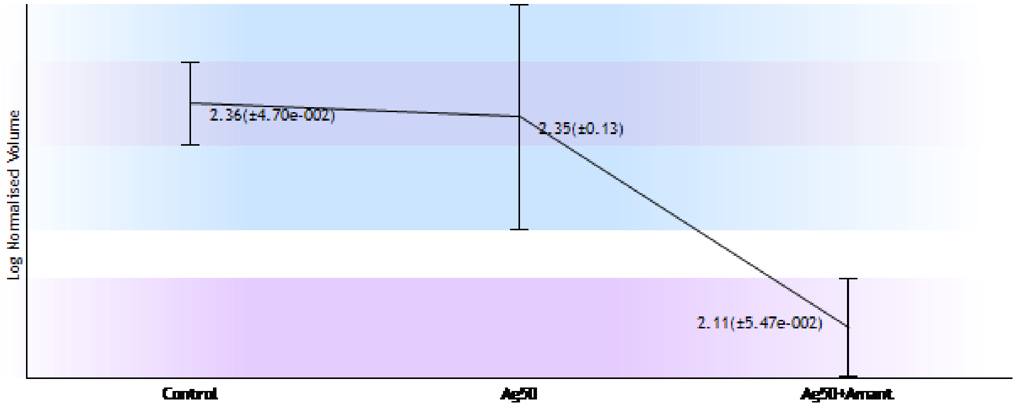

Identifier 70

Position (959, 235)

Notes

- Anova p-value ≤ 0.05
- Max fold change ≥ 1.5

Control	Ag50	Ag50+Amant
Com Scan samples 1FTSC	Com Scan samples 2 FTSC	Com Scan samples 29 FTSC

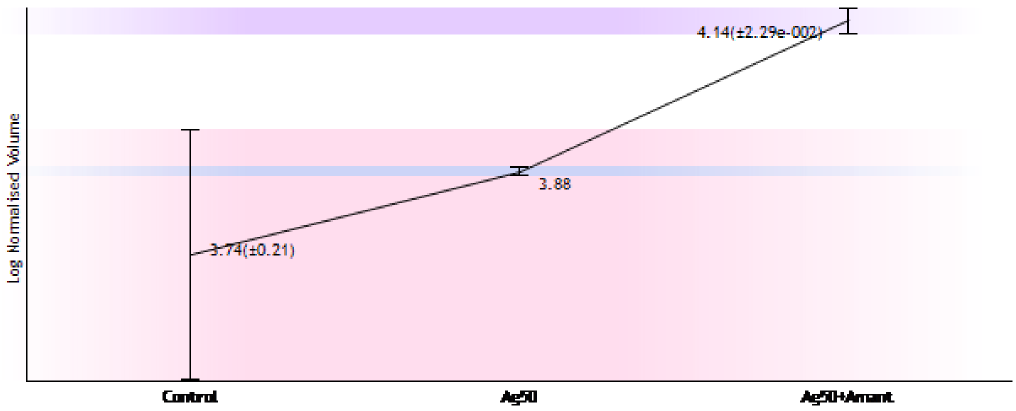

Identifier 216

Position (735, 424)

Notes

- Anova p-value ≤ 0.05
- Max fold change ≥ 1.5

Control	Ag50	Ag50+Amant
Com Scan samples 1FTSC	Com Scan samples 2 FTSC	Com Scan samples 29 FTSC

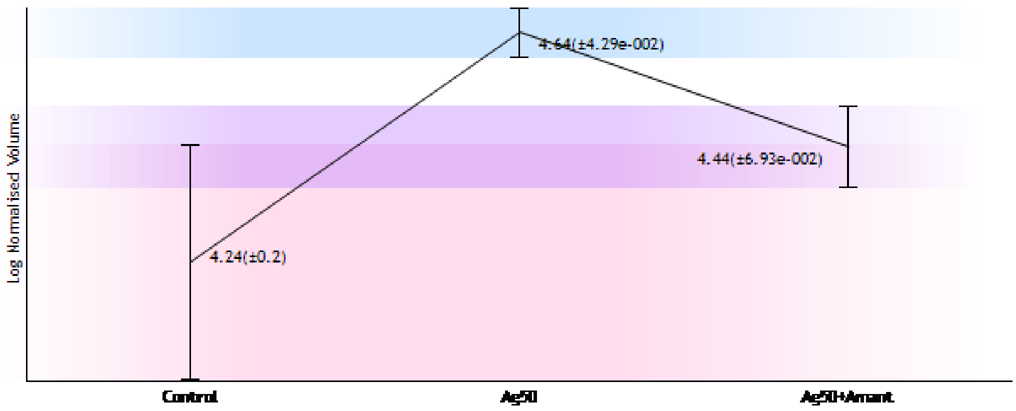

Identifier 296

Position (1030, 554)

Notes

- Anova p-value ≤ 0.05
- Max fold change ≥ 1.5

Control	Ag50	Ag50+Amant
Com Scan samples 1FTSC	Com Scan samples 2 FTSC	Com Scan samples 29 FTSC

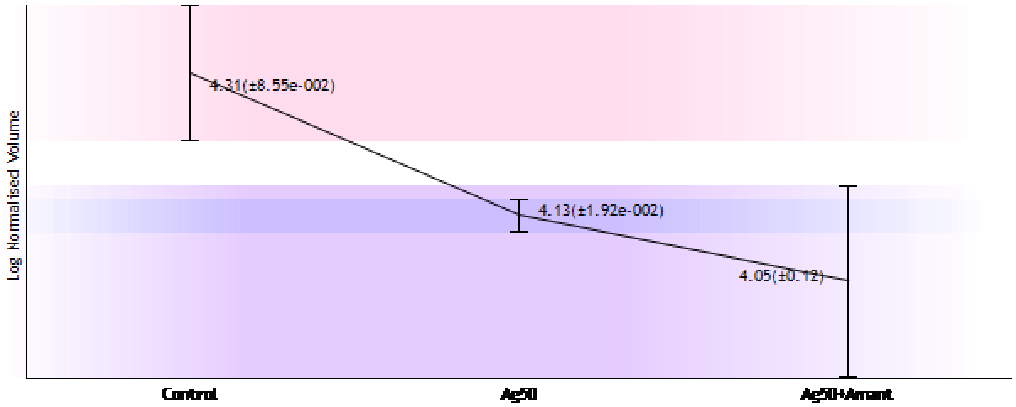

Identifier 123

Position (552, 301)

Notes

- Anova p-value ≤ 0.05
- Max fold change ≥ 1.5

Control	Ag50	Ag50+Amant
Com Scan samples 1FTSC	Com Scan samples 2 FTSC	Com Scan samples 29 FTSC

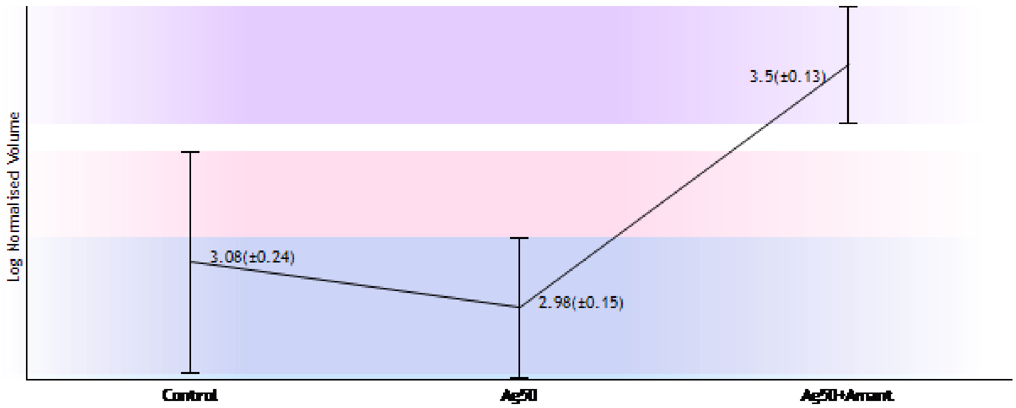

Identifier 297

Position (905, 556)

Notes

- Anova p-value ≤ 0.05
- Max fold change ≥ 1.5

Control	Ag50	Ag50+Amant
Com Scan samples 1FTSC	Com Scan samples 2 FTSC	Com Scan samples 29 FTSC

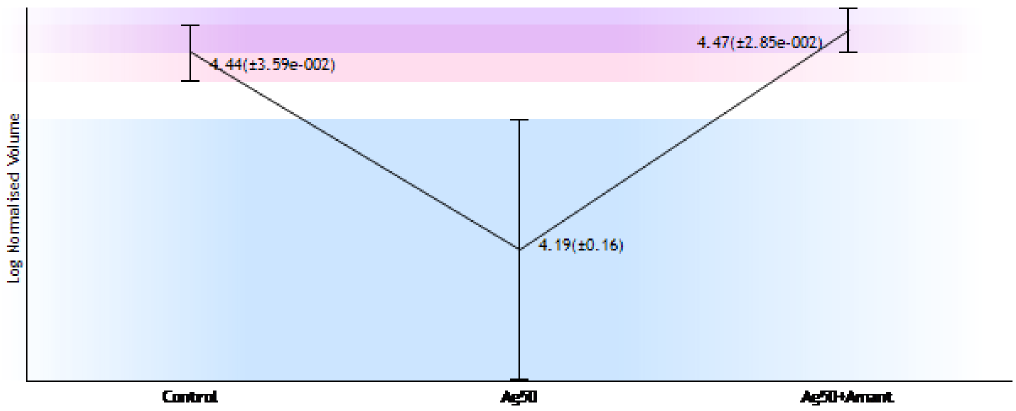

Identifier 125

Position (598, 301)

Notes

- Anova p-value ≤ 0.05
- Max fold change ≥ 1.5

Control	Ag50	Ag50+Amant
Com Scan samples 1FTSC	Com Scan samples 2 FTSC	Com Scan samples 29 FTSC

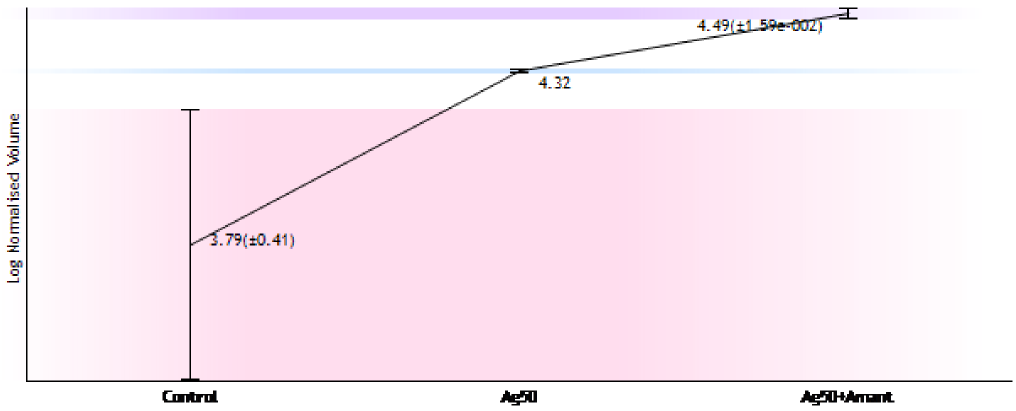

Identifier 52

Position (956, 222)

Notes

- Anova p-value ≤ 0.05
- Max fold change ≥ 1.5

Control	Ag50	Ag50+Amant
Com Scan samples 1FTSC	Com Scan samples 2 FTSC	Com Scan samples 29 FTSC

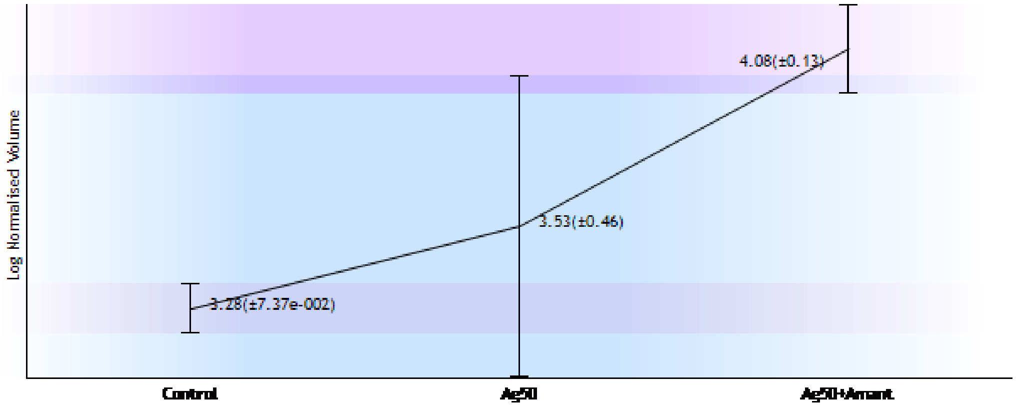

Identifier 201

Position (1083, 404)

Notes

- Anova p-value ≤ 0.05
- Max fold change ≥ 1.5

Control	Ag50	Ag50+Amant
Com Scan samples 1FTSC	Com Scan samples 2 FTSC	Com Scan samples 29 FTSC

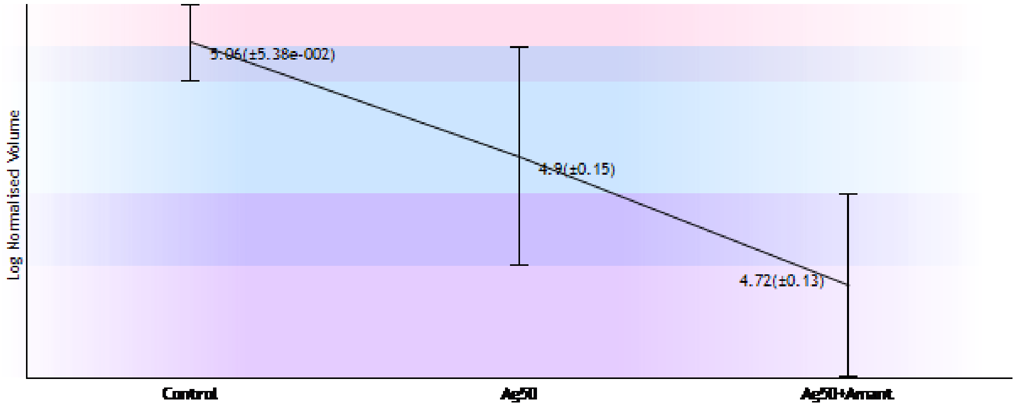

Identifier 434

Position (581, 856)

Notes

- Anova p-value ≤ 0.05
- Max fold change ≥ 1.5

Control	Ag50	Ag50+Amant
Com Scan samples 1FTSC	Com Scan samples 2 FTSC	Com Scan samples 29 FTSC

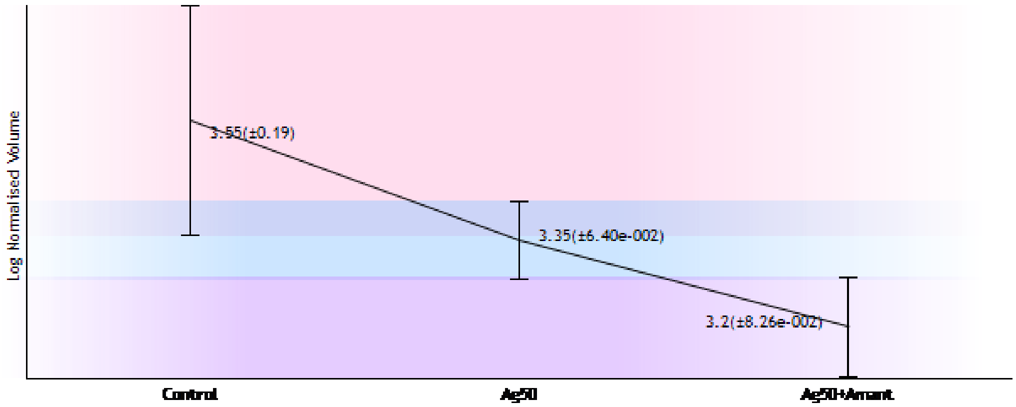

Identifier 313

Position (939, 592)

Notes

- Anova p-value ≤ 0.05
- Max fold change ≥ 1.5

Control	Ag50	Ag50+Amant
Com Scan samples 1FTSC	Com Scan samples 2 FTSC	Com Scan samples 29 FTSC

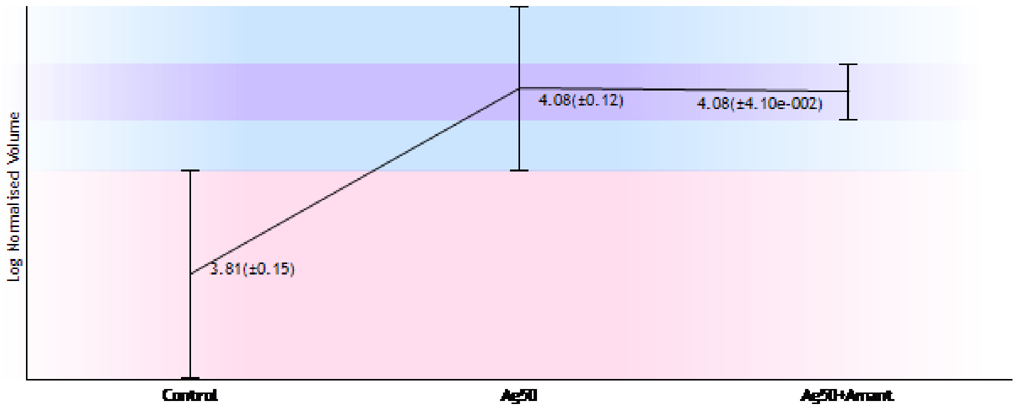

Identifier 275

Position (982, 502)

Notes

- Anova p-value ≤ 0.05
- Max fold change ≥ 1.5

Control	Ag50	Ag50+Amant
Com Scan samples 1FTSC	Com Scan samples 2 FTSC	Com Scan samples 29 FTSC

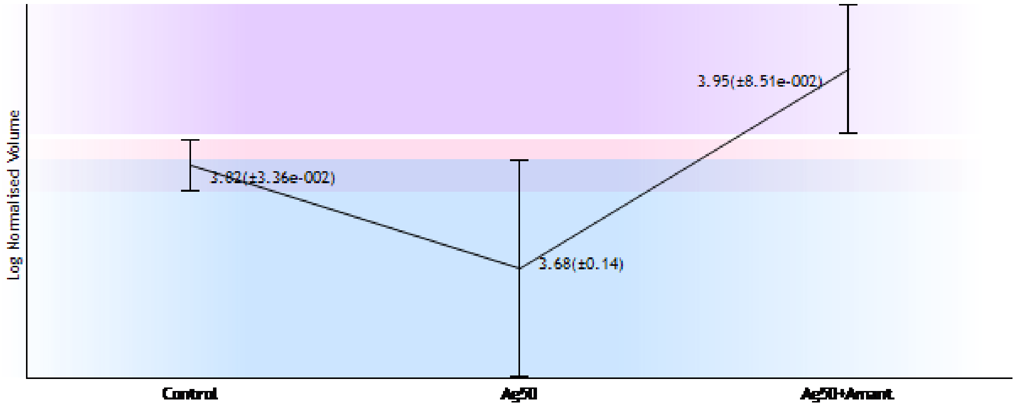

Identifier 230

Position (1201, 436)

Notes

- Anova p-value ≤ 0.05
- Max fold change ≥ 1.5

Control	Ag50	Ag50+Amant
Com Scan samples 1FTSC	Com Scan samples 2 FTSC	Com Scan samples 29 FTSC

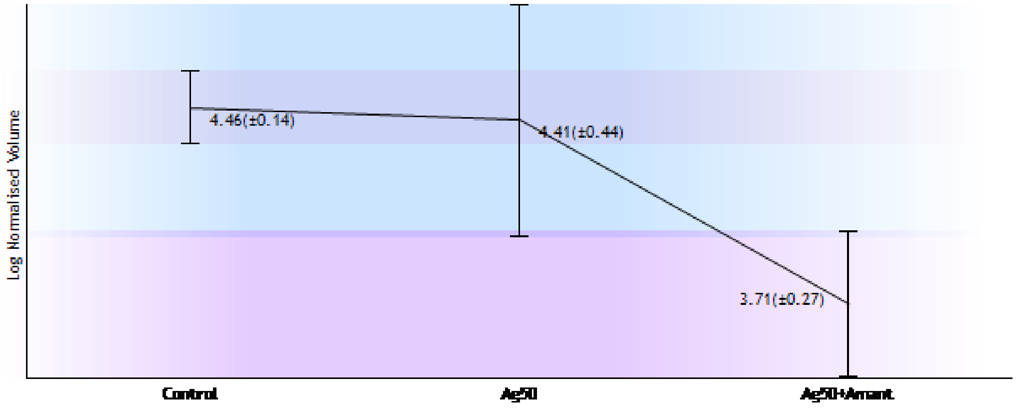

Identifier 210

Position (1269, 419)

Notes

- Anova p-value ≤ 0.05
- Max fold change ≥ 1.5

Control	Ag50	Ag50+Amant
Com Scan samples 1FTSC	Com Scan samples 2 FTSC	Com Scan samples 29 FTSC

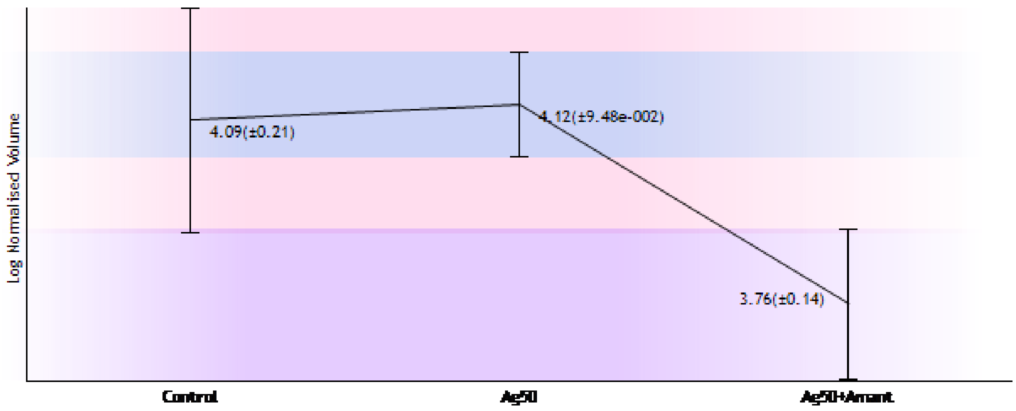

Identifier 432

Position (542, 852)

Notes

- Anova p-value ≤ 0.05
- Max fold change ≥ 1.5

Control	Ag50	Ag50+Amant
Com Scan samples 1FTSC	Com Scan samples 2 FTSC	Com Scan samples 29 FTSC

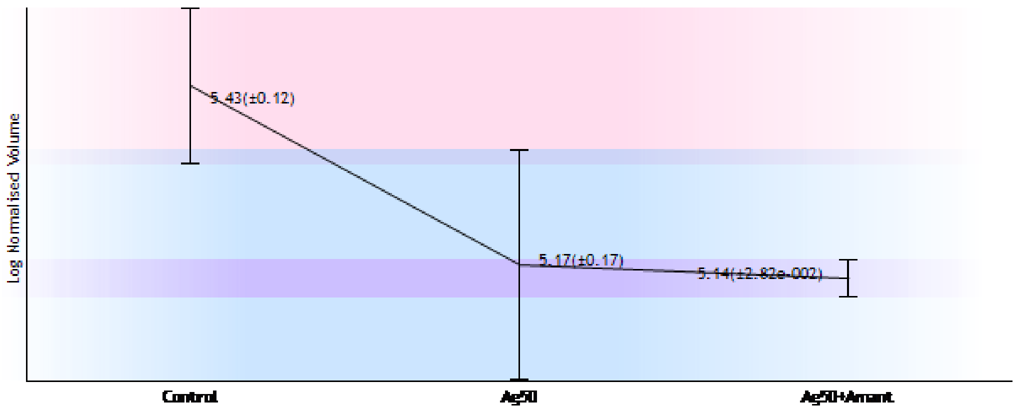

Identifier 133

Position (563, 312)

Notes

- Anova p-value ≤ 0.05
- Max fold change ≥ 1.5

Control	Ag50	Ag50+Amant
Com Scan samples 1FTSC	Com Scan samples 2 FTSC	Com Scan samples 29 FTSC

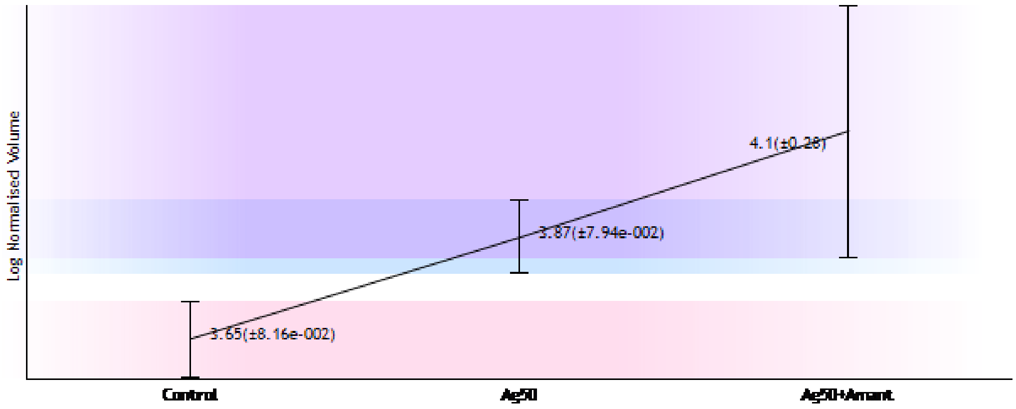

Identifier 396

Position (633, 771)

Notes

- Anova p-value ≤ 0.05
- Max fold change ≥ 1.5

Control	Ag50	Ag50+Amant
Com Scan samples 1FTSC	Com Scan samples 2 FTSC	Com Scan samples 29 FTSC

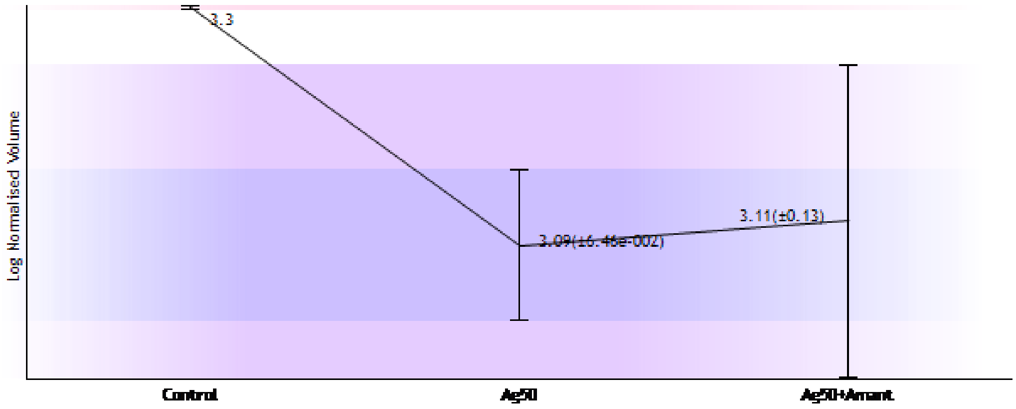

Identifier 436

Position (901, 856)

Notes

- Anova p-value ≤ 0.05
- Max fold change ≥ 1.5

Control	Ag50	Ag50+Amant
Com Scan samples 1FTSC	Com Scan samples 2 FTSC	Com Scan samples 29 FTSC

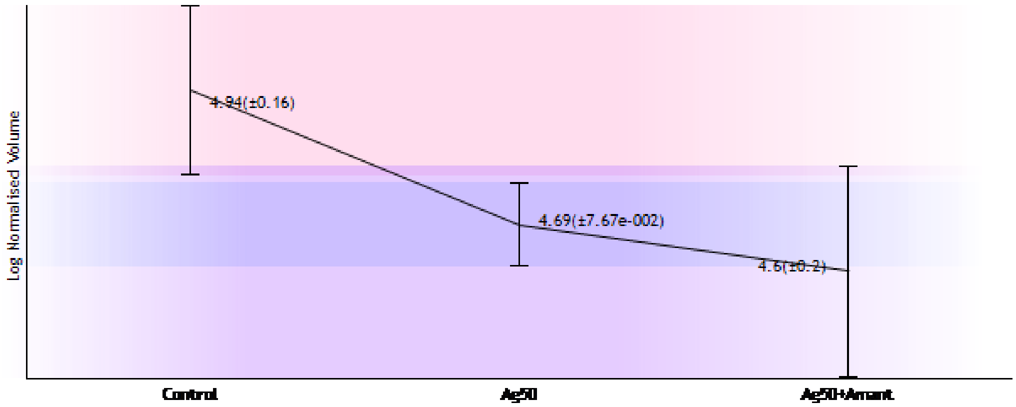

Identifier 77

Position (771, 247)

Notes

- Anova p-value ≤ 0.05
- Max fold change ≥ 1.5

Control	Ag50	Ag50+Amant
Com Scan samples 1FTSC	Com Scan samples 2 FTSC	Com Scan samples 29 FTSC

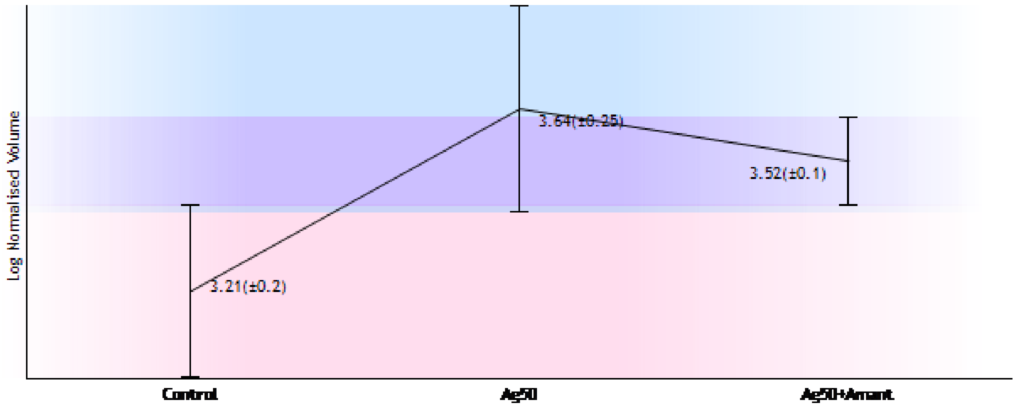

Identifier 124

Position (580, 301)

Notes

- Anova p-value ≤ 0.05
- Max fold change ≥ 1.5

Control	Ag50	Ag50+Amant
Com Scan samples 1FTSC	Com Scan samples 2 FTSC	Com Scan samples 29 FTSC

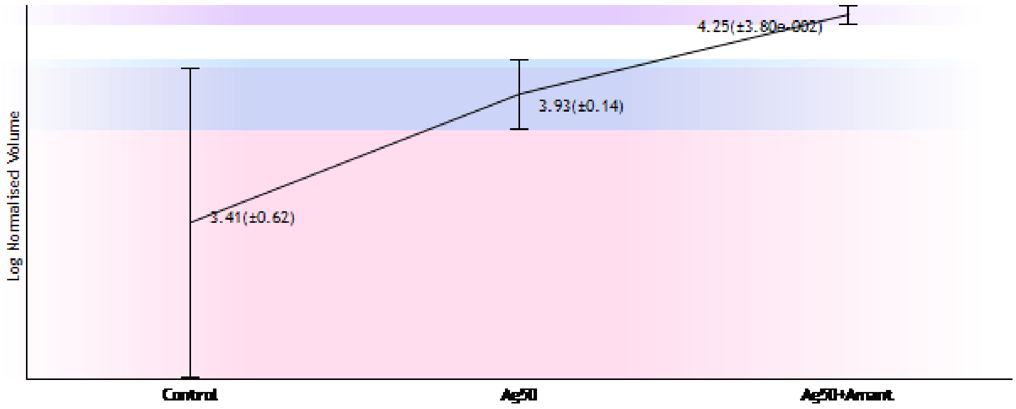

Identifier 72

Position (931, 238)

Notes

- Anova p-value ≤ 0.05
- Max fold change ≥ 1.5

Control	Ag50	Ag50+Amant
Com Scan samples 1FTSC	Com Scan samples 2 FTSC	Com Scan samples 29 FTSC

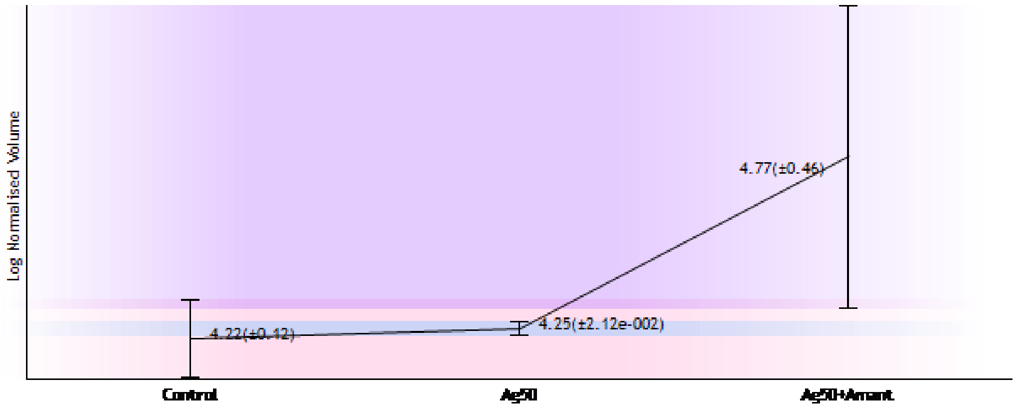

Identifier 395

Position (657, 770)

Notes

- Anova p-value ≤ 0.05
- Max fold change ≥ 1.5

Control	Ag50	Ag50+Amant
Com Scan samples 1FTSC	Com Scan samples 2 FTSC	Com Scan samples 29 FTSC

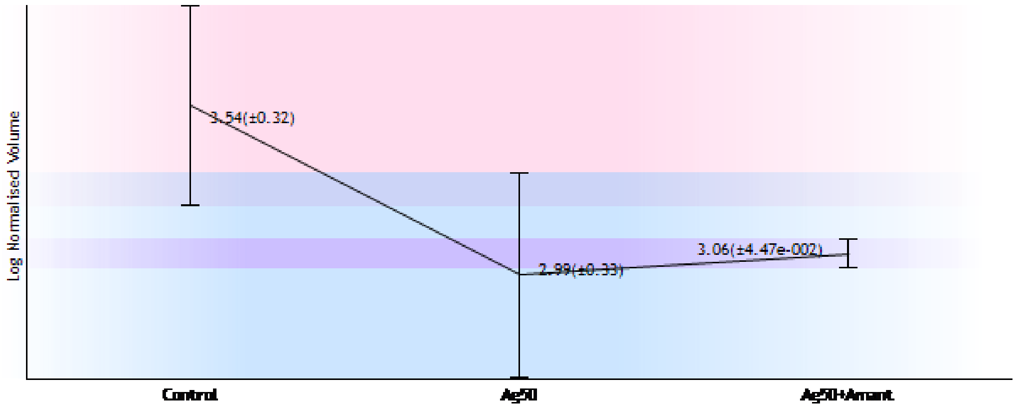

Identifier 207

Position (986, 409)

Notes

- Anova p-value ≤ 0.05
- Max fold change ≥ 1.5

Control	Ag50	Ag50+Amant
Com Scan samples 1FTSC	Com Scan samples 2 FTSC	Com Scan samples 29 FTSC

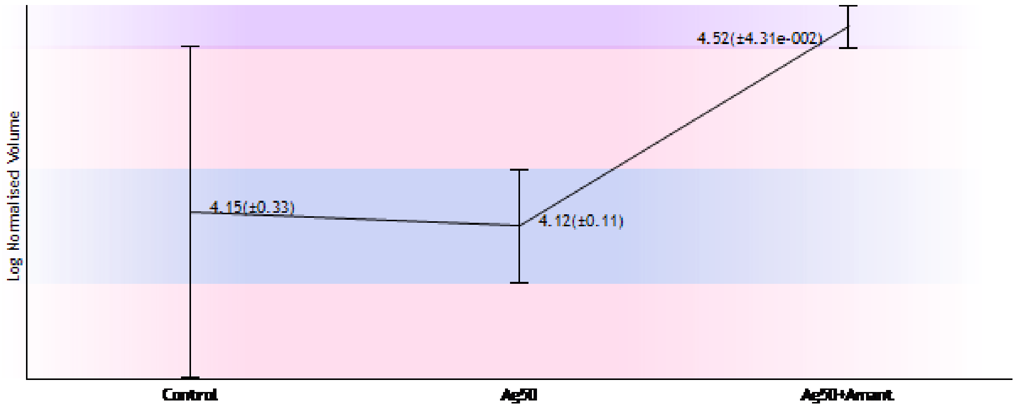

Identifier 135

Position (1010, 318)

Notes

- Anova p-value ≤ 0.05
- Max fold change ≥ 1.5

Control	Ag50	Ag50+Amant
Com Scan samples 1FTSC	Com Scan samples 2 FTSC	Com Scan samples 29 FTSC

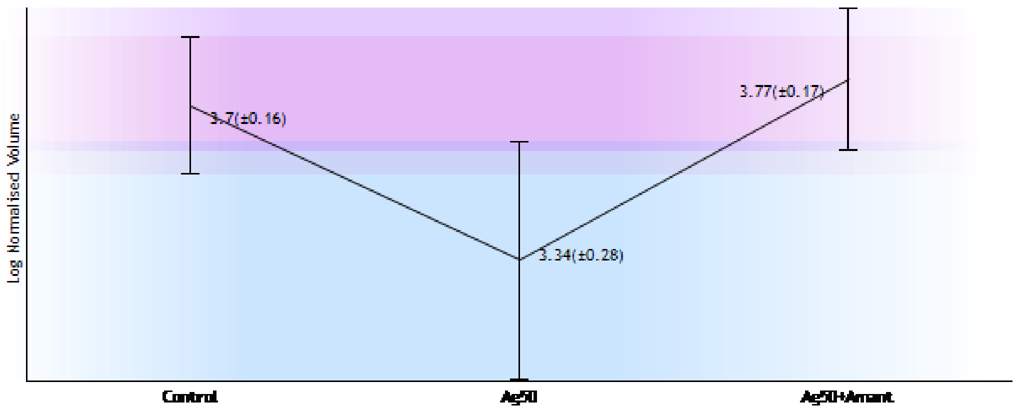

Identifier 331

Position (785, 625)

Notes

- Anova p-value ≤ 0.05
- Max fold change ≥ 1.5

Control	Ag50	Ag50+Amant
Com Scan samples 1FTSC	Com Scan samples 2 FTSC	Com Scan samples 29 FTSC

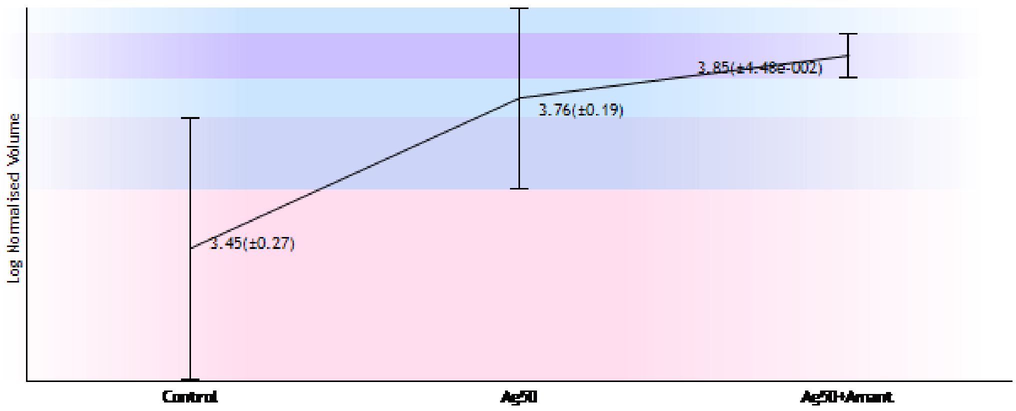

Identifier 358

Position (900, 665)

Notes

- Anova p-value ≤ 0.05
- Max fold change ≥ 1.5

Control	Ag50	Ag50+Amant
Com Scan samples 1FTSC	Com Scan samples 2 FTSC	Com Scan samples 29 FTSC

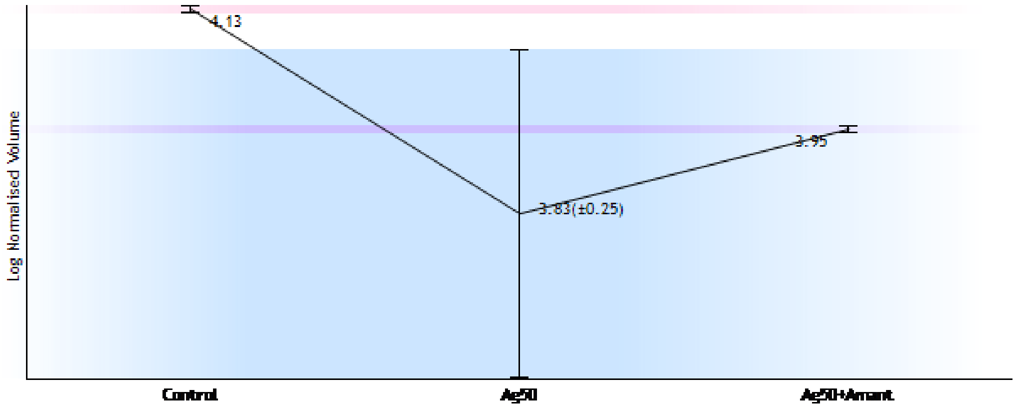

Identifier 378

Position (740, 719)

Notes

- Anova p-value ≤ 0.05
- Max fold change ≥ 1.5

Control	Ag50	Ag50+Amant
Com Scan samples 1FTSC	Com Scan samples 2 FTSC	Com Scan samples 29 FTSC

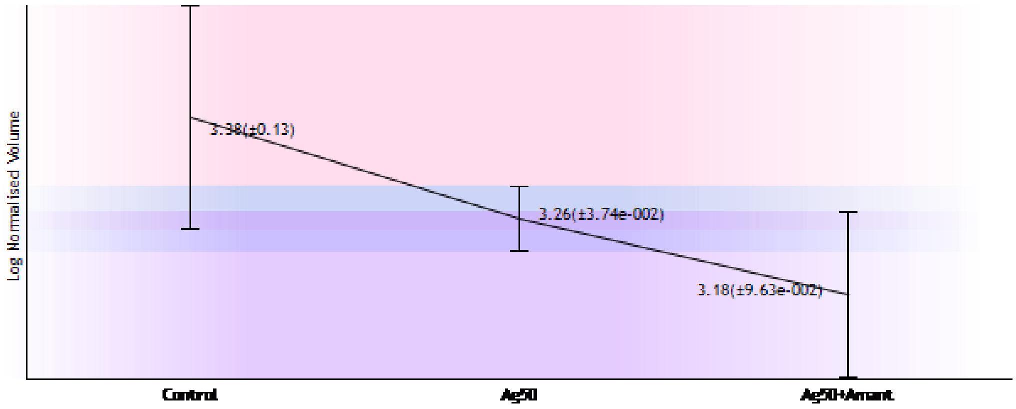

Identifier 374

Position (714, 694)

Notes

- Anova p-value ≤ 0.05
- Max fold change ≥ 1.5

Control	Ag50	Ag50+Amant
Com Scan samples 1FTSC	Com Scan samples 2 FTSC	Com Scan samples 29 FTSC

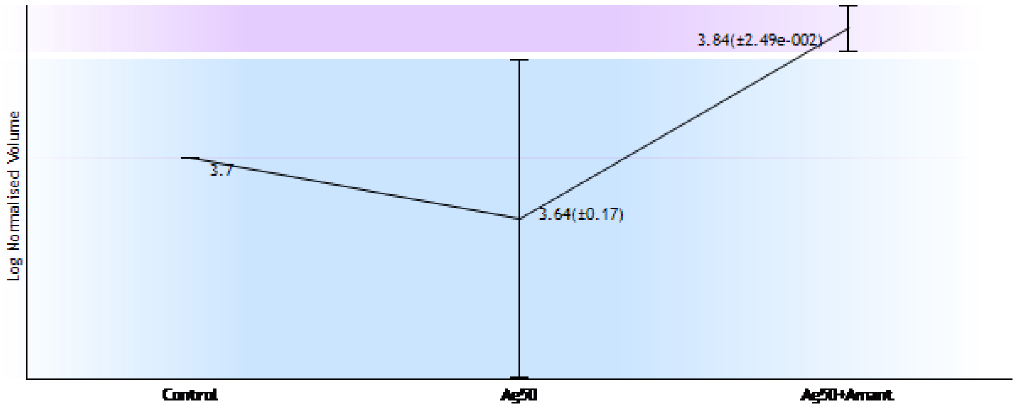

Identifier 43

Position (933, 208)

Notes

- Anova p-value ≤ 0.05
- Max fold change ≥ 1.5

Control	Ag50	Ag50+Amant
Com Scan samples 1FTSC	Com Scan samples 2 FTSC	Com Scan samples 29 FTSC

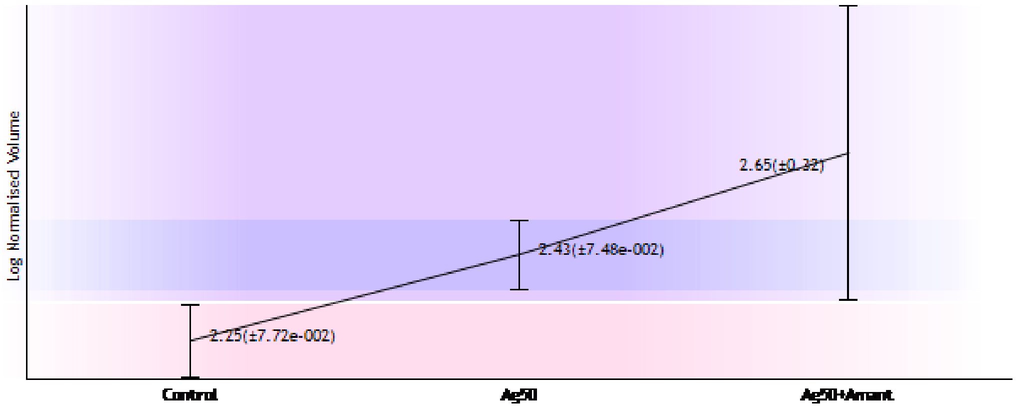

Identifier 81

Position (617, 253)

Notes

- Anova p-value ≤ 0.05
- Max fold change ≥ 1.5

Control	Ag50	Ag50+Amant
Com Scan samples 1FTSC	Com Scan samples 2 FTSC	Com Scan samples 29 FTSC

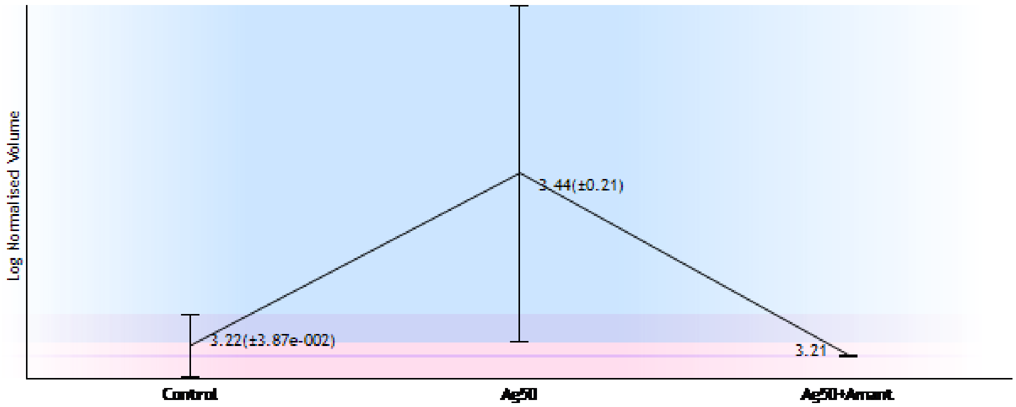

Identifier 128

Position (713, 303)

Notes

- Anova p-value ≤ 0.05
- Max fold change ≥ 1.5

Control	Ag50	Ag50+Amant
Com Scan samples 1FTSC	Com Scan samples 2 FTSC	Com Scan samples 29 FTSC

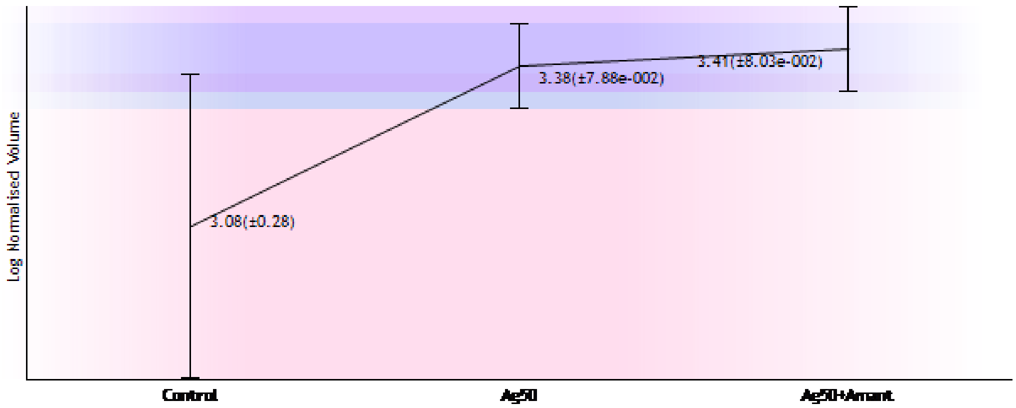

Younes Comm IAF Gills

Experiment: Younes Comm IAF Gills

Report created: 22/12/2015 12:30:21

Reference image

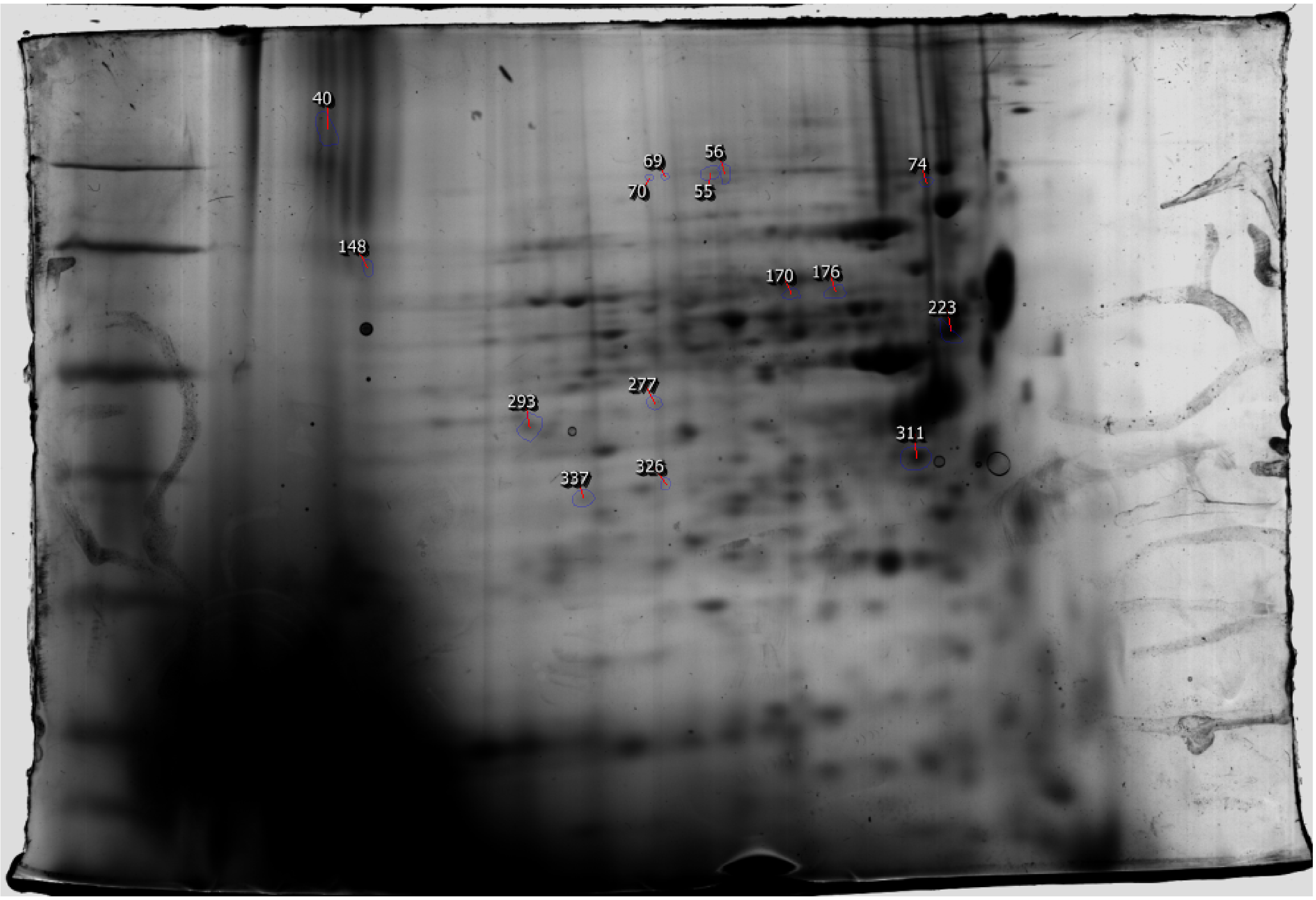

Experiment Design

Condition	Control	Ag50	Ag50+Amant
Replicates	2	2	2

Spots

#	Anova (p)	Fold	Tags	Notes	pI	MW	Protein Accession	Protein Description	Protein pI	Protein MW	Protein URL	Average Normalised Volumes		
												Control	Ag50	Ag50+Amant
55	8.787e-005	3.7										2796.984	5484.644	1480.067
70	0.004	4.3										324.923	697.084	163.253
148	0.005	2.8										2114.146	761.499	830.908
69	0.006	7.1										323.283	800.612	113.105
326	0.012	3.1										615.344	587.220	1829.790
223	0.018	2.4										1.323e+004	6297.628	5411.294
293	0.019	2.0										8735.312	1.767e+004	9116.154
277	0.023	2.1										2811.259	1368.490	2699.291
311	0.032	2.8										1.709e+004	1.842e+004	6584.466

#	Anova (p)	Fold	Tags	Notes	pI	MW	Protein Accession	Protein Description	Protein pI	Protein MW	Protein URL	Average Normalised Volumes		
												Control	Ag50	Ag50+Amant
74	0.033	2.1										2280.681	1235.220	1101.959
40	0.034	4.6										1.142e+004	2983.302	1.367e+004
176	0.038	2.4										2876.156	5036.038	6948.139
170	0.042	2.0										1875.842	3815.528	2598.739
337	0.044	2.1										4508.486	8150.887	3855.440
56	0.049	4.8										2052.164	5210.821	1093.909

Tags	
	Anova p-value ≤ 0.05
	Max fold change ≥ 2

Identifier 55

Position (855, 200)

Notes

- Anova p-value ≤ 0.05
- Max fold change ≥ 2

Control	Ag50	Ag50+Amant
Com Scan samples 1IAF	Com Scan samples 2 IAF	Com Scan samples 29 IAF

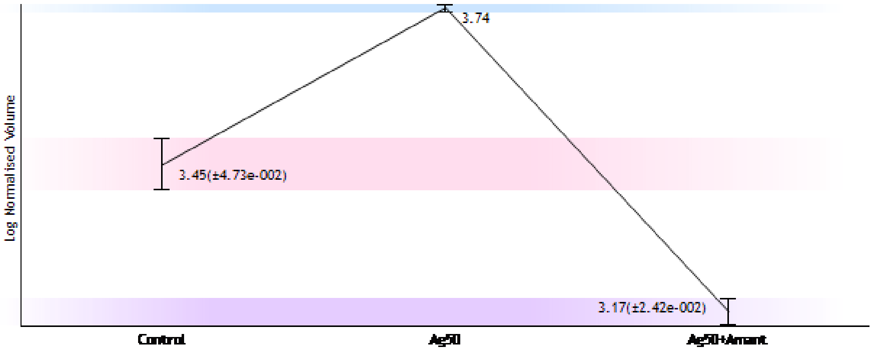

Identifier 70

Position (774, 206)

Notes

- Anova p-value ≤ 0.05
- Max fold change ≥ 2

Control	Ag50	Ag50+Amant
Com Scan samples 1IAF	Com Scan samples 2 IAF	Com Scan samples 29 IAF

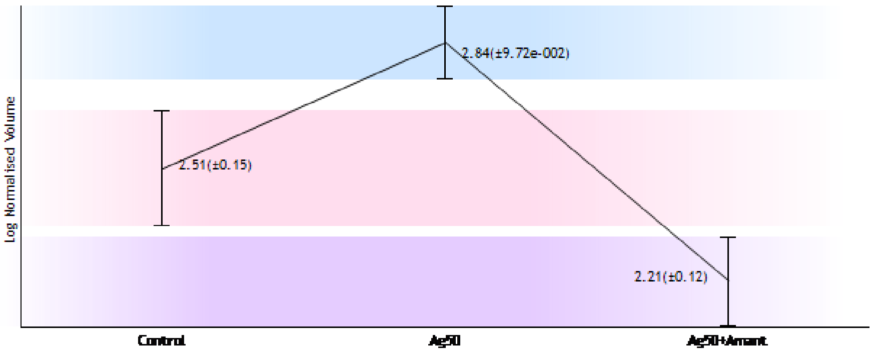

Identifier 148

Position (438, 308)

Notes

- Anova p-value ≤ 0.05
- Max fold change ≥ 2

Control	Ag50	Ag50+Amant
Com Scan samples 1IAF	Com Scan samples 2 IAF	Com Scan samples 29 IAF

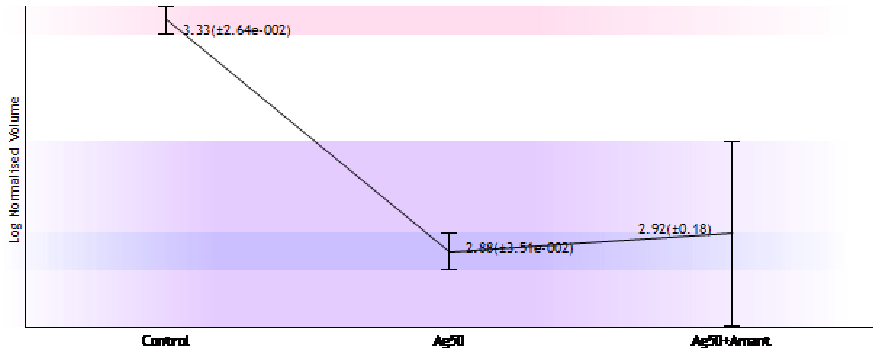

Identifier 69

Position (792, 205)

Notes

- Anova p-value ≤ 0.05
- Max fold change ≥ 2

Control	Ag50	Ag50+Amant
Com Scan samples 1IAF	Com Scan samples 2 IAF	Com Scan samples 29 IAF

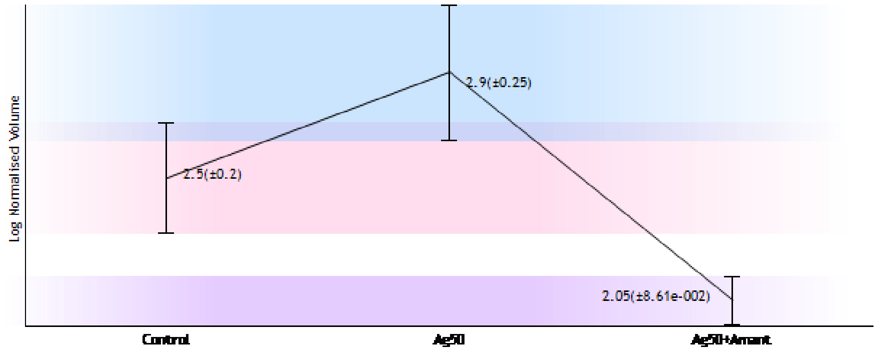

Identifier 326

Position (795, 574)

Notes

- Anova p-value ≤ 0.05
- Max fold change ≥ 2

Control	Ag50	Ag50+Amant
Com Scan samples 1IAF	Com Scan samples 2 IAF	Com Scan samples 29 IAF

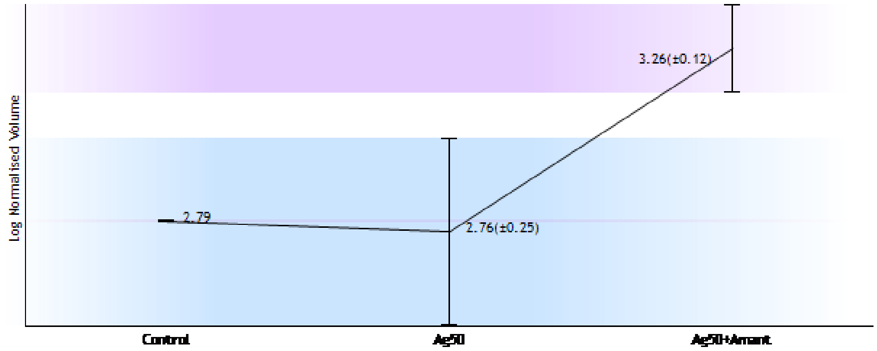

Identifier 223

Position (1134, 391)

Notes

- Anova p-value ≤ 0.05
- Max fold change ≥ 2

Control	Ag50	Ag50+Amant
Com Scan samples 1IAF	Com Scan samples 2 IAF	Com Scan samples 29 IAF

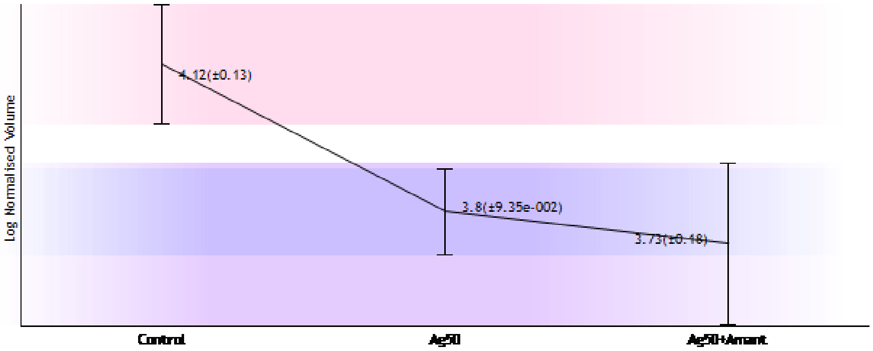

Identifier 293

Position (632, 505)

Notes

- Anova p-value ≤ 0.05
- Max fold change ≥ 2

Control	Ag50	Ag50+Amant
Com Scan samples 1IAF	Com Scan samples 2 IAF	Com Scan samples 29 IAF

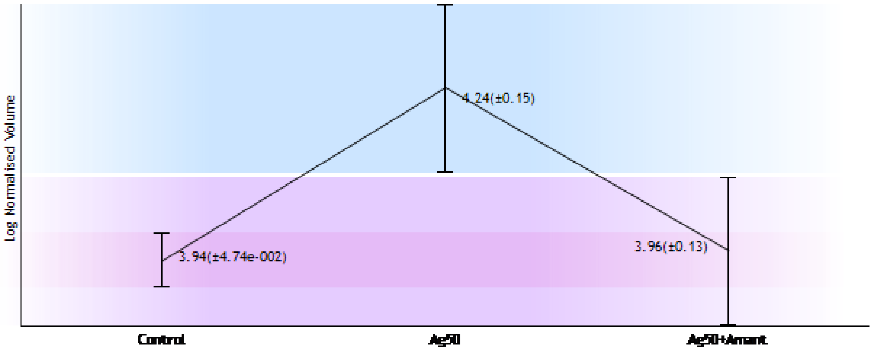

Identifier 277

Position (782, 476)

Notes

- Anova p-value ≤ 0.05
- Max fold change ≥ 2

Control	Ag50	Ag50+Amant
Com Scan samples 1IAF	Com Scan samples 2 IAF	Com Scan samples 29 IAF

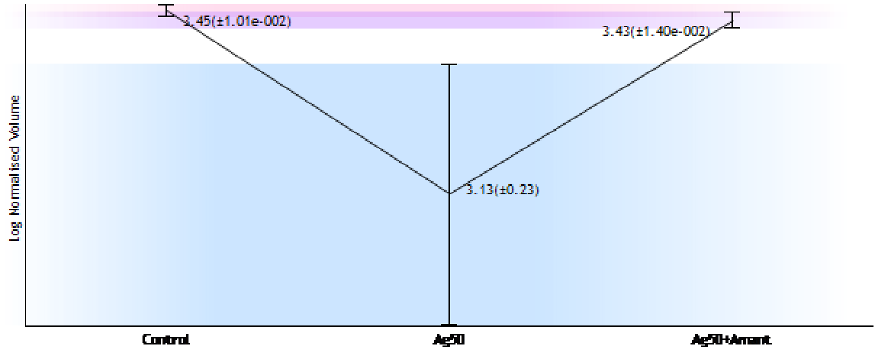

Identifier 311

Position (1093, 541)

Notes

- Anova p-value ≤ 0.05
- Max fold change ≥ 2

Control	Ag50	Ag50+Amant
Com Scan samples 1IAF	Com Scan samples 2 IAF	Com Scan samples 29 IAF

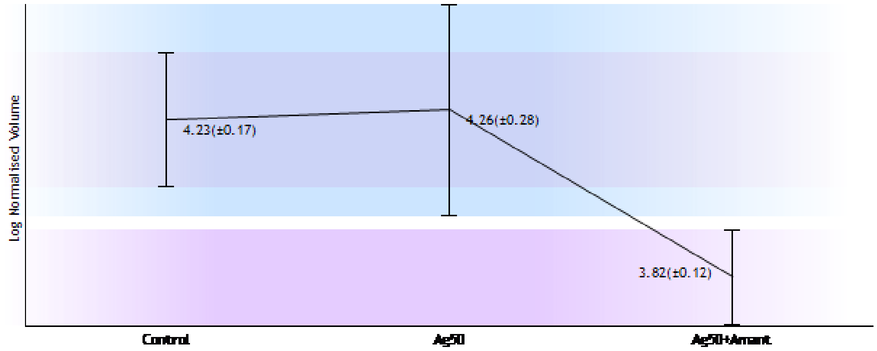

Identifier 74

Position (1107, 210)

Notes

- Anova p-value ≤ 0.05
- Max fold change ≥ 2

Control	Ag50	Ag50+Amant
Com Scan samples 1IAF	Com Scan samples 2 IAF	Com Scan samples 29 IAF

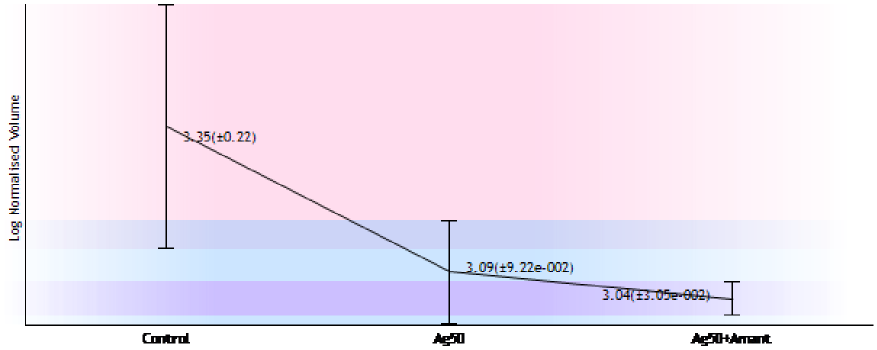

Identifier 40

Position (393, 168)

Notes

- Anova p-value ≤ 0.05

Max fold change ≥ 2

Control	Ag50	Ag50+Amant
Com Scan samples 1IAF	Com Scan samples 2 IAF	Com Scan samples 29 IAF

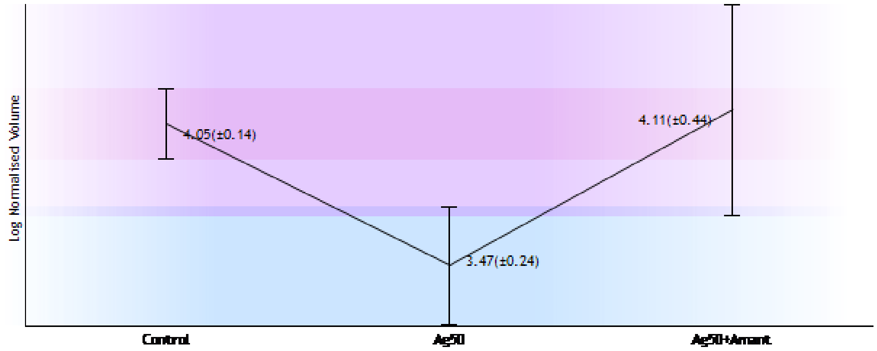

Identifier 176

Position (1000, 344)

Notes

Anova p-value ≤ 0.05

Max fold change ≥ 2

Control	Ag50	Ag50+Amant
Com Scan samples 1IAF	Com Scan samples 2 IAF	Com Scan samples 29 IAF

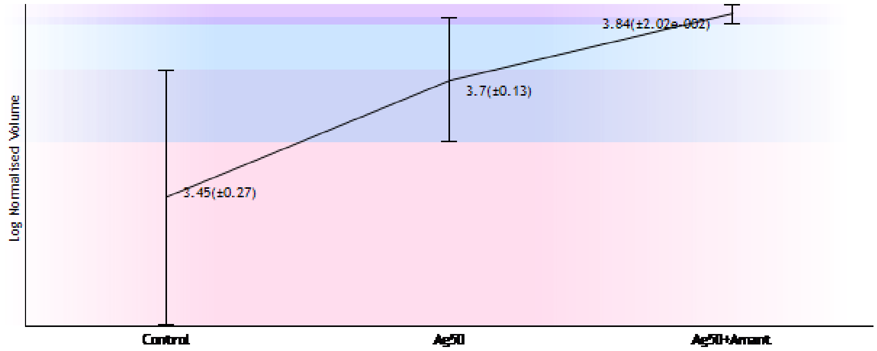

Identifier 170

Position (948, 339)

Notes

Anova p-value ≤ 0.05

Max fold change ≥ 2

Control	Ag50	Ag50+Amant
Com Scan samples 1IAF	Com Scan samples 2 IAF	Com Scan samples 29 IAF

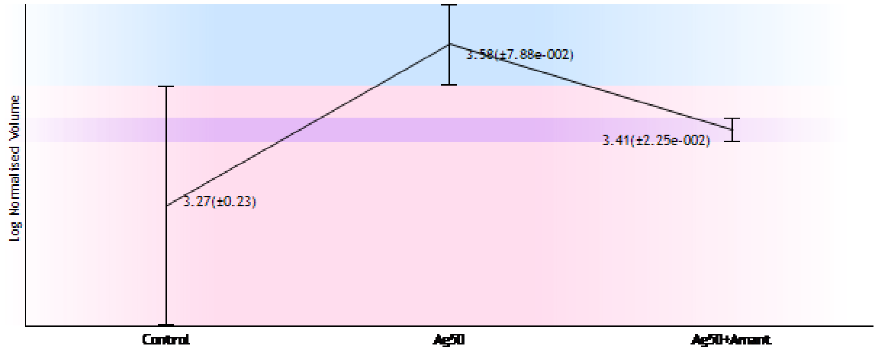

Identifier 337

Position (694, 591)

Notes

- Anova p-value ≤ 0.05
- Max fold change ≥ 2

Control	Ag50	Ag50+Amant
Com Scan samples 1IAF	Com Scan samples 2 IAF	Com Scan samples 29 IAF

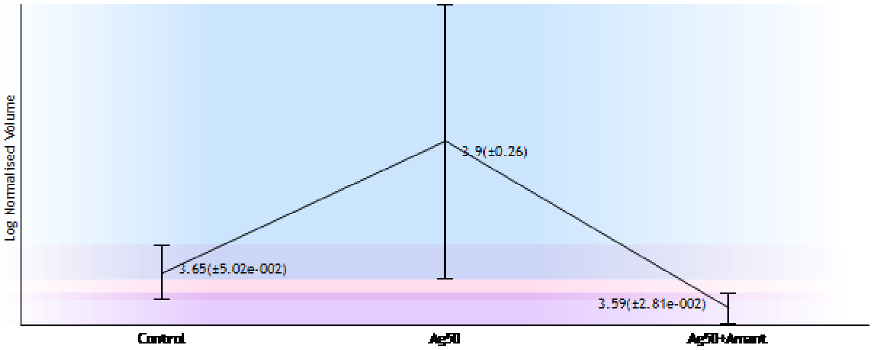

Identifier 56

Position (869, 200)

Notes

- Anova p-value ≤ 0.05
- Max fold change ≥ 2

Control	Ag50	Ag50+Amant
Com Scan samples 1IAF	Com Scan samples 2 IAF	Com Scan samples 29 IAF

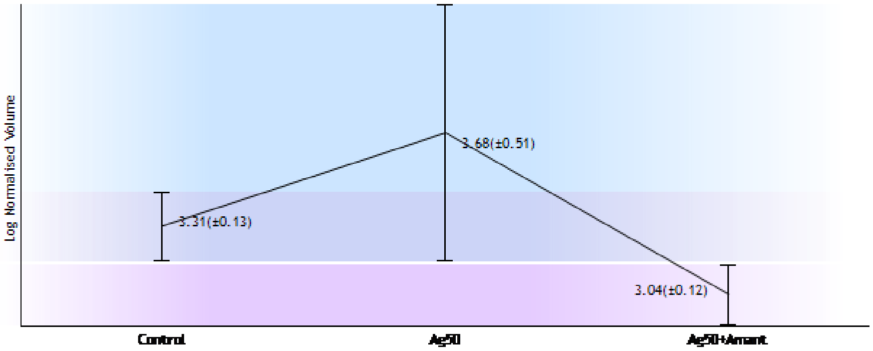

Younes FTSC Digestive gland

Experiment: Younes FTSC Digestive gland

Report created: 22/12/2015 10:48:10

Reference image

Experiment Design

Condition	Control	Ag50	Ag50+Amant
Replicates	2	2	2

Spots

#	Anova (p)	Fold	Tags	Notes	pI	MW	Protein Accession	Protein Description	Protein pI	Protein MW	Protein URL	Average Normalised Volumes		
												Control	Ag50	Ag50+Amant
11	0.137	2.1										4026.799	8614.313	7978.368
26	0.145	2.4										1873.272	1173.456	795.234
19	0.150	3.3										147.526	481.206	256.560
6	0.159	1.7										7294.406	1.213e+004	9982.232
17	0.178	2.6										9.159e+004	3.591e+004	5.382e+004
22	0.290	2.3										1.473e+004	3.453e+004	2.207e+004
10	0.342	2.1										400.272	398.003	848.117
30	0.411	1.6										3477.376	5664.588	4454.946
4	0.413	2.6										939.204	503.367	357.389
28	0.437	2.6										2345.395	3381.674	1306.578
20	0.443	1.8										2112.376	1981.747	1191.613
35	0.464	2.9										566.500	1325.192	1666.198
24	0.468	3.4										1727.812	1722.891	506.294
36	0.475	1.9										5614.573	8453.973	1.052e+004
29	0.493	2.3										7078.727	3025.106	4160.862
9	0.544	1.6										1.811e+004	1.149e+004	1.715e+004
18	0.565	1.8										1.720e+004	3.139e+004	2.877e+004
16	0.570	2.4										5041.957	9935.259	4080.628
33	0.571	2.6										3.664e+004	5.088e+004	9.534e+004
34	0.587	1.9										1.235e+004	1.293e+004	2.366e+004
15	0.599	2.5										5866.409	2375.280	3302.086
25	0.604	2.3										1.931e+005	2.465e+005	4.363e+005
7	0.604	1.9										3.564e+004	4.462e+004	2.372e+004
32	0.605	1.8										4.450e+005	4.876e+005	8.000e+005
3	0.612	2.8										1818.046	2561.646	905.598
13	0.626	3.2										9264.841	1.852e+004	5741.305
27	0.638	2.2										276.401	505.179	233.850
2	0.646	1.4										1.143e+004	7896.040	7980.950
12	0.694	2.0										3690.422	3027.116	1869.107
31	0.714	2.0										9.800e+004	1.445e+005	1.996e+005
23	0.794	2.1										4492.504	2122.489	2654.890
14	0.794	1.7										8994.921	5168.991	7208.470

#	Anova (p)	Fold	Tags	Notes	pI	MW	Protein Accession	Protein Description	Protein pI	Protein MW	Protein URL	Average Normalised Volumes		
												Control	Ag50	Ag50+Amant
1	0.858	2.1										1783.590	3666.995	2071.367
5	0.896	1.4										891.614	796.468	1106.987
8	0.923	1.3										687.520	837.896	648.304
21	0.930	2.5										978.806	1667.996	672.544

Tags	
	Anova p-value ≤ 0.05
	Max fold change ≥ 2
	Max fold change ≥ 1.5
	Anova p-value ≤ 0.1

Identifier 11

Position (442, 203)

Notes

Control	Ag50	Ag50+Amant
2DG Samples 37 FTSC	2DG Samples 38 FTSC	2DG Samples 65 FTSC

Identifier 26

Position (286, 420)

Notes

Control	Ag50	Ag50+Amant
2DG Samples 37 FTSC	2DG Samples 38 FTSC	2DG Samples 65 FTSC

Identifier 19

Position (633, 296)

Notes

Control	Ag50	Ag50+Amant
2DG Samples 37 FTSC	2DG Samples 38 FTSC	2DG Samples 65 FTSC

Identifier 6

Position (422, 141)

Notes

Control	Ag50	Ag50+Amant
2DG Samples 37 FTSC	2DG Samples 38 FTSC	2DG Samples 65 FTSC

Identifier 17

Position (243, 268)

Notes

Control	Ag50	Ag50+Amant
2DG Samples 37 FTSC	2DG Samples 38 FTSC	2DG Samples 65 FTSC

Identifier 22

Position (441, 310)

Notes

Control	Ag50	Ag50+Amant
2DG Samples 37 FTSC	2DG Samples 38 FTSC	2DG Samples 65 FTSC

Identifier 10

Position (115, 200)

Notes

Control	Ag50	Ag50+Amant
2DG Samples 37 FTSC	2DG Samples 38 FTSC	2DG Samples 65 FTSC

Identifier 30

Position (642, 456)

Notes

Control	Ag50	Ag50+Amant
2DG Samples 37 FTSC	2DG Samples 38 FTSC	2DG Samples 65 FTSC

Identifier 4

Position (536, 129)

Notes

Control	Ag50	Ag50+Amant
2DG Samples 37 FTSC	2DG Samples 38 FTSC	2DG Samples 65 FTSC

Identifier 28

Position (350, 425)

Notes

Control	Ag50	Ag50+Amant
2DG Samples 37 FTSC	2DG Samples 38 FTSC	2DG Samples 65 FTSC

Identifier 20

Position (172, 299)

Notes

Control	Ag50	Ag50+Amant
2DG Samples 37 FTSC	2DG Samples 38 FTSC	2DG Samples 65 FTSC

Identifier 35

Position (255, 522)

Notes

Control	Ag50	Ag50+Amant
2DG Samples 37 FTSC	2DG Samples 38 FTSC	2DG Samples 65 FTSC

Identifier 24

Position (439, 363)

Notes

Control	Ag50	Ag50+Amant
2DG Samples 37 FTSC	2DG Samples 38 FTSC	2DG Samples 65 FTSC

Identifier 36

Position (216, 536)

Notes

Control	Ag50	Ag50+Amant
2DG Samples 37 FTSC	2DG Samples 38 FTSC	2DG Samples 65 FTSC

Identifier 29

Position (265, 437)

Notes

Control	Ag50	Ag50+Amant
2DG Samples 37 FTSC	2DG Samples 38 FTSC	2DG Samples 65 FTSC

Identifier 9

Position (535, 176)

Notes

Control	Ag50	Ag50+Amant
2DG Samples 37 FTSC	2DG Samples 38 FTSC	2DG Samples 65 FTSC

Identifier 18

Position (271, 273)
Notes

Control	Ag50	Ag50+Amant
2DG Samples 37 FTSC	2DG Samples 38 FTSC	2DG Samples 65 FTSC

Identifier 16

Position (632, 261)
Notes

Control	Ag50	Ag50+Amant
2DG Samples 37 FTSC	2DG Samples 38 FTSC	2DG Samples 65 FTSC

Identifier 33

Position (400, 475)
Notes

Control	Ag50	Ag50+Amant
2DG Samples 37 FTSC	2DG Samples 38 FTSC	2DG Samples 65 FTSC

Identifier 34

Position (436, 476)
Notes

Control	Ag50	Ag50+Amant
2DG Samples 37 FTSC	2DG Samples 38 FTSC	2DG Samples 65 FTSC

Identifier 15

Position (543, 259)
Notes

Control	Ag50	Ag50+Amant
2DG Samples 37 FTSC	2DG Samples 38 FTSC	2DG Samples 65 FTSC

Identifier 25

Position (436, 416)
Notes

Control	Ag50	Ag50+Amant
2DG Samples 37 FTSC	2DG Samples 38 FTSC	2DG Samples 65 FTSC

Identifier 7

Position (617, 150)

Notes

Control	Ag50	Ag50+Amant
2DG Samples 37 FTSC	2DG Samples 38 FTSC	2DG Samples 65 FTSC

Identifier 32

Position (552, 466)

Notes

Control	Ag50	Ag50+Amant
2DG Samples 37 FTSC	2DG Samples 38 FTSC	2DG Samples 65 FTSC

Identifier 3

Position (368, 97)

Notes

Control	Ag50	Ag50+Amant
2DG Samples 37 FTSC	2DG Samples 38 FTSC	2DG Samples 65 FTSC

Identifier 13

Position (627, 230)

Notes

Control	Ag50	Ag50+Amant
2DG Samples 37 FTSC	2DG Samples 38 FTSC	2DG Samples 65 FTSC

Identifier 27

Position (306, 422)

Notes

Control	Ag50	Ag50+Amant
2DG Samples 37 FTSC	2DG Samples 38 FTSC	2DG Samples 65 FTSC

Identifier 2

Position (126, 96)

Notes

Control	Ag50	Ag50+Amant
2DG Samples 37 FTSC	2DG Samples 38 FTSC	2DG Samples 65 FTSC

Identifier 12

Position (583, 225)
Notes

Control	Ag50	Ag50+Amant
2DG Samples 37 FTSC	2DG Samples 38 FTSC	2DG Samples 65 FTSC

Identifier 31

Position (357, 465)
Notes

Control	Ag50	Ag50+Amant
2DG Samples 37 FTSC	2DG Samples 38 FTSC	2DG Samples 65 FTSC

Identifier 23

Position (544, 310)
Notes

Control	Ag50	Ag50+Amant
2DG Samples 37 FTSC	2DG Samples 38 FTSC	2DG Samples 65 FTSC

Identifier 14

Position (540, 234)
Notes

Control	Ag50	Ag50+Amant
2DG Samples 37 FTSC	2DG Samples 38 FTSC	2DG Samples 65 FTSC

Identifier 1

Position (610, 94)
Notes

Control	Ag50	Ag50+Amant
2DG Samples 37 FTSC	2DG Samples 38 FTSC	2DG Samples 65 FTSC

Identifier 5

Position (135, 138)

Notes

Control	Ag50	Ag50+Amant
2DG Samples 37 FTSC	2DG Samples 38 FTSC	2DG Samples 65 FTSC

Identifier 8

Position (133, 155)

Notes

Control	Ag50	Ag50+Amant
2DG Samples 37 FTSC	2DG Samples 38 FTSC	2DG Samples 65 FTSC

Identifier 21

Position (416, 309)

Notes

Control	Ag50	Ag50+Amant
2DG Samples 37 FTSC	2DG Samples 38 FTSC	2DG Samples 65 FTSC

Younes FTSC Gills

Experiment: Younes FTSC Gills

Report created: 22/12/2015 11:18:45

Reference image

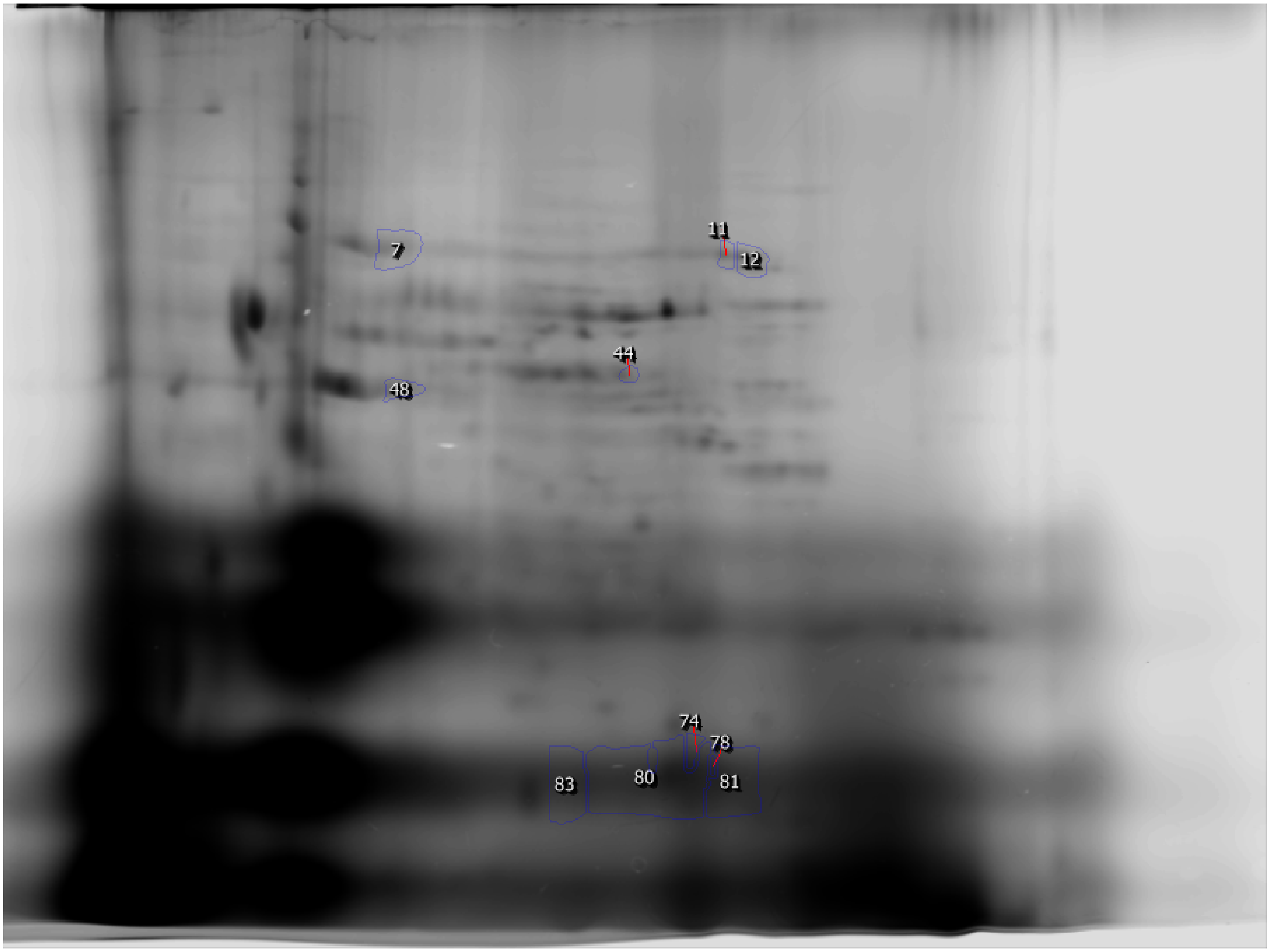

Experiment Design

Condition	Control	Ag50	Ag+Amant
Replicates	2	2	2

Spots

#	Anova (p)	Fold	Tags	Notes	pI	MW	Protein Accession	Protein Description	Protein pI	Protein MW	Protein URL	Average Normalised Volumes		
												Control	Ag50	Ag+Amant
80	0.002	2.7										1.183e+005	8.999e+004	4.365e+004
11	0.004	2.6										1174.056	1925.854	3016.544
7	0.005	3.0										6374.531	6939.084	1.898e+004
48	0.009	3.5										2200.404	3597.704	1020.882

#	Anova (p)	Fold	Tags	Notes	pI	MW	Protein Accession	Protein Description	Protein pI	Protein MW	Protein URL	Average Normalised Volumes		
												Control	Ag50	Ag+Amant
12	0.011	3.5										1834.626	4275.045	6343.658
78	0.012	3.0										744.623	520.361	249.267
83	0.014	4.4										3.406e+004	2.336e+004	7714.276
81	0.020	2.4										5.306e+004	3.140e+004	2.186e+004
74	0.025	2.1										3503.955	2617.380	1681.784
44	0.032	1.9										504.825	957.581	506.720

Tags	
	Anova p-value ≤ 0.05
	Max fold change ≥ 1.5

Identifier 80

Position (447, 502)

Notes

Anova p-value ≤ 0.05

Max fold change ≥ 1.5

Control	Ag50	Ag+Amant
20-122015 2DG samples 1 FTSC	20-122015 2DG samples 2 FTSC	20-122015 2DG samples 29 FTSC

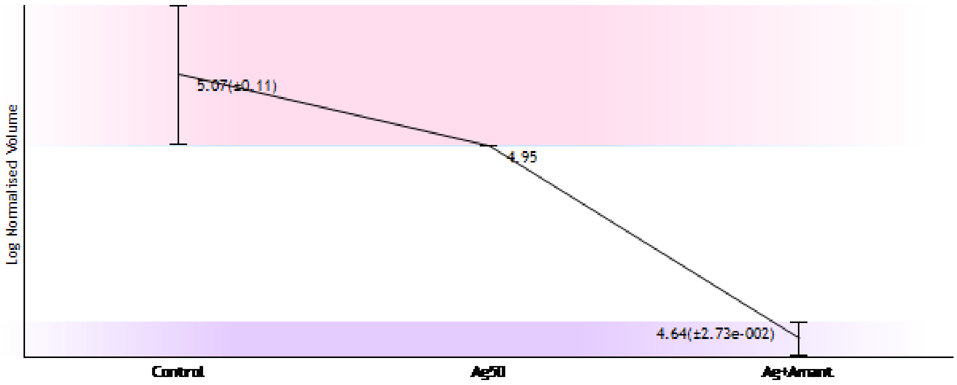

Identifier 11

Position (473, 164)

Notes

Anova p-value ≤ 0.05

Max fold change ≥ 1.5

Control	Ag50	Ag+Amant
20-122015 2DG samples 1 FTSC	20-122015 2DG samples 2 FTSC	20-122015 2DG samples 29 FTSC

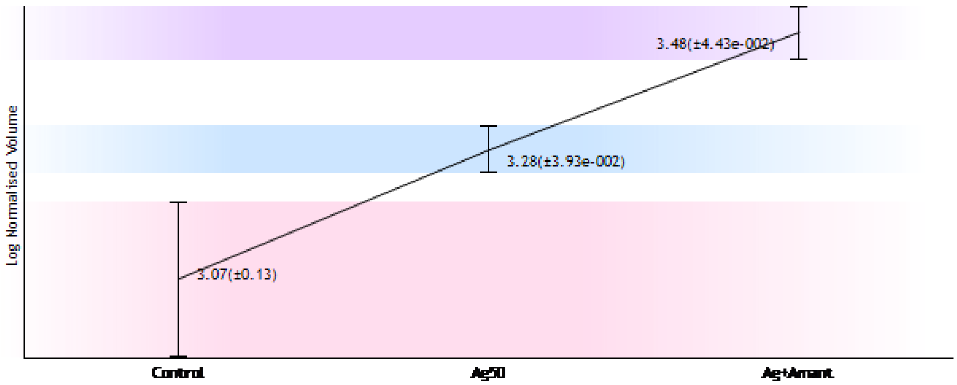

Identifier 7

Position (253, 159)

Notes

- Anova p-value ≤ 0.05
- Max fold change ≥ 1.5

Control	Ag50	Ag+Amant
20-122015 2DG samples 1 FTSC	20-122015 2DG samples 2 FTSC	20-122015 2DG samples 29 FTSC

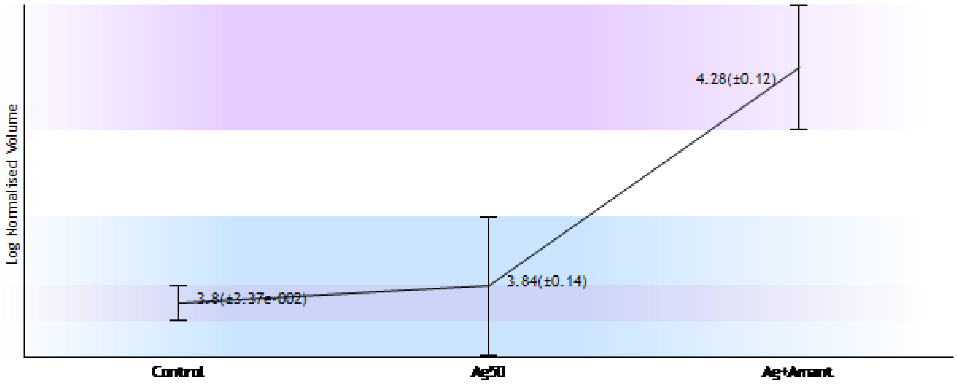

Identifier 48

Position (252, 250)

Notes

- Anova p-value ≤ 0.05
- Max fold change ≥ 1.5

Control	Ag50	Ag+Amant
20-122015 2DG samples 1 FTSC	20-122015 2DG samples 2 FTSC	20-122015 2DG samples 29 FTSC

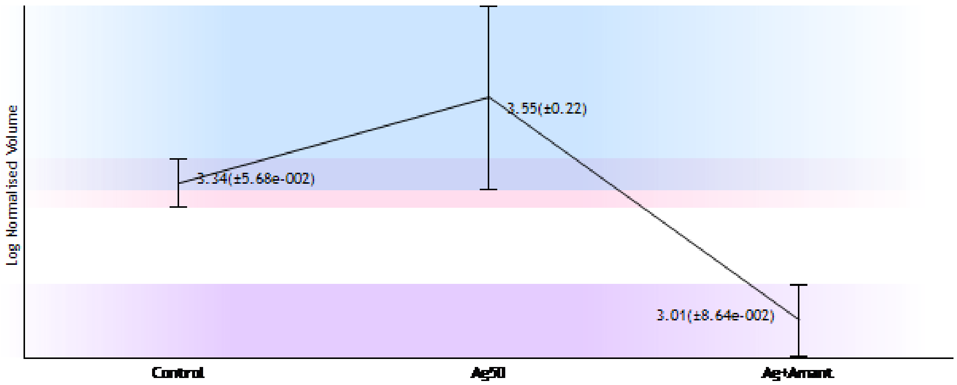

Identifier 12

Position (483, 164)

Notes

- Anova p-value ≤ 0.05
- Max fold change ≥ 1.5

Control	Ag50	Ag+Amant
20-122015 2DG samples 1 FTSC	20-122015 2DG samples 2 FTSC	20-122015 2DG samples 29 FTSC

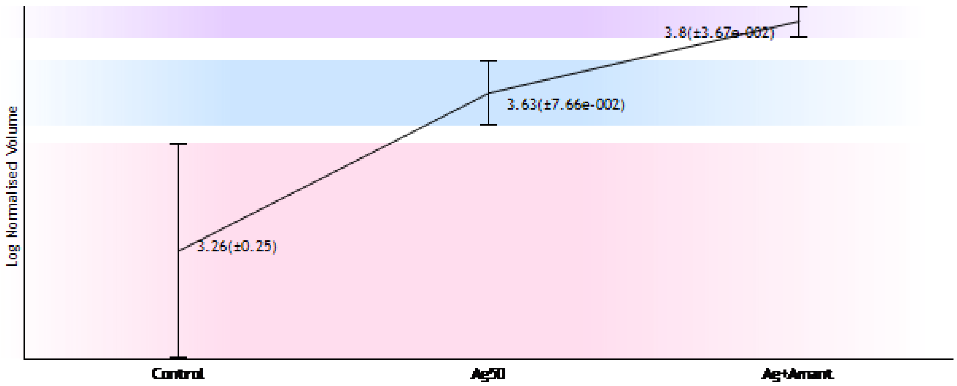

Identifier 78

Position (465, 492)

Notes

- Anova p-value ≤ 0.05
- Max fold change ≥ 1.5

Control	Ag50	Ag+Amant
20-122015 2DG samples 1 FTSC	20-122015 2DG samples 2 FTSC	20-122015 2DG samples 29 FTSC

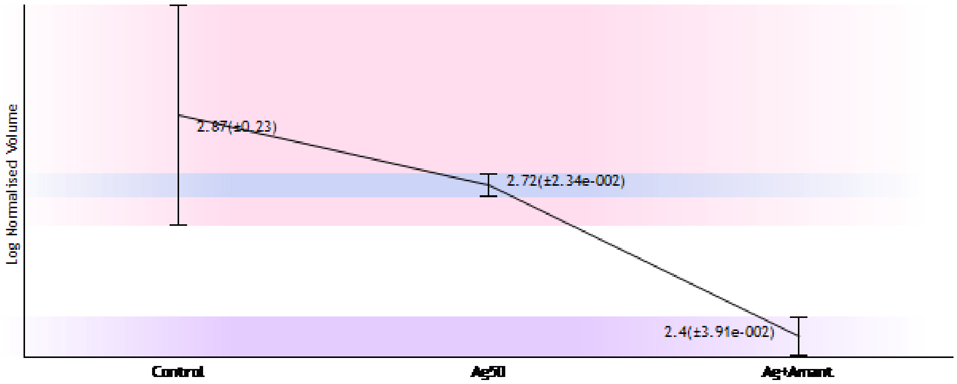

Identifier 83

Position (358, 513)

Notes

- Anova p-value ≤ 0.05
- Max fold change ≥ 1.5

Control	Ag50	Ag+Amant
20-122015 2DG samples 1 FTSC	20-122015 2DG samples 2 FTSC	20-122015 2DG samples 29 FTSC

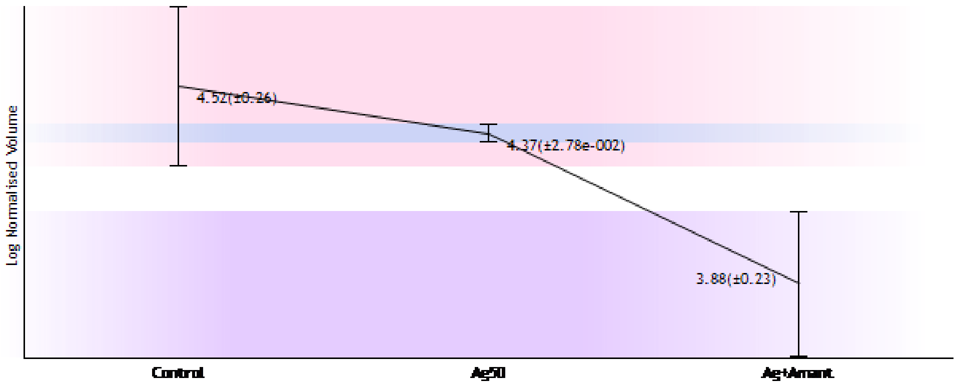

Identifier 81

Position (471, 511)

Notes

- 🟥 Anova p-value ≤ 0.05
- 🟨 Max fold change ≥ 1.5

Control	Ag50	Ag+Amant
20-122015 2DG samples 1 FTSC	20-122015 2DG samples 2 FTSC	20-122015 2DG samples 29 FTSC

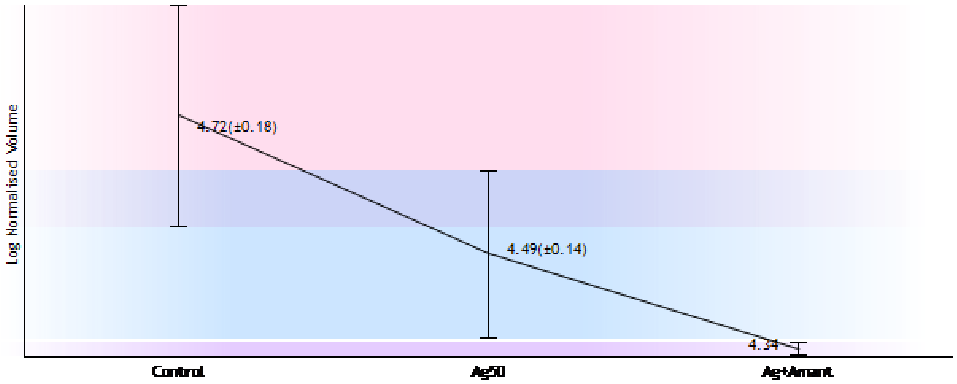

Identifier 74

Position (448, 478)

Notes

- 🟥 Anova p-value ≤ 0.05
- 🟨 Max fold change ≥ 1.5

Control	Ag50	Ag+Amant
20-122015 2DG samples 1 FTSC	20-122015 2DG samples 2 FTSC	20-122015 2DG samples 29 FTSC

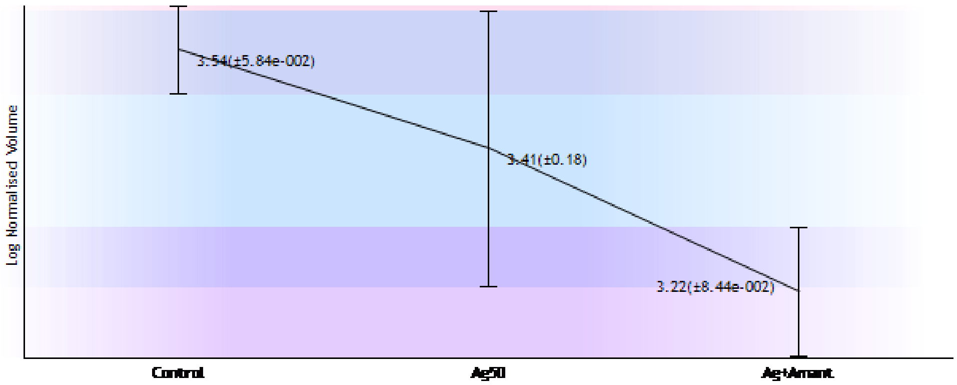

Identifier 44

Position (408, 241)

Notes

- Anova p-value ≤ 0.05
- Max fold change ≥ 1.5

Control	Ag50	Ag+Amant
20-122015 2DG samples 1 FTSC	20-122015 2DG samples 2 FTSC	20-122015 2DG samples 29 FTSC

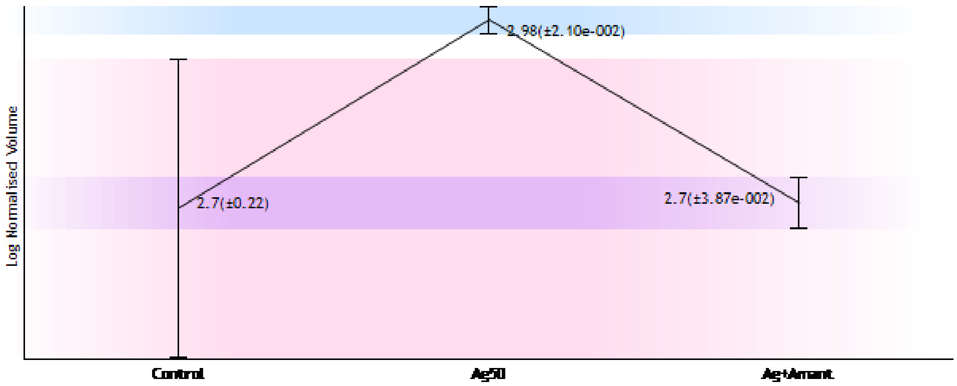

Younes IAF Digestive gland

Experiment: Younes IAF Digestive gland

Report created: 22/12/2015 11:02:18

Reference image

Experiment Design

Condition	Control	Ag50	Ag50+Amant
Replicates	2	2	2

Spots

#	Anova (p)	Fold	Tags	Notes	pI	MW	Protein Accession	Protein Description	Protein pI	Protein MW	Protein URL	Average Normalised Volumes		
												Control	Ag50	Ag50+Amant
211	1.118e-004	5.8										663.436	3852.552	1250.123
36	5.449e-004	1.8										1.021e+004	1.228e+004	1.827e+004
65	6.484e-004	3.2										6.127e+004	1.930e+004	3.642e+004
46	0.001	2.0										9322.541	1.673e+004	1.876e+004
35	0.001	4.2										4432.856	1122.669	1061.093
44	0.002	2.2										7272.546	1.542e+004	1.605e+004
194	0.002	1.6										1.171e+004	1.896e+004	1.600e+004
29	0.005	1.9										2027.039	3907.054	2356.340
58	0.007	1.9										8315.909	1.602e+004	1.349e+004
113	0.011	1.6										6658.466	4275.475	6521.993
90	0.013	1.7										9.880e+004	7.466e+004	5.805e+004
48	0.013	2.7										3839.436	1.018e+004	8819.291
57	0.013	2.0										4091.955	8157.607	8065.854
99	0.013	2.5										2.127e+004	1.178e+004	8358.439
47	0.014	1.8										3201.252	5160.215	5878.229
171	0.017	1.5										3.857e+004	2.865e+004	2.505e+004
96	0.018	1.9										1035.328	1955.185	1222.046
101	0.019	1.7										9136.595	7404.308	1.264e+004
18	0.021	3.1										1891.597	795.688	605.972
223	0.024	2.3										239.893	168.825	379.876
51	0.024	2.7										5003.319	1.339e+004	7728.837
97	0.027	2.7										1406.071	3348.638	3792.241
86	0.030	2.2										3608.726	7780.496	6373.143
177	0.030	2.5										625.558	573.773	251.776
185	0.030	1.7										3.060e+004	2.350e+004	1.767e+004
217	0.031	2.3										1.095e+004	5436.616	4684.273
56	0.033	2.1										1.579e+004	9324.592	7633.617
60	0.033	2.7										2.506e+004	1.944e+004	9236.557
72	0.034	2.4										4687.670	9106.499	1.137e+004
120	0.035	2.5										3.286e+004	5.635e+004	8.098e+004
80	0.036	1.6										3050.888	3472.243	5015.829
213	0.037	1.6										754.113	863.334	531.155
27	0.040	12.0										486.673	1471.118	5829.466
102	0.041	1.7										6036.351	1.005e+004	9585.897

Tags	
🟥	Anova p-value ≤ 0.05
🟨	Max fold change ≥ 1.5

Identifier 211

Position (467, 437)

Notes

- 🟥 Anova p-value ≤ 0.05
- 🟨 Max fold change ≥ 1.5

Control	Ag50	Ag50+Amant
19-12-2015 2DG samples 37 IAF	19-12-2015 2DG samples 38 IAF	19-12-2015 2DG samples 65 IAF

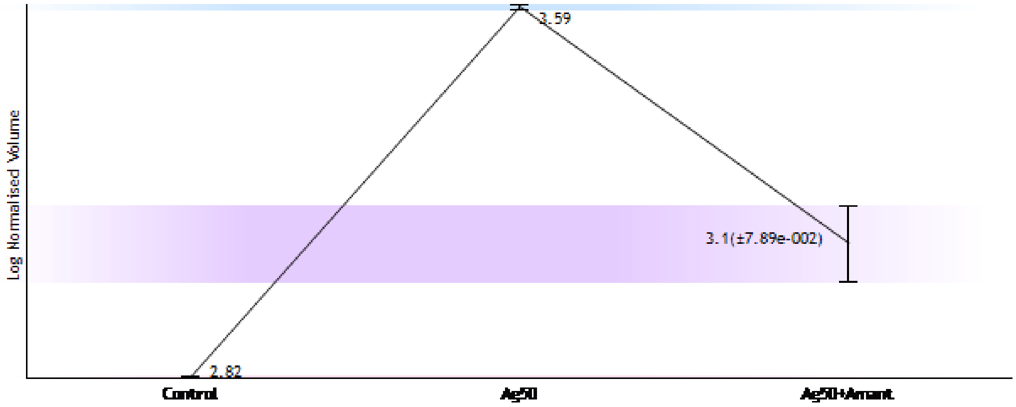

Identifier 36

Position (316, 151)

Notes

- 🟥 Anova p-value ≤ 0.05
- 🟨 Max fold change ≥ 1.5

Control	Ag50	Ag50+Amant
19-12-2015 2DG samples 37 IAF	19-12-2015 2DG samples 38 IAF	19-12-2015 2DG samples 65 IAF

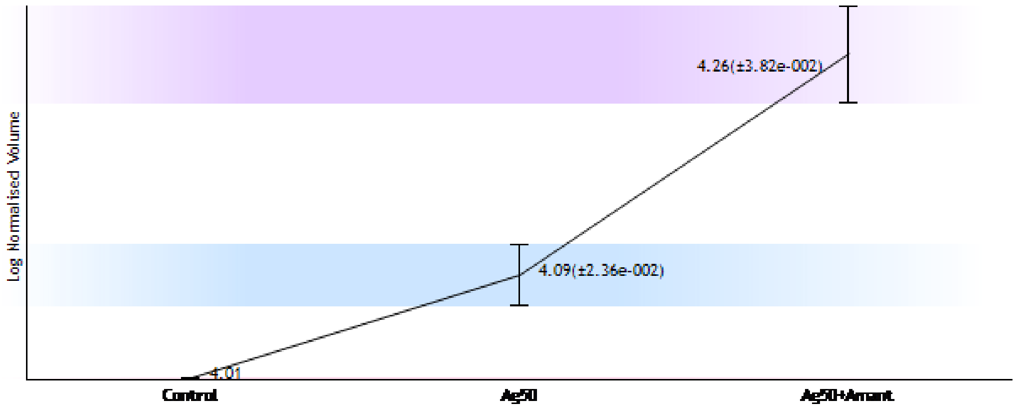

Identifier 65

Position (688, 166)

Notes

Anova p-value ≤ 0.05

Max fold change ≥ 1.5

Control	Ag50	Ag50+Amant
19-12-2015 2DG samples 37 IAF	19-12-2015 2DG samples 38 IAF	19-12-2015 2DG samples 65 IAF

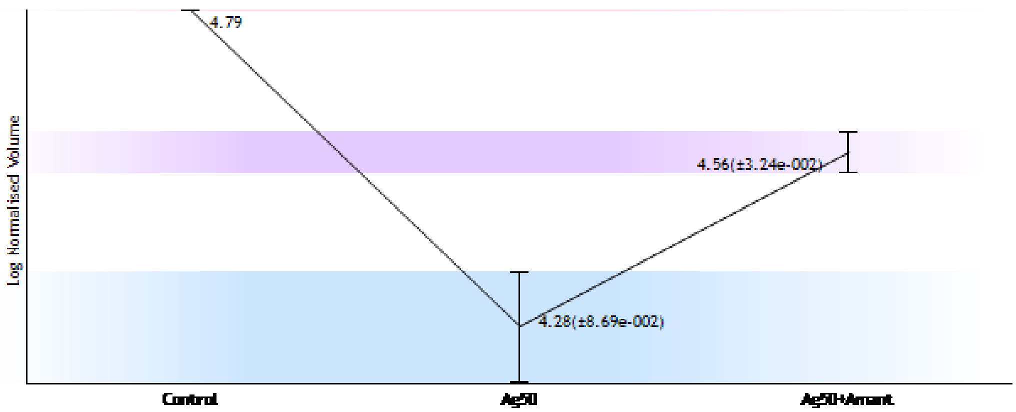

Identifier 46

Position (331, 161)

Notes

Anova p-value ≤ 0.05

Max fold change ≥ 1.5

Control	Ag50	Ag50+Amant
19-12-2015 2DG samples 37 IAF	19-12-2015 2DG samples 38 IAF	19-12-2015 2DG samples 65 IAF

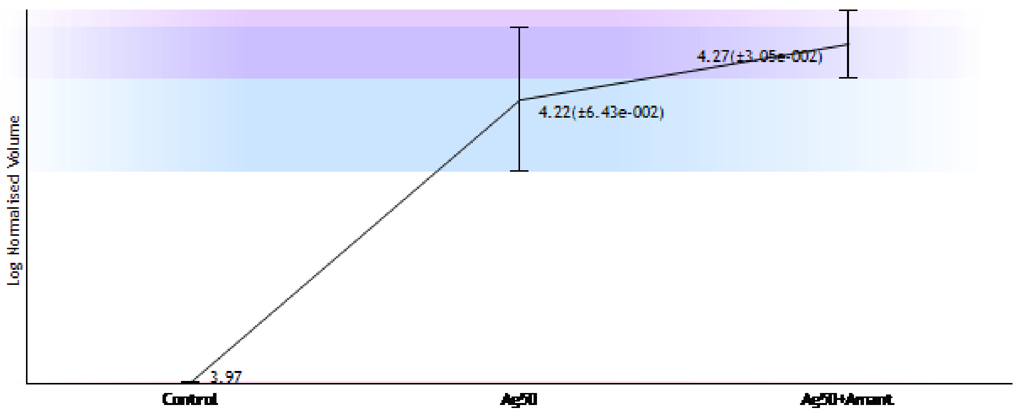

Identifier 35

Position (239, 149)

Notes

Anova p-value ≤ 0.05

Max fold change ≥ 1.5

Control	Ag50	Ag50+Amant
19-12-2015 2DG samples 37 IAF	19-12-2015 2DG samples 38 IAF	19-12-2015 2DG samples 65 IAF

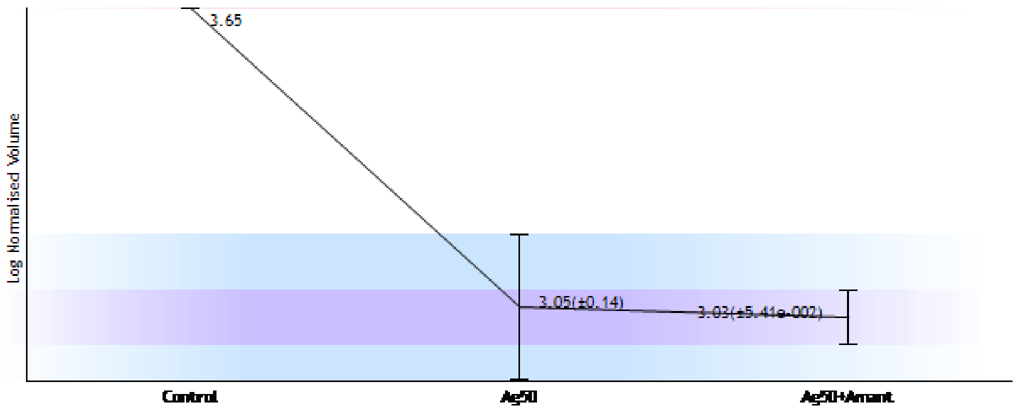

Identifier 44

Position (287, 159)

Notes

- Anova p-value ≤ 0.05
- Max fold change ≥ 1.5

Control	Ag50	Ag50+Amant
19-12-2015 2DG samples 37 IAF	19-12-2015 2DG samples 38 IAF	19-12-2015 2DG samples 65 IAF

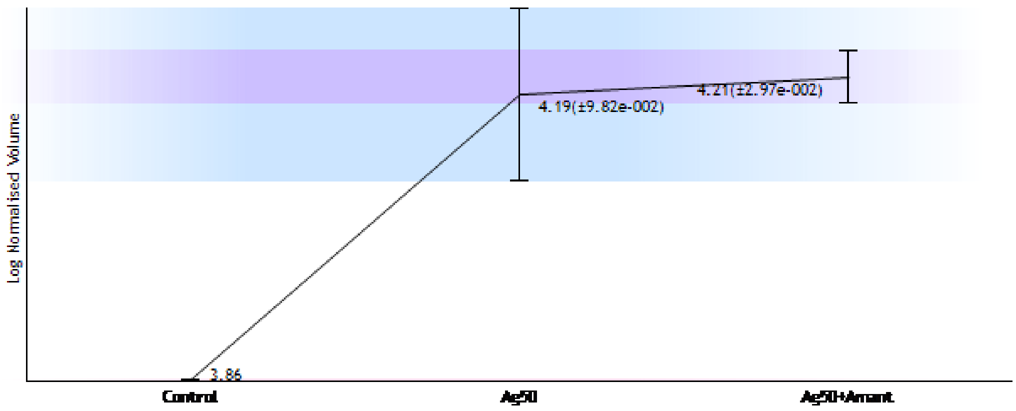

Identifier 194

Position (386, 393)

Notes

- Anova p-value ≤ 0.05
- Max fold change ≥ 1.5

Control	Ag50	Ag50+Amant
19-12-2015 2DG samples 37 IAF	19-12-2015 2DG samples 38 IAF	19-12-2015 2DG samples 65 IAF

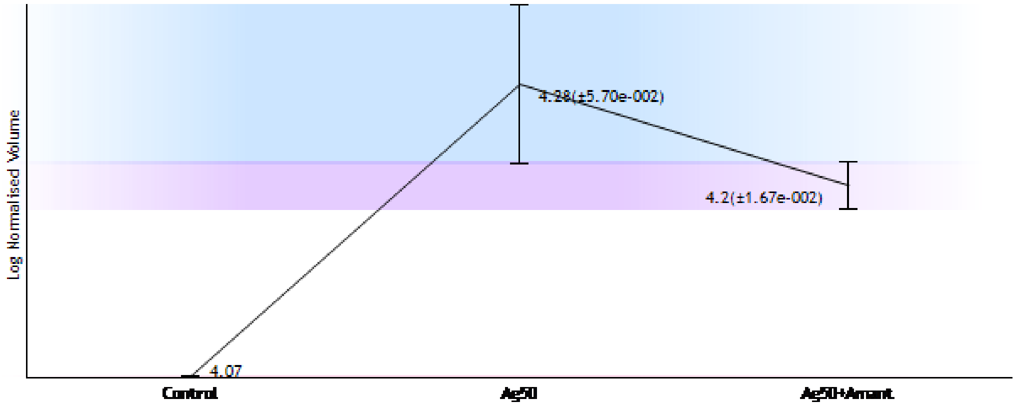

Identifier 29

Position (269, 137)

Notes

- Anova p-value ≤ 0.05
- Max fold change ≥ 1.5

Control	Ag50	Ag50+Amant
19-12-2015 2DG samples 37 IAF	19-12-2015 2DG samples 38 IAF	19-12-2015 2DG samples 65 IAF

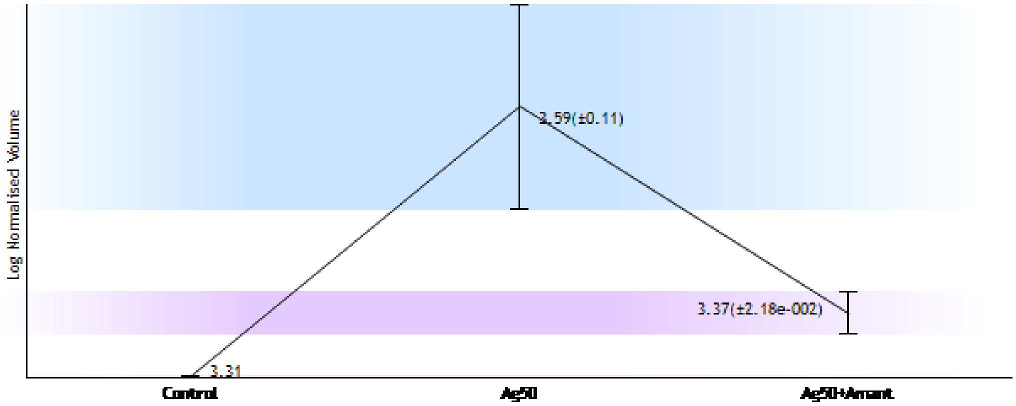

Identifier 58

Position (409, 163)

Notes

- Anova p-value ≤ 0.05
- Max fold change ≥ 1.5

Control	Ag50	Ag50+Amant
19-12-2015 2DG samples 37 IAF	19-12-2015 2DG samples 38 IAF	19-12-2015 2DG samples 65 IAF

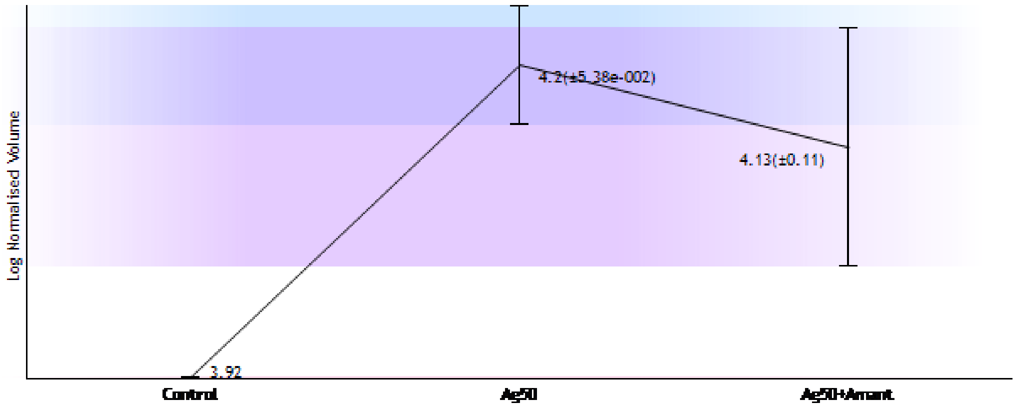

Identifier 113

Position (544, 237)

Notes

- Anova p-value ≤ 0.05
- Max fold change ≥ 1.5

Control	Ag50	Ag50+Amant
19-12-2015 2DG samples 37 IAF	19-12-2015 2DG samples 38 IAF	19-12-2015 2DG samples 65 IAF

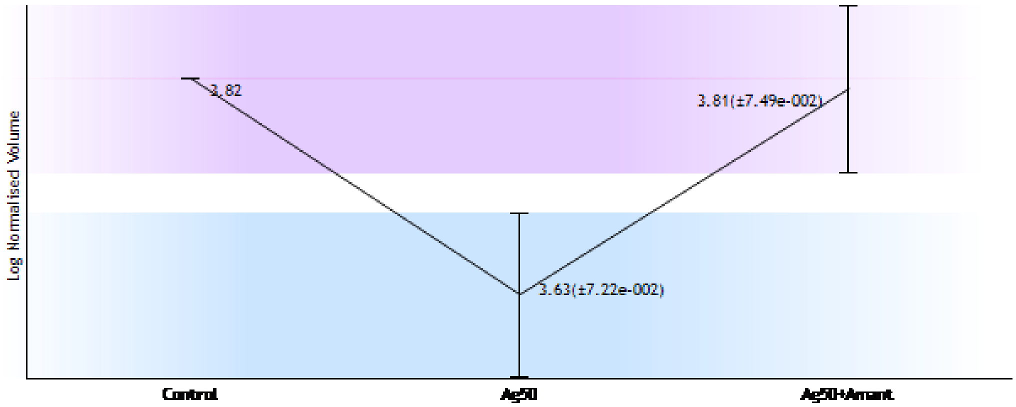

Identifier 90

Position (240, 214)

Notes

- Anova p-value ≤ 0.05
- Max fold change ≥ 1.5

Control	Ag50	Ag50+Amant
19-12-2015 2DG samples 37 IAF	19-12-2015 2DG samples 38 IAF	19-12-2015 2DG samples 65 IAF

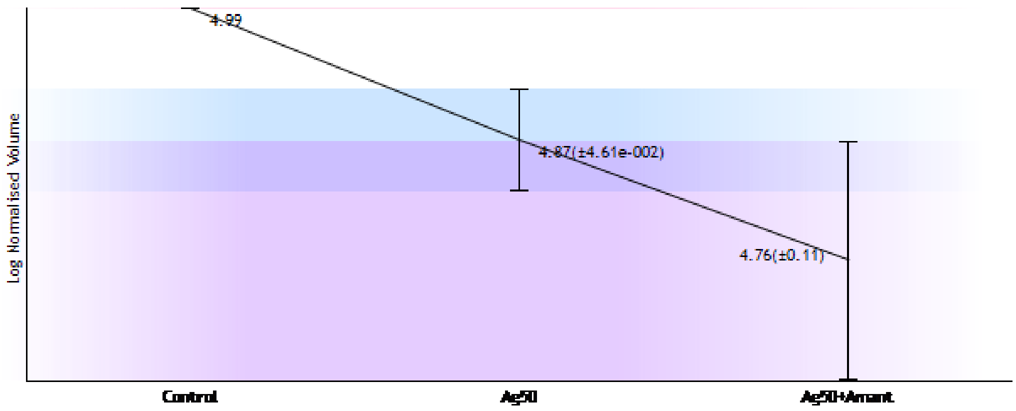

Identifier 48

Position (381, 161)

Notes

- Anova p-value ≤ 0.05
- Max fold change ≥ 1.5

Control	Ag50	Ag50+Amant
19-12-2015 2DG samples 37 IAF	19-12-2015 2DG samples 38 IAF	19-12-2015 2DG samples 65 IAF

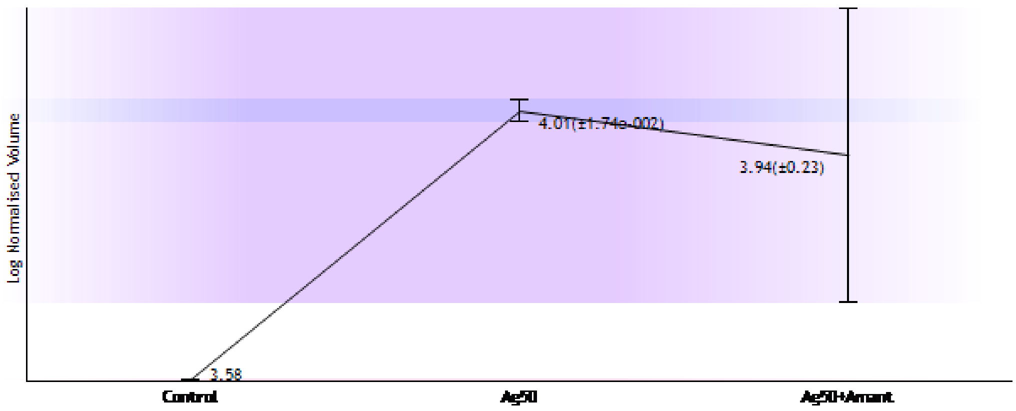

Identifier 57

Position (348, 163)

Notes

- Anova p-value ≤ 0.05
- Max fold change ≥ 1.5

Control	Ag50	Ag50+Amant
19-12-2015 2DG samples 37 IAF	19-12-2015 2DG samples 38 IAF	19-12-2015 2DG samples 65 IAF

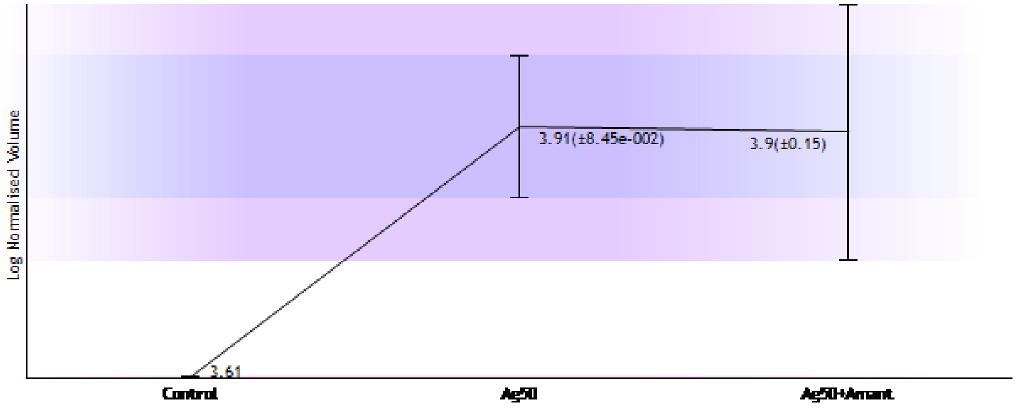

Identifier 99

Position (600, 224)

Notes

- Anova p-value ≤ 0.05
- Max fold change ≥ 1.5

Control	Ag50	Ag50+Amant
19-12-2015 2DG samples 37 IAF	19-12-2015 2DG samples 38 IAF	19-12-2015 2DG samples 65 IAF

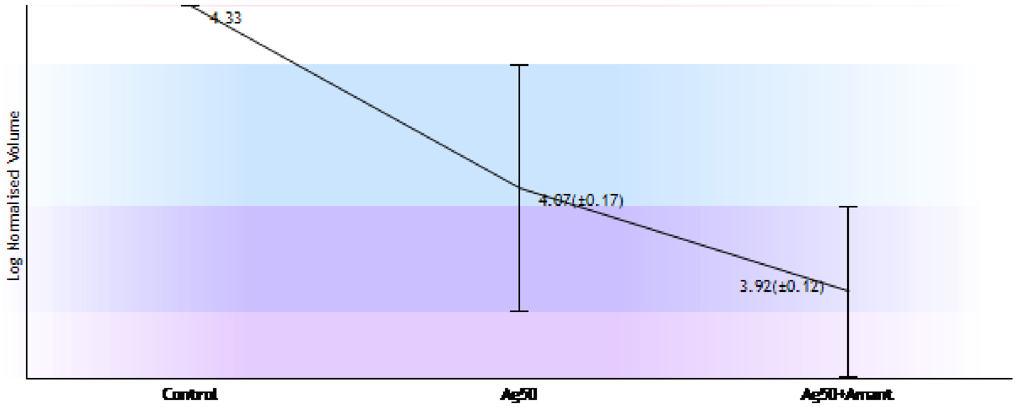

Identifier 47

Position (366, 161)

Notes

- Anova p-value ≤ 0.05
- Max fold change ≥ 1.5

Control	Ag50	Ag50+Amant
19-12-2015 2DG samples 37 IAF	19-12-2015 2DG samples 38 IAF	19-12-2015 2DG samples 65 IAF

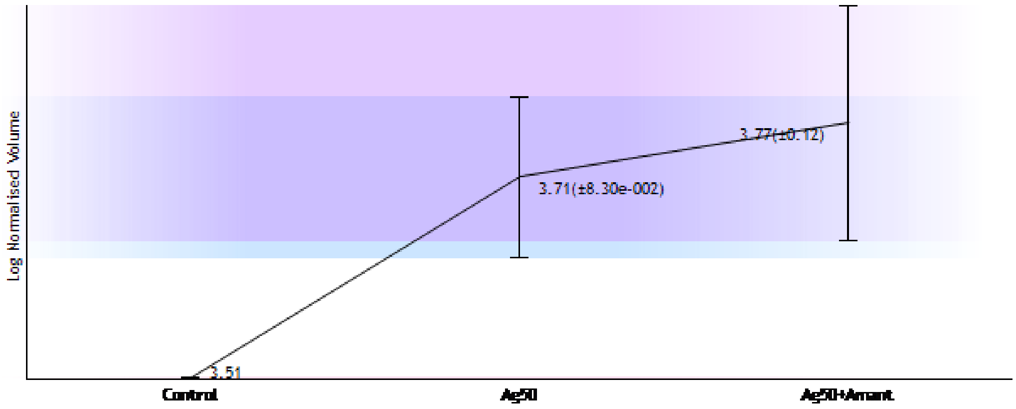

Identifier 171

Position (596, 356)

Notes

- Anova p-value ≤ 0.05
- Max fold change ≥ 1.5

Control	Ag50	Ag50+Amant
19-12-2015 2DG samples 37 IAF	19-12-2015 2DG samples 38 IAF	19-12-2015 2DG samples 65 IAF

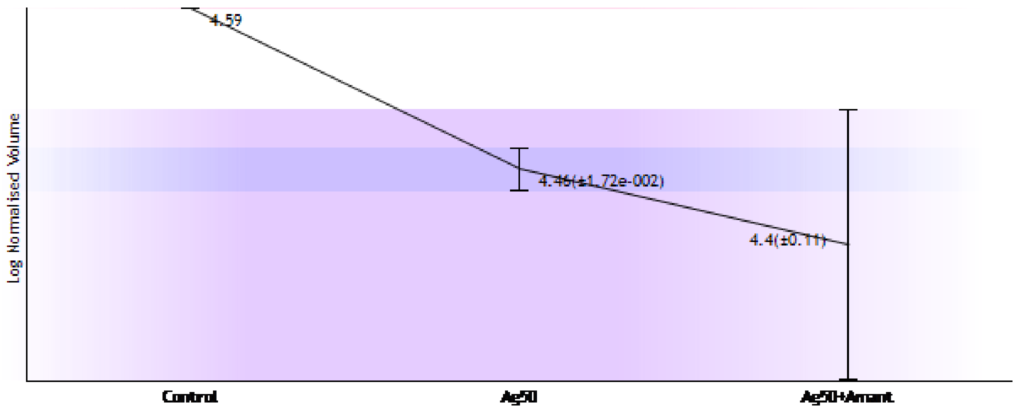

Identifier 96

Position (344, 222)

Notes

- Anova p-value ≤ 0.05
- Max fold change ≥ 1.5

Control	Ag50	Ag50+Amant
19-12-2015 2DG samples 37 IAF	19-12-2015 2DG samples 38 IAF	19-12-2015 2DG samples 65 IAF

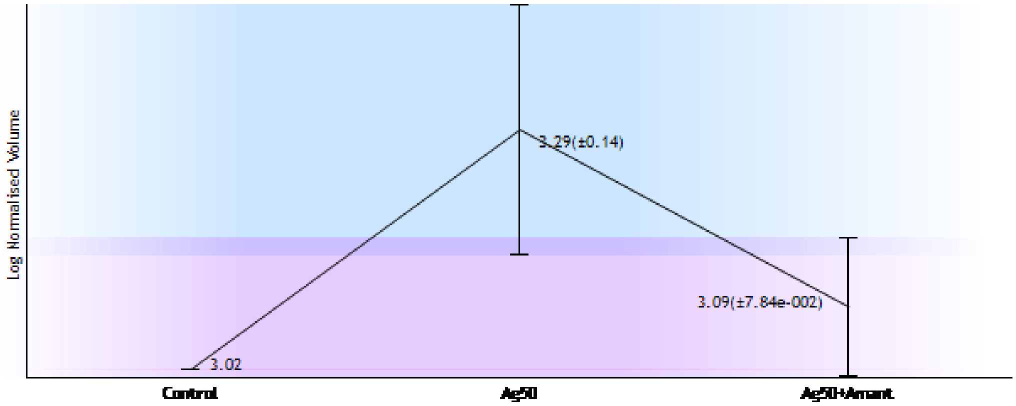

Identifier 101

Position (568, 225)

Notes

- Anova p-value ≤ 0.05
- Max fold change ≥ 1.5

Control	Ag50	Ag50+Amant
19-12-2015 2DG samples 37 IAF	19-12-2015 2DG samples 38 IAF	19-12-2015 2DG samples 65 IAF

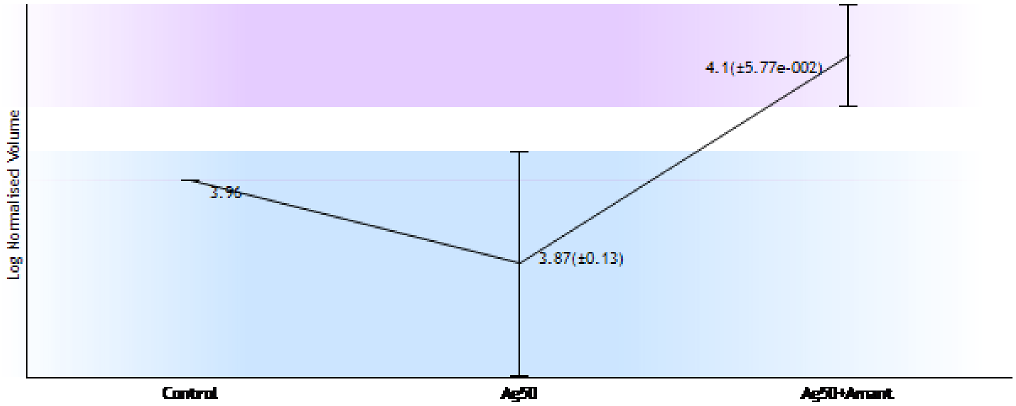

Identifier 18

Position (628, 115)

Notes

- Anova p-value ≤ 0.05
- Max fold change ≥ 1.5

Control	Ag50	Ag50+Amant
19-12-2015 2DG samples 37 IAF	19-12-2015 2DG samples 38 IAF	19-12-2015 2DG samples 65 IAF

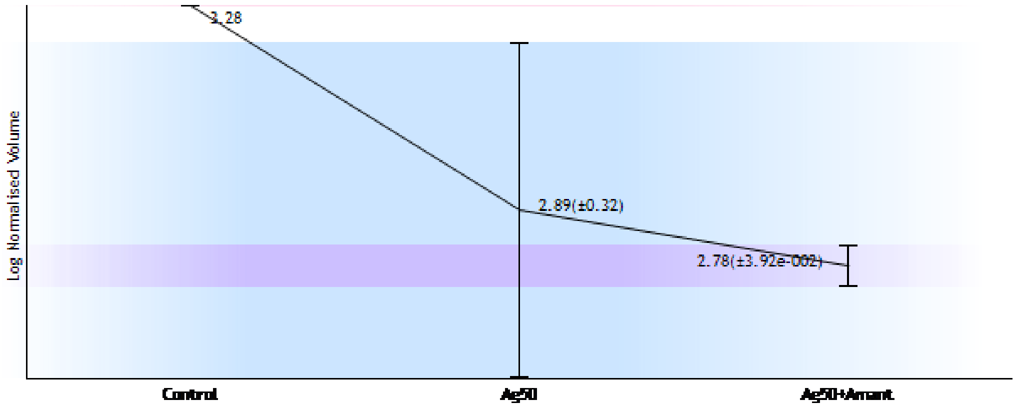

Identifier 223

Position (603, 525)

Notes

- Anova p-value ≤ 0.05
- Max fold change ≥ 1.5

Control	Ag50	Ag50+Amant
19-12-2015 2DG samples 37 IAF	19-12-2015 2DG samples 38 IAF	19-12-2015 2DG samples 65 IAF

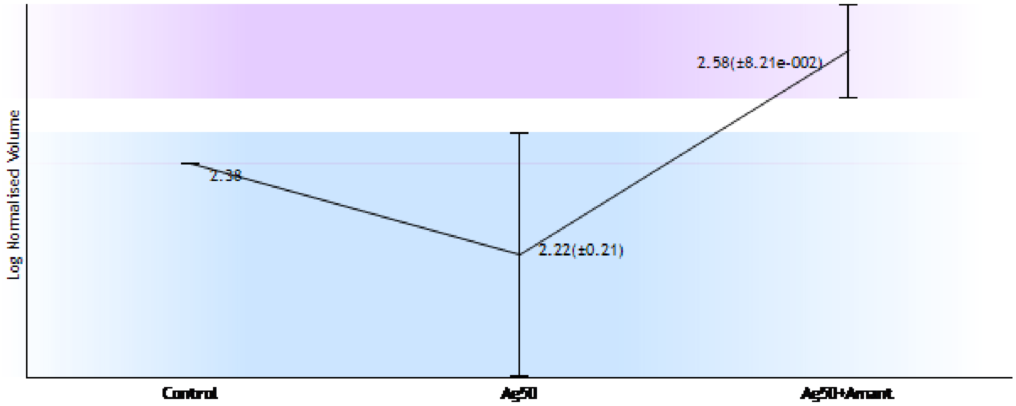

Identifier 51

Position (394, 162)

Notes

- Anova p-value ≤ 0.05
- Max fold change ≥ 1.5

Control	Ag50	Ag50+Amant
19-12-2015 2DG samples 37 IAF	19-12-2015 2DG samples 38 IAF	19-12-2015 2DG samples 65 IAF

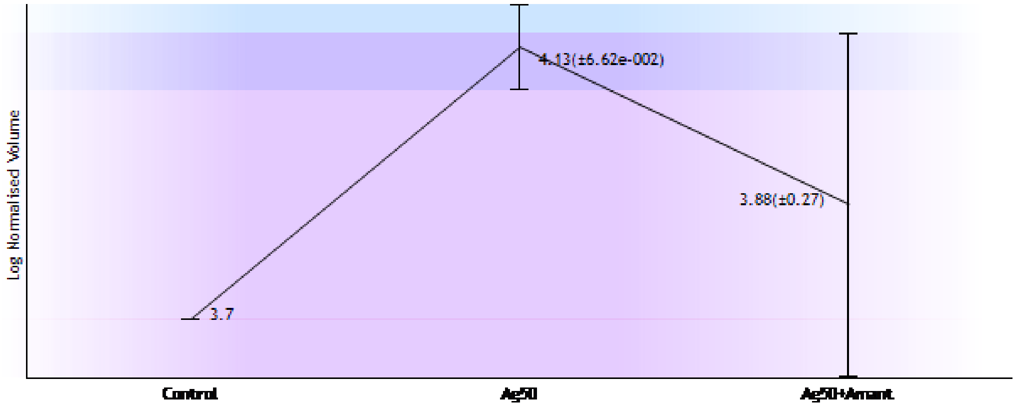

Identifier 97

Position (355, 224)

Notes

- Anova p-value ≤ 0.05
- Max fold change ≥ 1.5

Control	Ag50	Ag50+Amant
19-12-2015 2DG samples 37 IAF	19-12-2015 2DG samples 38 IAF	19-12-2015 2DG samples 65 IAF

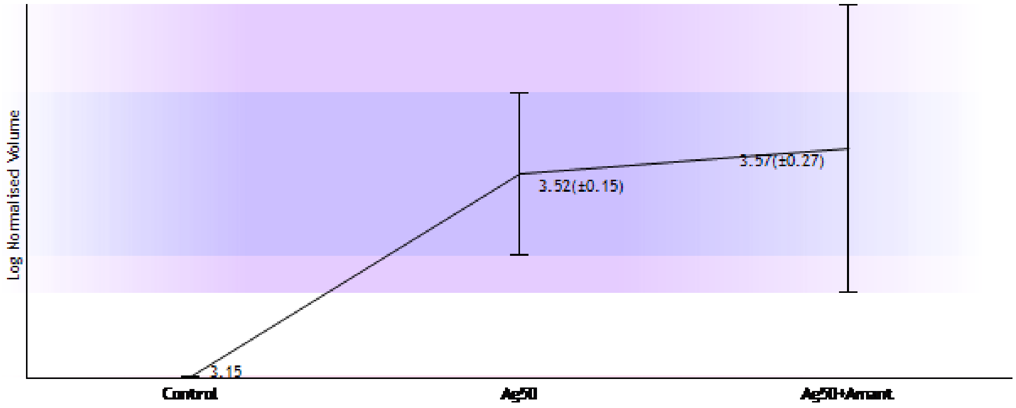

Identifier 86

Position (402, 207)

Notes

- Anova p-value ≤ 0.05
- Max fold change ≥ 1.5

Control	Ag50	Ag50+Amant
19-12-2015 2DG samples 37 IAF	19-12-2015 2DG samples 38 IAF	19-12-2015 2DG samples 65 IAF

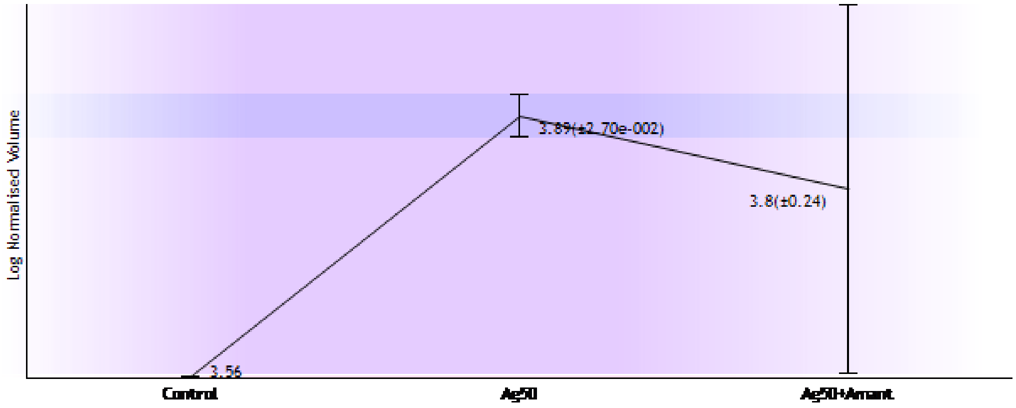

Identifier 177

Position (328, 368)

Notes

- Anova p-value ≤ 0.05
- Max fold change ≥ 1.5

Control	Ag50	Ag50+Amant
19-12-2015 2DG samples 37 IAF	19-12-2015 2DG samples 38 IAF	19-12-2015 2DG samples 65 IAF

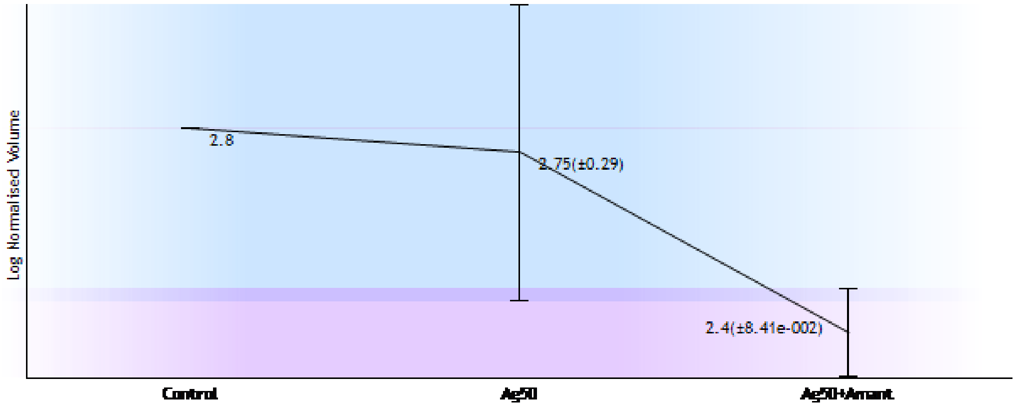

Identifier 185

Position (595, 379)

Notes

- Anova p-value ≤ 0.05
- Max fold change ≥ 1.5

Control	Ag50	Ag50+Amant
19-12-2015 2DG samples 37 IAF	19-12-2015 2DG samples 38 IAF	19-12-2015 2DG samples 65 IAF

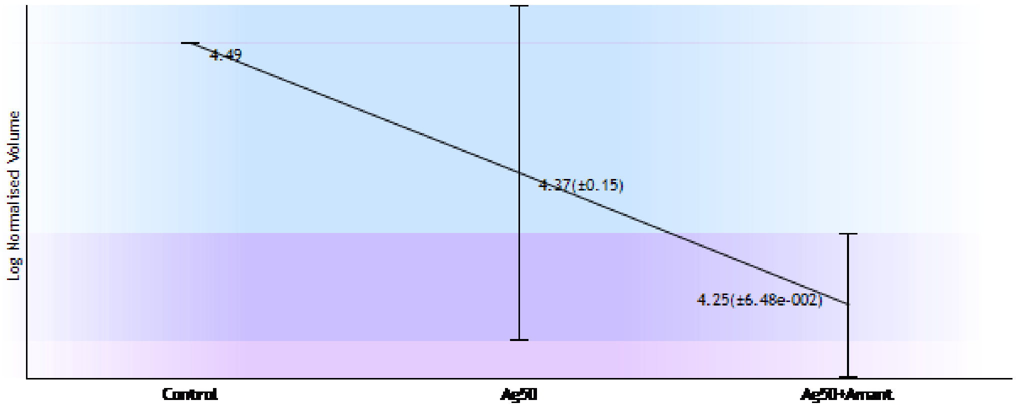

Identifier 217

Position (303, 472)

Notes

- Anova p-value ≤ 0.05
- Max fold change ≥ 1.5

Control	Ag50	Ag50+Amant
19-12-2015 2DG samples 37 IAF	19-12-2015 2DG samples 38 IAF	19-12-2015 2DG samples 65 IAF

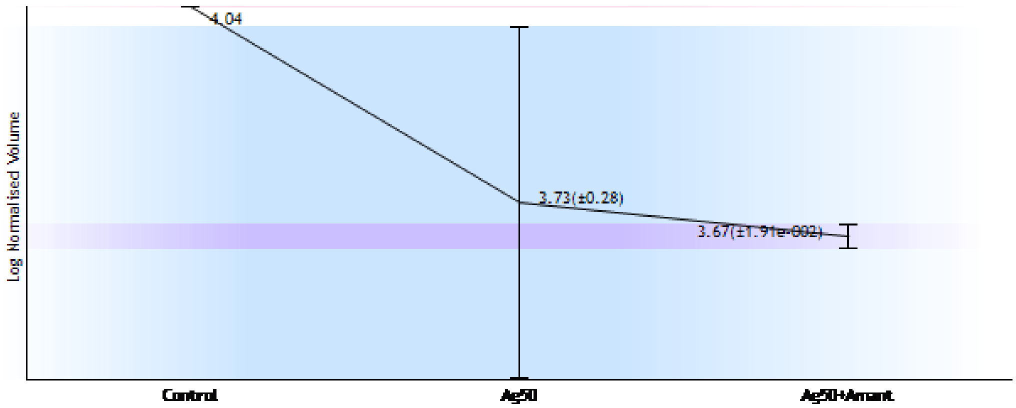

Identifier 56

Position (657, 162)

Notes

- Anova p-value ≤ 0.05
- Max fold change ≥ 1.5

Control	Ag50	Ag50+Amant
19-12-2015 2DG samples 37 IAF	19-12-2015 2DG samples 38 IAF	19-12-2015 2DG samples 65 IAF

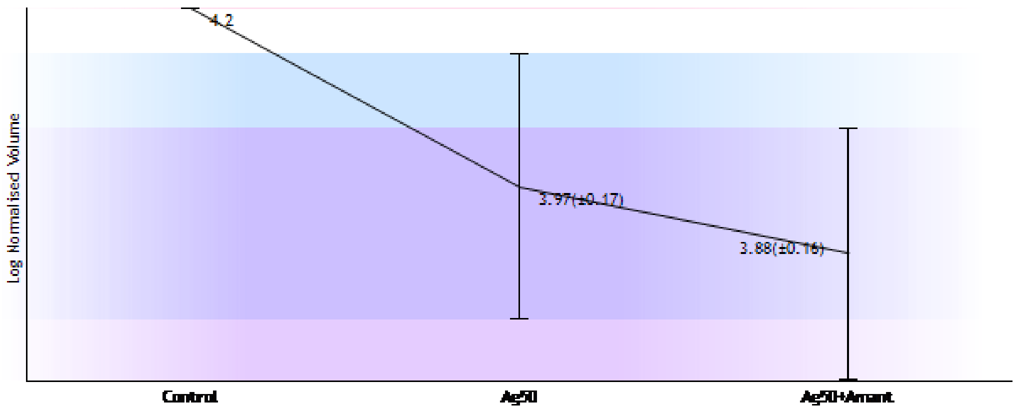

Identifier 60

Position (602, 163)

Notes

- Anova p-value ≤ 0.05
- Max fold change ≥ 1.5

Control	Ag50	Ag50+Amant
19-12-2015 2DG samples 37 IAF	19-12-2015 2DG samples 38 IAF	19-12-2015 2DG samples 65 IAF

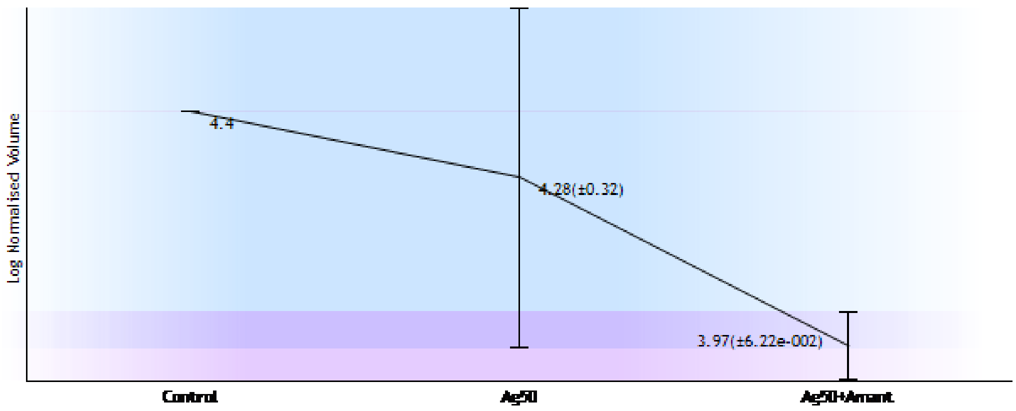

Identifier 72

Position (287, 181)

Notes

- Anova p-value ≤ 0.05
- Max fold change ≥ 1.5

Control	Ag50	Ag50+Amant
19-12-2015 2DG samples 37 IAF	19-12-2015 2DG samples 38 IAF	19-12-2015 2DG samples 65 IAF

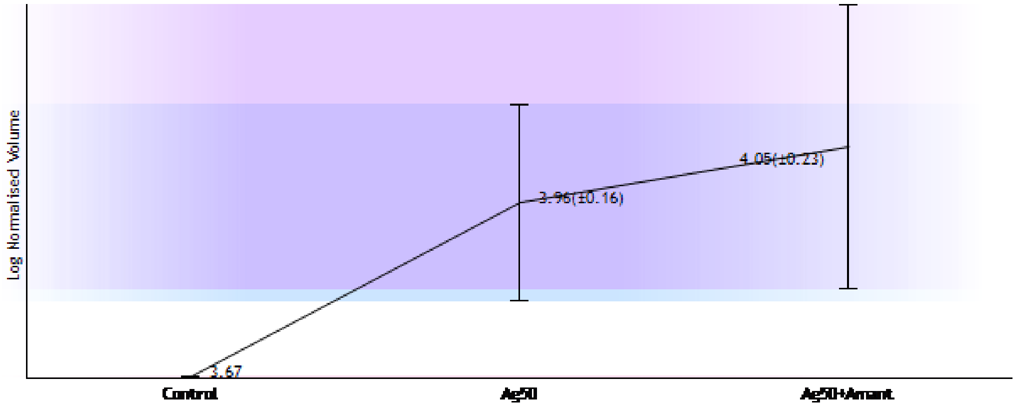

Identifier 120

Position (159, 247)

Notes

- Anova p-value ≤ 0.05
- Max fold change ≥ 1.5

Control	Ag50	Ag50+Amant
19-12-2015 2DG samples 37 IAF	19-12-2015 2DG samples 38 IAF	19-12-2015 2DG samples 65 IAF

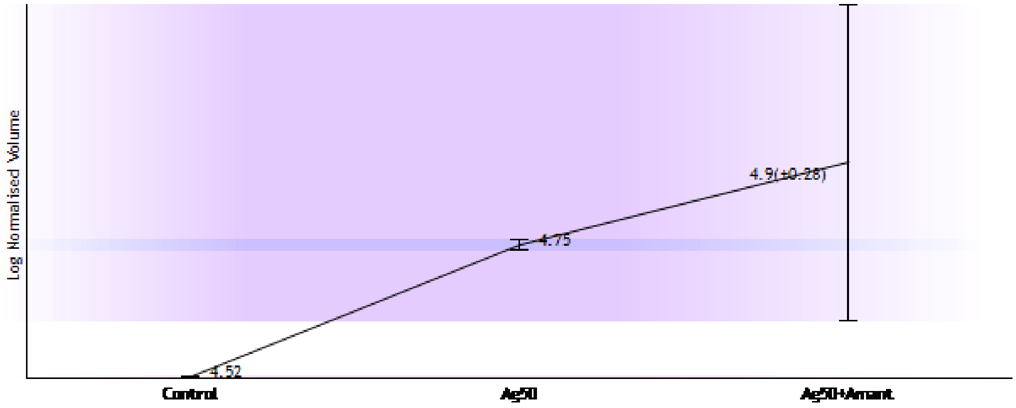

Identifier 80

Position (569, 197)

Notes

- Anova p-value ≤ 0.05
- Max fold change ≥ 1.5

Control	Ag50	Ag50+Amant
19-12-2015 2DG samples 37 IAF	19-12-2015 2DG samples 38 IAF	19-12-2015 2DG samples 65 IAF

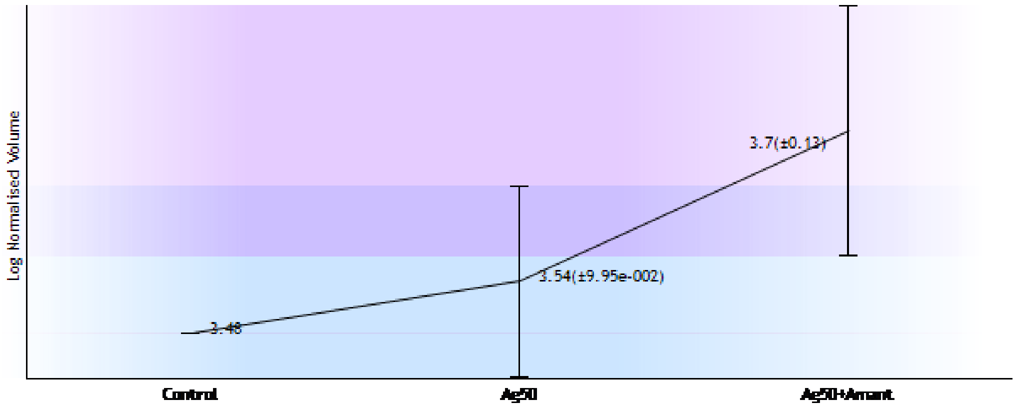

Identifier 213

Position (717, 449)

Notes

- Anova p-value ≤ 0.05
- Max fold change ≥ 1.5

Control	Ag50	Ag50+Amant
19-12-2015 2DG samples 37 IAF	19-12-2015 2DG samples 38 IAF	19-12-2015 2DG samples 65 IAF

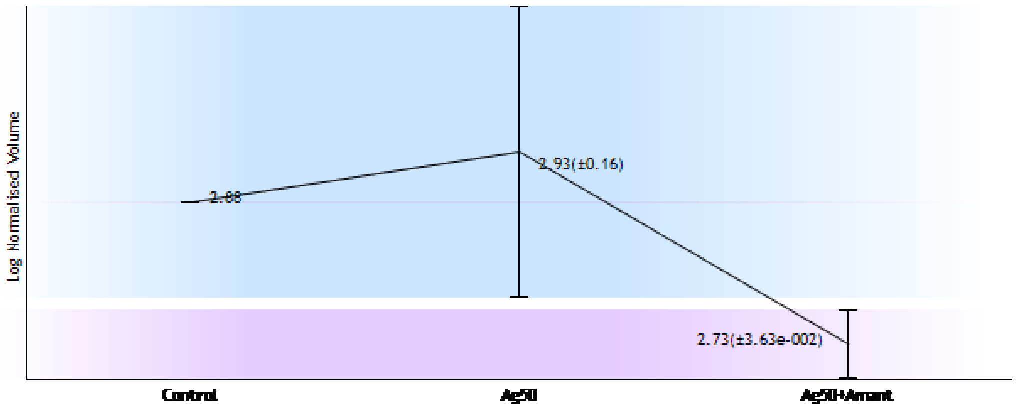

Identifier 27

Position (347, 130)

Notes

- Anova p-value ≤ 0.05
- Max fold change ≥ 1.5

Control	Ag50	Ag50+Amant
19-12-2015 2DG samples 37 IAF	19-12-2015 2DG samples 38 IAF	19-12-2015 2DG samples 65 IAF

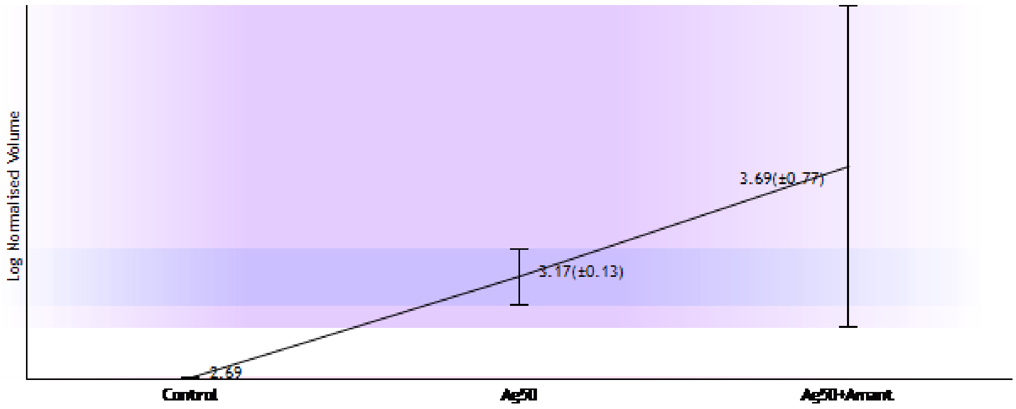

Identifier 102

Position (374, 226)

Notes

- Anova p-value ≤ 0.05
- Max fold change ≥ 1.5

Control	Ag50	Ag50+Amant
19-12-2015 2DG samples 37 IAF	19-12-2015 2DG samples 38 IAF	19-12-2015 2DG samples 65 IAF

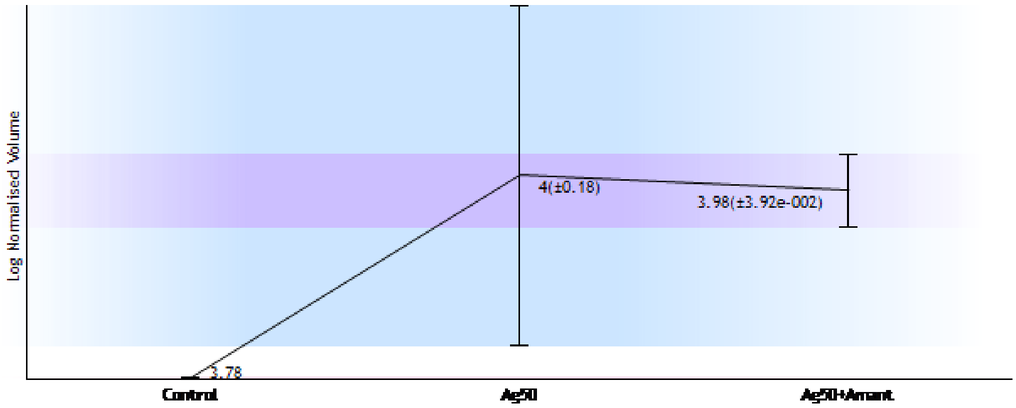

Younes IAF Gills

Experiment: Younes IAF Gills

Report created: 22/12/2015 11:27:09

Reference image

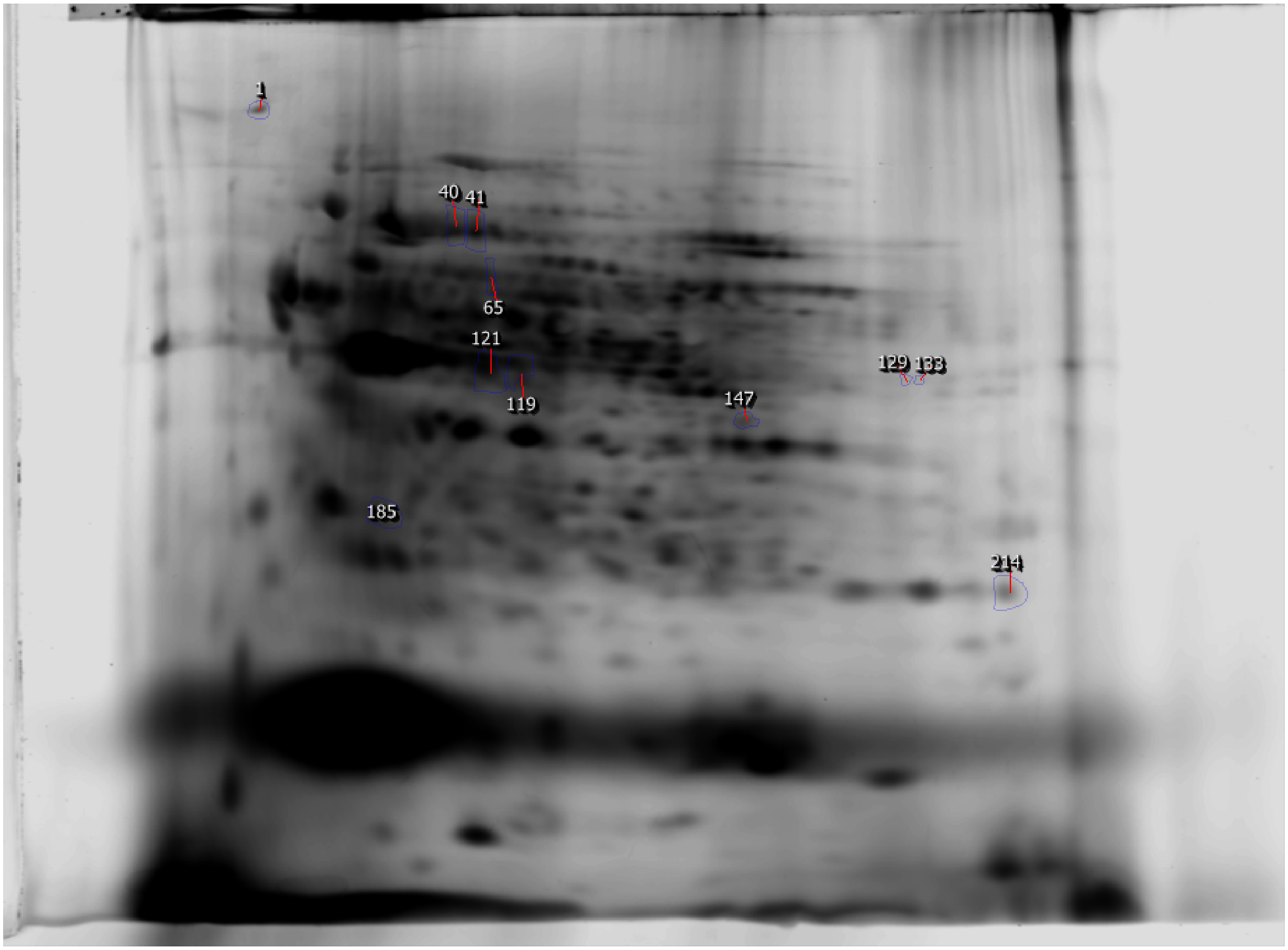

Experiment Design

Condition	Control	Ag50	Ag50+Amant
Replicates	2	2	2

Spots

#	Anova (p)	Fold	Tags	Notes	pI	MW	Protein Accession	Protein Description	Protein pI	Protein MW	Protein URL	Average Normalised Volumes		
												Control	Ag50	Ag50+Amant
119	0.003	1.9										7630.601	1.276e+004	6718.368
41	0.003	1.5										7842.361	6956.848	5147.865
1	0.004	6.2										1941.800	2607.275	420.348
129	0.005	5.3										146.132	78.960	415.796
147	0.018	2.1										1865.296	3094.422	1443.348

#	Anova (p)	Fold	Tags	Notes	pI	MW	Protein Accession	Protein Description	Protein pI	Protein MW	Protein URL	Average Normalised Volumes		
												Control	Ag50	Ag50+Amant
133	0.026	2.6										120.473	99.564	260.808
121	0.035	1.5										1.672e+004	2.187e+004	1.430e+004
185	0.043	2.0										4831.508	6378.751	3241.754
214	0.047	1.9										4731.172	9152.301	5744.178
65	0.048	1.6										3121.582	4994.341	3163.863
40	0.049	1.5										8183.723	5850.514	5415.280

Tags	
	Anova p-value ≤ 0.05
	Max fold change ≥ 1.5

Identifier 119

Position (351, 245)

Notes

- Anova p-value ≤ 0.05
- Max fold change ≥ 1.5

Control	Ag50	Ag50+Amant
20-12-2015 2DG samples 1 IAF	20-12-2015 2DG samples 2 IAF	20-12-2015 2DG samples 29 IAF

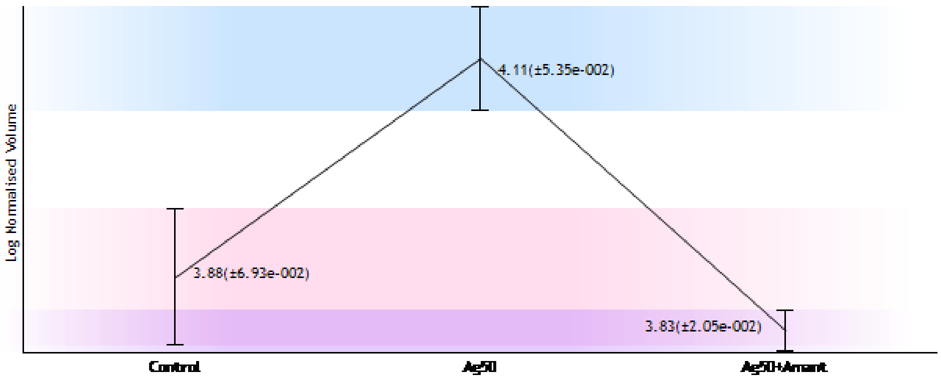

Identifier 41

Position (325, 156)

Notes

- Anova p-value ≤ 0.05
- Max fold change ≥ 1.5

Control	Ag50	Ag50+Amant
20-12-2015 2DG samples 1 IAF	20-12-2015 2DG samples 2 IAF	20-12-2015 2DG samples 29 IAF

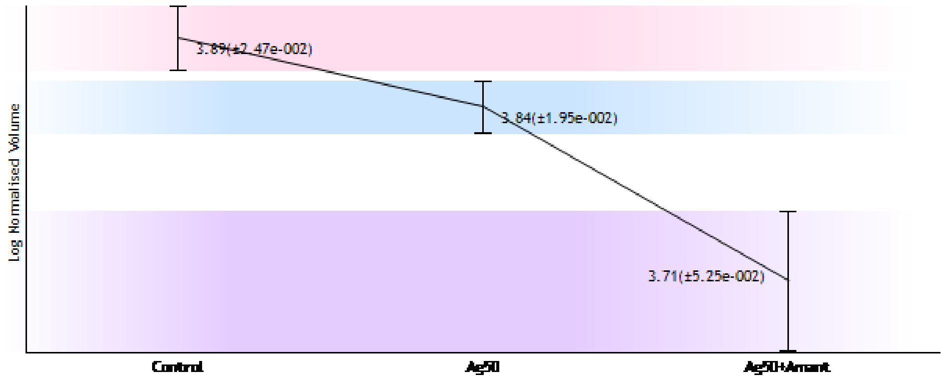

Identifier 1

Position (176, 72)

Notes

- Anova p-value ≤ 0.05
- Max fold change ≥ 1.5

Control	Ag50	Ag50+Amant
20-12-2015 2DG samples 1 IAF	20-12-2015 2DG samples 2 IAF	20-12-2015 2DG samples 29 IAF

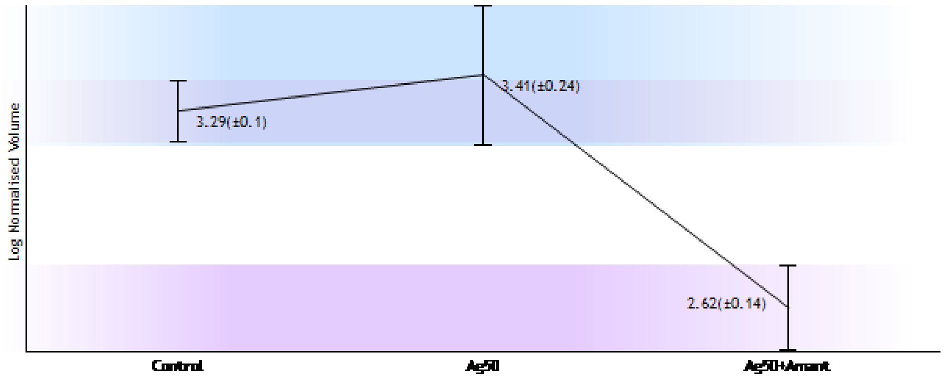

Identifier 129

Position (620, 256)

Notes

- Anova p-value ≤ 0.05
- Max fold change ≥ 1.5

Control	Ag50	Ag50+Amant
20-12-2015 2DG samples 1 IAF	20-12-2015 2DG samples 2 IAF	20-12-2015 2DG samples 29 IAF

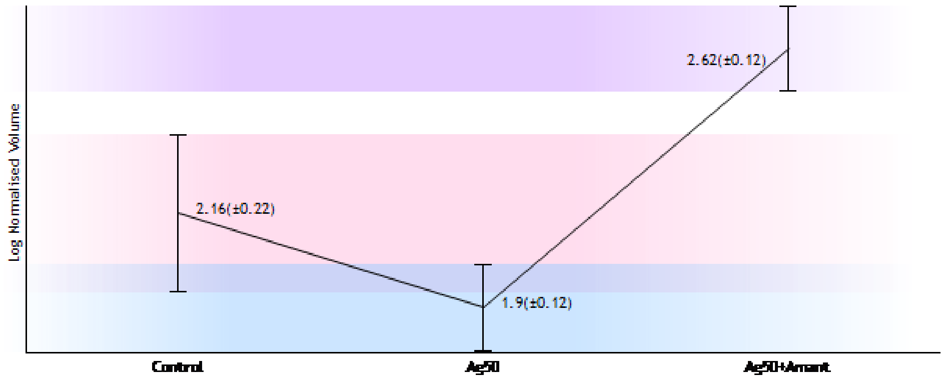

Identifier 147

Position (512, 286)

Notes

- Anova p-value ≤ 0.05
- Max fold change ≥ 1.5

Control	Ag50	Ag50+Amant
20-12-2015 2DG samples 1 IAF	20-12-2015 2DG samples 2 IAF	20-12-2015 2DG samples 29 IAF

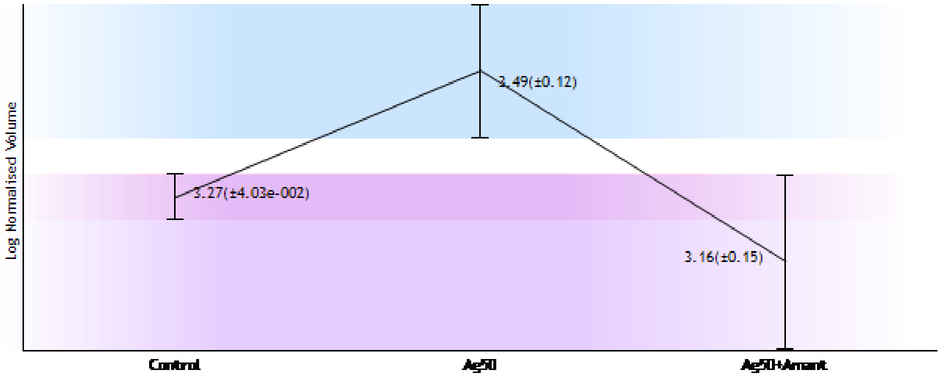

Identifier 133

Position (632, 259)

Notes

- Anova p-value ≤ 0.05
- Max fold change ≥ 1.5

Control	Ag50	Ag50+Amant
20-12-2015 2DG samples 1 IAF	20-12-2015 2DG samples 2 IAF	20-12-2015 2DG samples 29 IAF

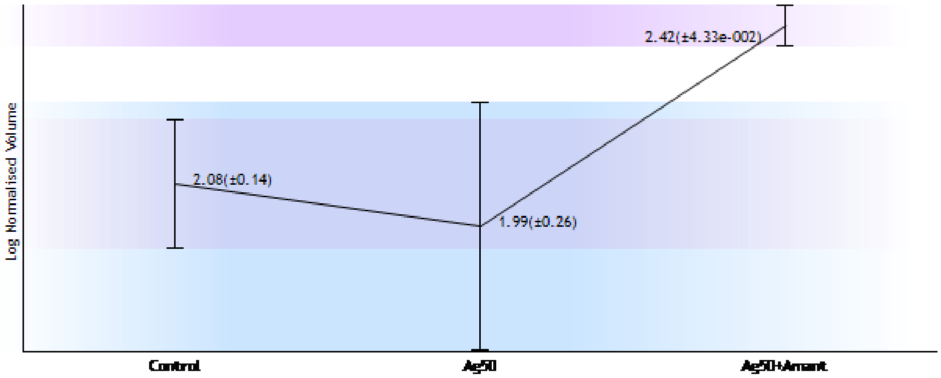

Identifier 121

Position (331, 246)

Notes

- Anova p-value ≤ 0.05
- Max fold change ≥ 1.5

Control	Ag50	Ag50+Amant
20-12-2015 2DG samples 1 IAF	20-12-2015 2DG samples 2 IAF	20-12-2015 2DG samples 29 IAF

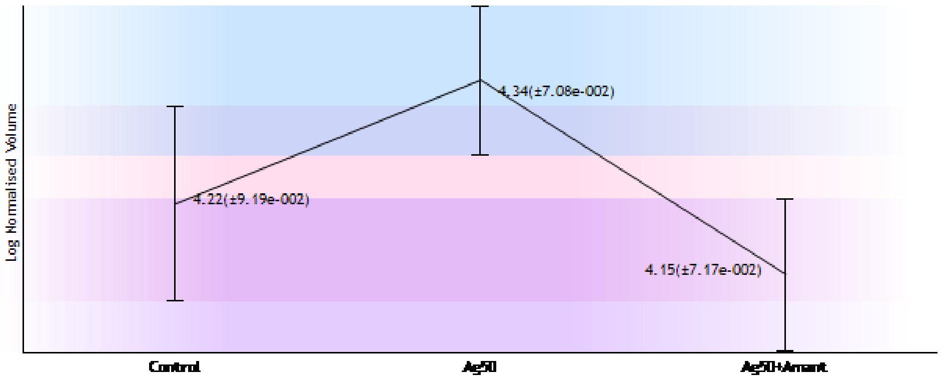

Identifier 185

Position (259, 351)

Notes

- Anova p-value ≤ 0.05
- Max fold change ≥ 1.5

Control	Ag50	Ag50+Amant
20-12-2015 2DG samples 1 IAF	20-12-2015 2DG samples 2 IAF	20-12-2015 2DG samples 29 IAF

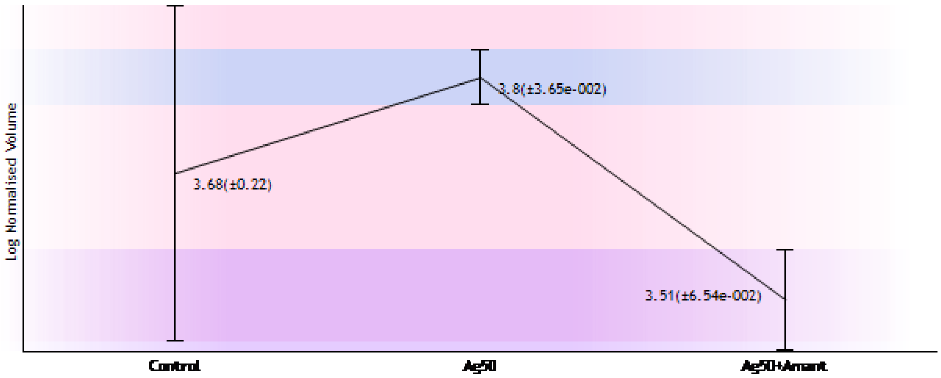

Identifier 214

Position (691, 405)

Notes

- Anova p-value ≤ 0.05
- Max fold change ≥ 1.5

Control	Ag50	Ag50+Amant
20-12-2015 2DG samples 1 IAF	20-12-2015 2DG samples 2 IAF	20-12-2015 2DG samples 29 IAF

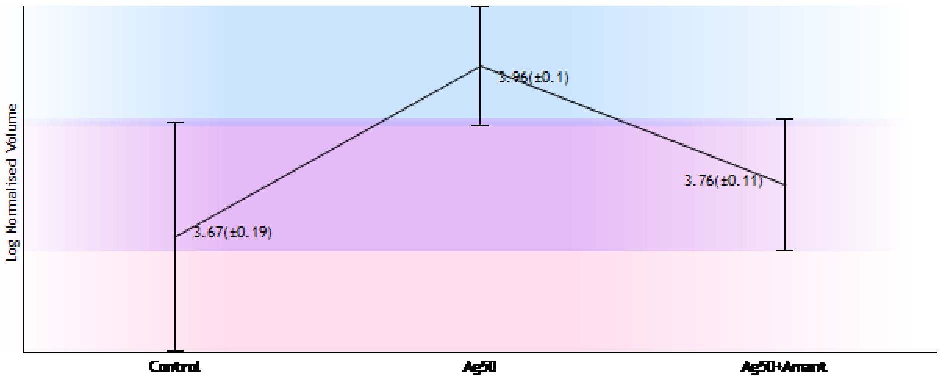

Identifier 65

Position (335, 188)

Notes

- Anova p-value ≤ 0.05
- Max fold change ≥ 1.5

Control	Ag50	Ag50+Amant
20-12-2015 2DG samples 1 IAF	20-12-2015 2DG samples 2 IAF	20-12-2015 2DG samples 29 IAF

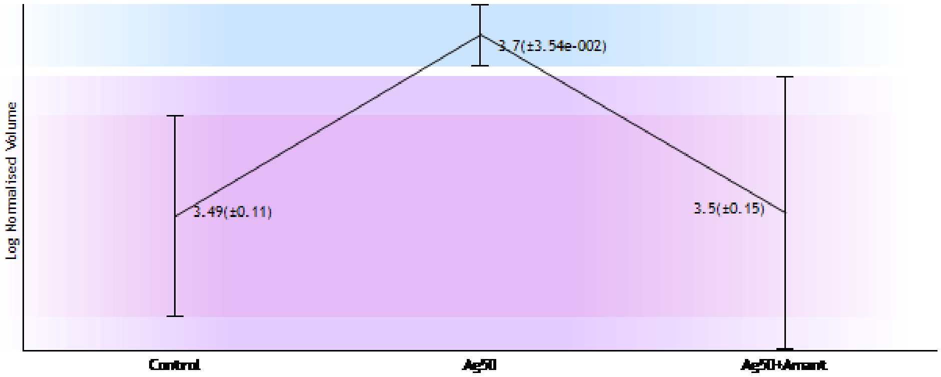

Identifier 40

Position (307, 156)

Notes

- Anova p-value ≤ 0.05
- Max fold change ≥ 1.5

Control	Ag50	Ag50+Amant
20-12-2015 2DG samples 1 IAF	20-12-2015 2DG samples 2 IAF	20-12-2015 2DG samples 29 IAF

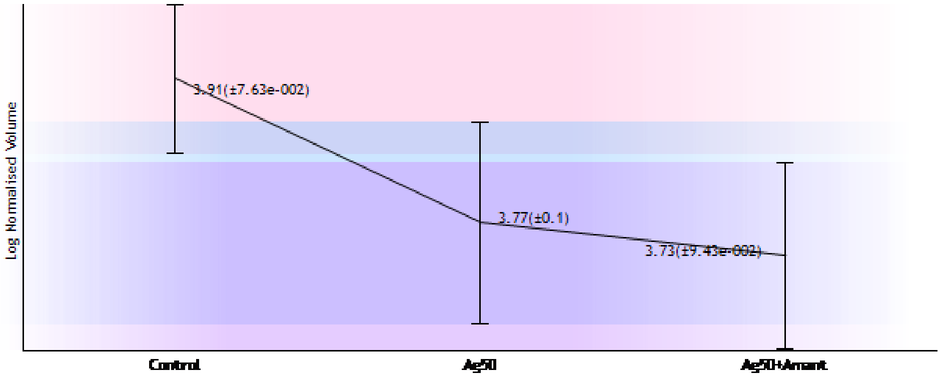

Supplement: S2 File — (PDF) [file pone.0205765.s003.pdf]
